# Multistep catalytic abiotic CO_2_ conversion to sugars through C_1_ intermediates

**DOI:** 10.1073/pnas.2514826122

**Published:** 2025-08-26

**Authors:** Nathan Soland, Jie Luo, Arifin Luthfi Maulana, Julian Feijoo, Hye-Jin Jo, Alexander M. Oddo, Yu Shan, Tianle Wang, Geonhui Lee, Jihoon Choi, Wei-Shan Huynh, Maria Fonseca Guzman, Lihini Jayasinghe, Cheng Zhu, Yao Yang, Peidong Yang

**Affiliations:** ^a^Department of Chemistry, University of California Berkeley, Berkeley, CA 94720; ^b^Department of Materials Science and Engineering, University of California Berkeley, Berkeley, CA 94720; ^c^Department of Chemical and Biomolecular Engineering, University of California, Berkeley, CA 94720; ^d^Materials and Chemical Sciences Division, Lawrence Berkeley National Laboratory, Berkeley, CA 94720; ^e^Kavli Energy NanoScience Institute, Berkeley, CA 94720

**Keywords:** CO_2_ conversion, electrocatalysis, photocatalysis, sugar synthesis

## Abstract

Carbon dioxide (CO_2_) to multicarbon (C_n_) upgrading for commodity chemicals, fuel production, or artificial food synthesis using renewable energy input is a golden target for researchers in sustainable carbon emission reduction. Here, we explore and analyze a flexible modular roadmap for the task, utilizing sequential electro-, photo-, and organocatalysis to develop a strategy for CO_2_ conversion using the key and elusive formaldehyde precursor of interest for sugar generation. We study the electrochemical carbon dioxide reduction reaction to methanol in a flow cell and its discontinuous photooxidation to formaldehyde (PMOR) with excellent selectivity. Utilizing a highly active *N-*heterocyclic carbene catalyst enables tunable generation of C_4_–C_6_ aldoses without undesirable byproducts, with carbon conversion yield reaching 60 to 80% for desired pentose, tetrose, and triose product mixtures and over 20% for hexose. This approach presents a roadmap for CO_2_ valorization, aiming to bridge carbon waste streams with sustainable sugar synthesis and opening broad avenues for green chemical production.

## Significance

Generating useful chemicals using renewable energy, known as Power-to-X, will require exploration of a variety of pathways to enable conversion of simple precursors, including wasteful greenhouse gases, into products of arbitrary complexity. Carbohydrates are valuable targets for carbon dioxide conversion, as nature has demonstrated through the multitude of applications of sugars in biological energy storage, structure, signaling, and more. However, the scalable abiotic assembly of single-carbon species from carbon dioxide to multicarbon products is challenging. Here, we showcase and evaluate a strategy synthesizing and directly assembling formaldehyde into simple carbohydrates, including life-sustaining monosaccharides, using the organocatalysts known as *N*-heterocyclic carbenes. This strategy may help inform future directions toward carbon dioxide valorization.

There is a surplus of carbon dioxide (CO_2_) emissions and a looming deficit of petroleum-derived fuels. Catalytic conversion of CO_2_ to valuable chemicals (such as carbon monoxide, methanol, formic acid, acetate, and ethanol) with selectivity and high energy efficiency represents a promising approach to address both problems simultaneously (1). Carbohydrates are an immensely valuable target product. Producing artificial sugars directly without extraneous crop components (e.g., structural carbohydrates) and associated externalities has been explored to address the world’s food requirements (2, 3). Approximately half of the world’s caloric needs can be satisfied by carbohydrates (2, 4). If deemed unsuitable for human consumption, these artificial sugars may be converted to other valuable molecular targets such as fuels, via fermentation, or pharmaceuticals and plastics by chemical upgrading or microbial manufacturing (1, 5).

**Fig. 1.**
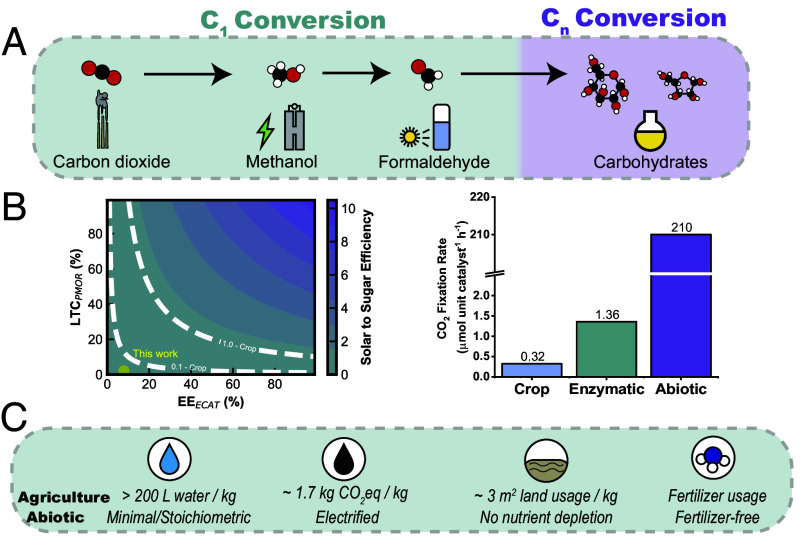
Overview of the abiotic CO_2_ to sugars strategy. (*A*) Scheme indicating the modular sequential transformation of CO_2_ to saccharides through methanol-derived formaldehyde. (*B*) Estimated theoretical solar-to-sugar energy efficiency, defined as the products of estimated solar-to-power, electrocatalytic (EE_ECAT_), photocatalytic (LTC_PMOR_) (if applicable), and C_1_ to C_n_ efficiencies, not assessing penalties for possible separation and purification steps; and carbon fixation rate/productivity in comparison to biotic strategies including conventional agriculture (represented by maize) and a landmark chemoenzymatic strategy from Cai et al. (*SI Appendix, Discussion S1*) (7). (*C*) Some negative consequences of conventional agriculture that might be mitigated using chemical strategies for sugar synthesis. Estimated water consumption, CO_2_ equivalent emissions, and land occupancy required for growth of 1 kg of maize as thoroughly calculated by Poore et al. (4).

Artificial CO_2_-to-sugar strategies explored may, for convenience, be classified as biotic (including chemoenzymatic) and abiotic (6–8). Although biological processes are often highly efficient in individual steps, in total, the real photosynthesis of biomass falls short of ideal solar-to-chemical conversion efficiency, generally only approaching 1% and more frequently on the order of 0.1% (Fig. 1*B* and *SI Appendix, Discussion S1*) (9, 10). Metabolic engineering and chemoenzymatic pathways have been broadly explored for improving CO_2_ conversion to carbohydrates, taking advantage of two-electron reduction pathways for CO_2_ fixation or existing thermocatalytic technologies combined with the powerful stereoselectivity of enzymatic reactions to produce carbohydrates (7, 11–15). Many of these approaches have preliminarily been assessed for technoeconomic feasibility and present unique solutions to accessing either metabolically relevant or chemically useful carbohydrate products. However, these chemoenzymatic concepts often suffer from many of the same challenges of biology, including low titer of product, slow turnover, costly enzymatic engineering and purification, and constraints imposed by the availability of naturally existing metabolites. High-rate carbon conversion in a minimal number of abiotic steps, even at lower stepwise efficiency and selectivity, may be comparable to the solar-to-chemical conversion yields of conventional crops and offer scalability potential (Fig. 1*B*) (2, 16). Unlike agriculture, catalytic carbon dioxide conversion powered by electricity would also potentially circumvent dependence on resource-intensive inputs with negative environmental impacts (Fig. 1*C*) such as high water consumption, land erosion, soil nutrient depletion, and fossil fuel-powered agricultural machinery (4). To this end, several powerful chemoenzymatic strategies have been advanced. However, significant research advancements are required to optimize each catalytic step in CO_2_ upgrading to carbohydrates.

Here, we explored the development of a constrained strategy to convert CO_2_ to CH_2_O focusing exclusively on potentially renewably powered electro- or photocatalysis followed by low-temperature, highly active organocatalytic conversion to C_n_ monosaccharides (C_3-6_) (Fig. 1*A*). We selected state-of-the-art catalysts that have demonstrated potential in producing the relevant intermediate products that provide access to reactive formaldehyde, which can be condensed to carbohydrates. In this work, we demonstrate lab-scale conversions of carbon through the C_1_ to C_n_ modules (Fig. 1*A*) to benchmark emerging catalytic technologies and to explore the feasibility of complex CO_2_ conversion in individually optimized steps. A key focus is the single-shot conversion of formaldehyde to monosaccharides via organocatalysis, in contrast to nonselective alkaline formose reactions that are commonly explored in this field.

## Results and Discussion

### Organocatalysis for Selective C_n_ Generation from Formaldehyde.

**Fig. 2.**
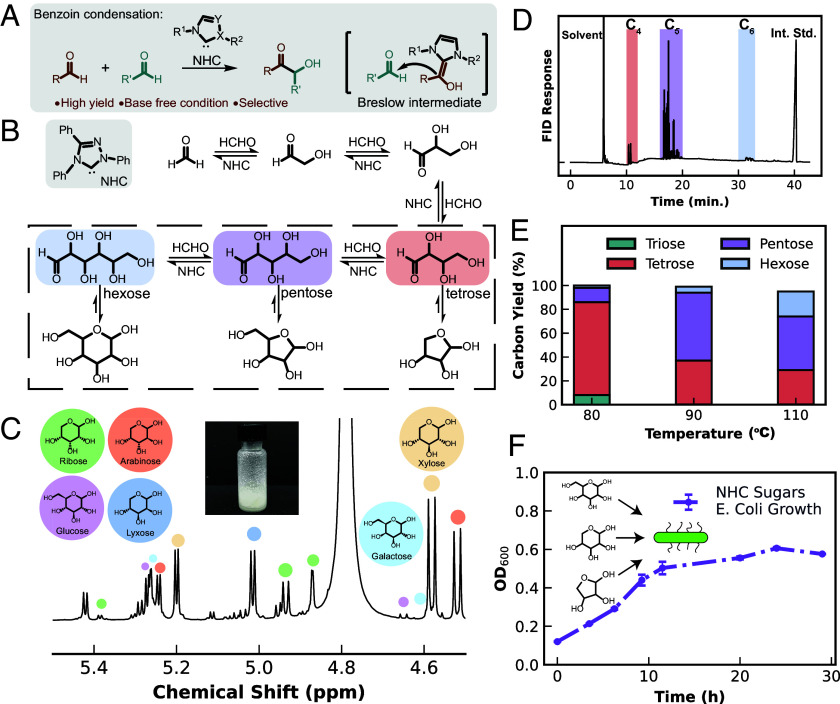
NHC catalyzes aldose generation from formaldehyde. (*A*) Mechanistic scheme of NHC-catalyzed formoin condensation. (*B*) Simplified scheme showing formoin reactions leads to the generation of stable tetrose, pentose, and hexose products. (*C*) ^1^H NMR of formoin product mixture, with identifiable anomeric chemical shifts of distinct monosaccharides annotated. Depicted as illustration are the pyranose structures of cyclic carbohydrates. (*Inset*) Image of purified sugar product from formoin reaction (*SI Appendix,* Fig. S11). (*D*) Representative gas chromatogram of acetylated pentose-optimized formoin product mixture. Groupings of similar carbohydrates appear in well-defined regions of the chromatogram and are used for quantitation. Sorbitol was used as an internal standard. (*E*) Carbon conversion yield of formaldehyde to sugars via NHC-1 for three optimized conditions, showing the isolated effect of temperature on product yield. (*F*) Optical density of growth trial using formoin sugars with a wild-type *Escherichia coli* strain.

The formose reaction has long been explored to uniquely produce complex carbohydrates from formaldehyde in alkaline conditions (17, 18). However, the autocatalytic nature of the reaction leads to kinetic instabilities and makes precise control of the product distribution infeasible. This inevitably reduces the carbon yield of carbohydrates (*SI Appendix,* Figs. S1 and S2), despite decades of research efforts (18, 19). To this end, organocatalytic benzoin-like condensation of formaldehyde catalyzed by *N*-heterocyclic carbenes (NHCs) has emerged as a promising alternative approach (Fig. 2 *A* and *B*) which has been demonstrated and discussed, notably in recent years by Bontemps et al., but typically leads to C_2_-C_4_ short-chain carbohydrate products such as glycolaldehyde and erythrose (20–24).

To extend the utility of NHC catalysis for producing versatile saccharide products, we tested various NHCs for formaldehyde reactivity (*SI Appendix,* Table S1). Enders’ carbene **NHC-1** (1,3,4-triphenyl-4,5-dihydro- l,2,4-triazol-5-ylide, Fig. 2*B*) stood out and was the major focus of our attempts due to its excellent stability under heating and high reactivity, which is essential for the generation of higher sugars. In addition, instead of using the cationic carbene precursor, which needs to be activated by the inclusion of base, we utilized an alcohol-trapped carbene precursor (1,3,4-triphenyl-4,5-dihydro-5-methoxy-1,2,4-triazole, *SI Appendix,* Figs. S3 and S4) (22), which generates the active carbene species in situ by heating during catalysis without the need for an activating base. A similar strategy has been employed in other works to help enable selective aldol condensations (20, 25). The exclusion of additional base is helpful for selective and facile generation of aldoses beyond C_3_ without isomerization to ketoses.

We systematically optimized the temperature, reaction time, and solvent to control the degree of CH_2_O condensation, aiming to generate different speciation of sugars. General trends observed include the ability to selectively generate tetrose, pentose, and some hexose sugars, which can form thermodynamically favorable furanose or pyranose structures (Fig. 2*B*). These structures exhibit thermodynamic differences among the various products, providing opportunities to control the selectivity through precise tuning of the reaction conditions (Fig. 2 *C*–*E* and *SI Appendix,* Table S1). Gas chromatography (GC) of acetylated products in conjunction with high-performance liquid chromatography (HPLC), nuclear magnetic resonance spectroscopy (^1^H NMR, ^13^C NMR), gas chromatography-mass spectrometry (GC-MS), and infrared spectroscopy (IR) confirms the presence of desirable C_5_–C_6_ sugars including lyxose, xylose, arabinose, ribose, glucose, and galactose (*SI Appendix,* Figs. S5–S11 and Table S2). In the separately optimized reactions, carbon yield of tetrose, pentose, and hexose was 78, 62, and 23% respectively, as calculated by GC analysis of the acetylated product with a sorbitol internal standard (Fig. 2 *D* and *E* and *SI Appendix,* Fig. S12 and *Discussion S2*). Control experiments without substrate and without NHC generated no quantifiable product (*SI Appendix,* Figs. S13 and S14). In total, this high-yield one-step conversion to beyond-C_3_ products is a unique feature of the nonaqueous formoin reaction using Enders’ carbene, and one we found particularly intriguing for the prospects of future CO_2_ upgrading efforts, despite the extra distillation step it may introduce for integration with aqueous carbondioxide reduction reaction (CO_2_RR) conversions (vide infra).

A thorough understanding of the formoin aldose selectivity can account for the potential formation and fate of ketoses. Once formaldehyde is converted to glycolaldehyde (C_2_H_4_O_2_), there are C_>1_ reactants available for the NHC to utilize, which may complicate the product mixture. For example, ketose generation is possible through C_2_–C_2+_ coupling and is tentatively indicated by the presence of additional peaks of intermediate retention time in the GC chromatogram (*SI Appendix, Discussion S3*). Indeed, **NHC-1** can utilize glycolaldehyde as the initial substrate, and the product chromatogram displays these intermediate peaks (*SI Appendix,* Fig. S15). This increases the product complexity, yet in the formoin reaction, we do not generally observe these species except with highly concentrated reactions. We tentatively interpret this as steric favorability of the coordination of **NHC-1** to formaldehyde to form the Breslow intermediate, suppressing the generation of the byproducts observed in the glycolaldehyde reaction and improving aldose selectivity.

We found that stabilized ketoses, such as dihydroxyacetone (DHA, C_3_ ketose) and erythrulose (C_4_ ketose), are generally terminal species for the reaction as they are not suitable substrates for the NHC compared to their aldose isomers. Notably, the nonreactivity of DHA and other ketoses suggests that branched sugars, considered toxic byproducts of uncontrolled formose chemistry, are unlikely to be generated from this system (*SI Appendix,* Figs. S1, S2, S16, and S17 and *Discussion S3*). Therefore, not only is the generation of ketose sugars less favorable, but if generated, as is always the case during condensations with an activating base present in an aqueous solvent, they are present primarily as spectators or terminal products. Very recently, the viability of a formaldehyde-based intermediate for a chemo-biological cascade to produce lactic acid via DHA was reported, supporting the increasing interest in producing viable multicatalytic processes given the ability to utilize dihydroxyacetone (15). Thus, a two-pronged mechanism for suppression of nonaldose products is achieved. To our knowledge, these experiments present the highest selectivity and yield for direct conversion of formaldehyde to pentoses/hexose reported to date and present a useful strategy toward further enhancing product selectivity.

To briefly explore the applications of the NHC formoin product, we scaled up the reaction to the gram scale and isolated the NHC-produced carbohydrate product mixture for further purification and feeding to *Escherichia coli* (*E. coli*), a model heterotrophic organism for metabolic engineering and biomanufacturing. After facile formoin product purification (*SI Appendix,* Fig. S18), 83% mass yield was obtained as a viscous yellow syrup. The formoin sugars were fed to *E. coli* as the sole carbon source (1% wt/wt) in M9 minimal media. Cell growth was confirmed by sigmoidal increase of optical density up to a stationary phase of ~0.6 with a specific growth rate of 0.138 h^−1^ (Fig. 2*F*), indicating biocompatibility of the formoin product. For comparison, *E. coli* cultured on D-glucose under identical conditions exhibited an unsurprisingly higher growth rate, with a specific growth rate of 0.347 h^−1^, reflecting the expected lower metabolic availability of the racemic mixture of primarily C_5_ sugars (*SI Appendix,* Fig. S19 and Tables S3 and S4) of the formoin reaction. Future optimization of the reaction to prioritize enantioenriched hexose sugars is reasonably expected to enhance caloric density and thus microbial suitability for bioconversion of formoin sugars.

### Electrocatalytic Methanol Generation from CO_2_.

The feasibility of controlled aldose formation from formaldehyde (CH_2_O) necessitates a catalytic system to produce the formaldehyde itself from CO_2_. Despite significant advances in heterogeneous electrocatalysis in the last decades, directing multiple sequential proton-coupled electron transfers is kinetically challenging and thus remains a lucrative outstanding research challenge for CO_2_ conversion. CH_2_O is rarely identified even as a stable minor product in CO_2_ electroreduction (CO_2_RR), limiting the feasibility of its direct isolation as a substrate for sugar conversion (6, 26).

Alternatively, CH_2_O can often be generated robustly in methanol oxidation, and reports of methanol-producing electrocatalysts are more prevalent (6, 27, 28). Therefore, we targeted a multistep catalytic strategy for CO_2_ abiotic conversion to sugars through a route involving electroreduction to methanol followed by partial oxidation to formaldehyde. For validation, we targeted CO_2_RR performance with partial current density j_MeOH_ on the order of 10 mA cm^−2^, which translates to 60 µmol methanol h^−1^ cm^−2^, multiple factors higher than typical ambient CO_2_ fixation for a leaf (29).

**Fig. 3.**
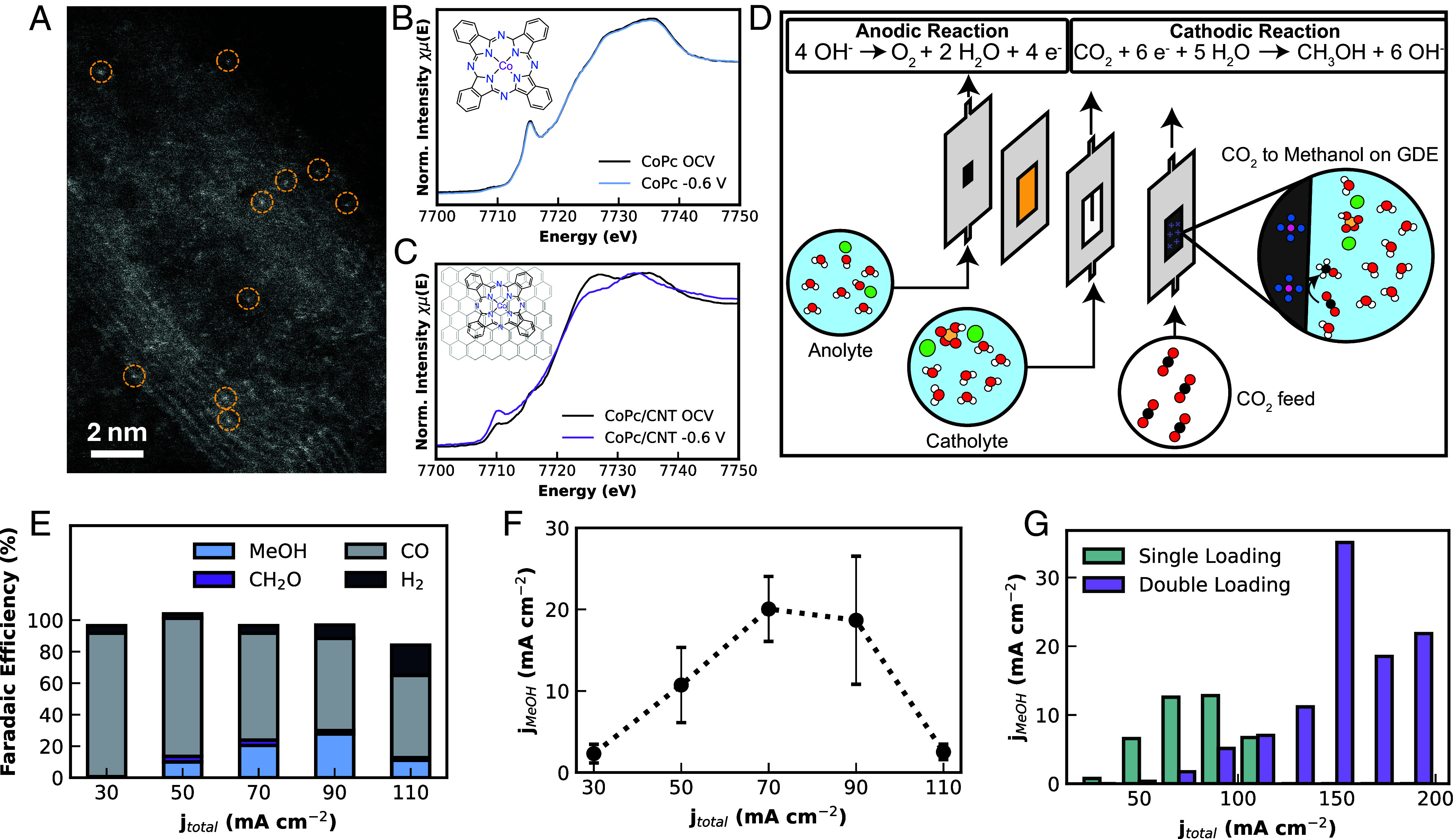
Characterization and electrocatalytic CO_2_RR performance of immobilized cobalt phthalocyanine (CoPc) on carbon nanotubes (CNTs). (*A*) HAADF-STEM image showing single-atom cobalt sites isolated on CNTs. (*B* and *C*) *Operando* Co K-edge XANES of (*B*) CoPc and (*C*) CoPc/CNT catalyst in an undivided electrochemical cell with and without bias under CO_2_ flow. The stark reduction of the 7,715 eV feature on CNT suggests monodispersity. Additionally, the spectrum of CoPc/CNT changes under bias, indicating its electroactive nature, whereas CoPc does not change due to its insulated nature. (*D*) Schematic of the electrochemical flow cell and associated reactions. (*E*) FE of major products from CO_2_ under galvanostatic conditions. (*F*) Productivity of catalyst as partial current density to methanol. Error bars are SD of three measurements. (*G*) Improved activity after increasing total Co loading on CNTs.

We selected cobalt phthalocyanine on carbon nanotube support (CoPc/CNT) as the CO_2_RR catalyst (*SI Appendix,* Fig. S20) due to growing recognition of its viability for generating beyond-two-electron C_1_ products such as methanol (6 e^−^) with moderate faradaic efficiency (FE), a metric of electron utilization efficiency for a desired product (*SI Appendix, Discussion S4*) (26, 30, 31). Following precedent, the CoPc/CNT catalyst was readily synthesized by thorough cosonication of the precursors in *N,N*-dimethylformamide (*SI Appendix, Discussion S5*) (30). Its structure and activity were validated by electron microscopy, X-ray spectroscopy, and electrochemical characterizations (Fig. 3 *A*–*C* and *SI Appendix,* Figs. S21–S28). Notably, high-angle annular dark-field scanning transmission electron microscopy (HAADF-STEM) imaging identified the presence of single-atom cobalt sites (Fig. 3*A*) with limited aggregation and few powder crystallites remaining.

X-ray absorption near-edge structure (XANES) displays the electronic fingerprint of adsorbed CoPc species as distinct from the native CoPc precursor, especially the features at 7,715 eV associated with molecular distortion caused by adsorption to the CNT substrate (Fig. 3 *B* and *C* and *SI Appendix,* Figs. S24 and S25) (31–33). While crystallites of CoPc show negligible spectroscopic change under the CO_2_RR at −0.6 V vs. RHE, *operando* XANES of CoPc/CNT shows a decrease in the white-line intensity at ~7,725 eV, an increase in the pre-edge intensity at ~7,710 eV and a slight absorption edge shift to lower energy values, corresponding to the electroreduction of Co sites to a lower average oxidation state during CO_2_RR. Extended X-ray absorption fine structure (EXAFS) analysis suggests that most cobalt was present without CoPc–CoPc interactions, while also showing reduced molecular degradation compared to the related catalyst copper phthalocyanine, underscoring the promising stability of the CoPc system (*SI Appendix*, Fig. S26 and Tables S5 and S6). X-ray photoelectron spectroscopy (XPS) analysis further indicated the presence of possible strain effects, indicated by N 1s peak splitting, likely induced by CoPc interaction with CNTs in the synthesized catalyst (*SI Appendix,* Fig. S27) (32). Collectively, these characterizations indicate an immobilized species that is chemically distinct from the unhybridized CoPc, which is known to terminate CO_2_RR at CO, only a net two-electron process (34).

We then tested the catalyst for methanol generation (35). We utilized a gas diffusion electrode to apply potentiostatic and galvanostatic control and screened a range of conditions and total current densities (Fig. 3*D* and *SI Appendix,* Figs. S29–S37) in pursuit of high-rate CO_2_ conversion. Utilizing 0.55 mg cm^−2^ of CoPc/CNT (*SI Appendix,* Fig. S37), we achieved a peak FE_MeOH_ of 27% at an average approximate potential of –0.95 V vs. RHE, one of few, though recently growing, reports of direct CO_2_RR to methanol in the flow cell (31, 32, 36, 37). Peak j_MeOH_ reached 20 mA cm^−2^ or 0.124 mmol methanol h^−1^ cm^−2^ (Fig. 3 *E* and *F*). Previous work has emphasized that CoPc solubility in the loading step can limit total catalyst loading on CNTs (33). Therefore, we increased the mass loading of cobalt from 0.27 to 0.46 wt% on CNTs by two sequential CoPc loadings, enabling an improved peak j_MeOH_ of 35 mA cm^−2^ (0.216 mmol h^−1^ cm^−2^) at an average cathodic potential of approximately −1.1 V vs. RHE, exceeding the benchmark goal of 10 mA cm^−2^ (Fig. 3*G*). Although this efficiency approaches other high-yielding reports in the literature, a significant challenge remains for the stability of the system. To this end, we tested the durability of the flow cell under extended operation. A slight decrease in FE_MeOH_ to 17% was shown over the course of a 4-h period at a total current density of 90 mA cm^−2^ (*SI Appendix,* Fig. S36).

We note that in addition to methanol, formaldehyde is a direct product of CO_2_RR on CoPc/CNT (Fig. 3*E*), consistent with previous findings and the hypothesized mechanism of CO reduction to methanol (26, 38). In our trials, FE_CH2O_ does not exceed 5%, which is inadequate for attempting isolation and conversion to sugars but suggests future potential for tuning immobilized molecular catalyst systems for direct CO_2_ to CH_2_O conversion. Collectively, these results reinforce the ideas that catalyst loading and associated kinetic factors govern the efficiency of a complex catalytic system such as CoPc/CNT (33, 36, 37, 39) and enable a path toward more efficient CO_2_ conversion to reactive C_1_ products for future sugar generation. Numerous groups are developing efficient scale-ups of the molecular system, which may eventually produce sustainable methanol at adequate concentrations for distillation and purification.

### Photocatalytic Conversion of Methanol to Formaldehyde.

After electrochemical methanol production, conversion to the requisite formaldehyde for subsequent sugar synthesis must be achieved by exquisitely selective catalysis. To address this challenge, we turned to photocatalytic methanol dehydrogenation (PMOR), which integrates methanol oxidation and the hydrogen evolution reaction (HER) in a single step, offering a sustainable method for processing the product stream from electrocatalysis. Within the class of semiconductor photocatalysts, zinc indium sulfide (ZIS), a ternary chalcogenide, has attracted attention due to its low toxicity, suitable band edges for alcohol dehydrogenation, and high stability. However, ZIS synthesized by conventional hydrothermal or solvothermal methods often exhibits complex morphologies and low activity toward PMOR (40–42).

**Fig. 4.**
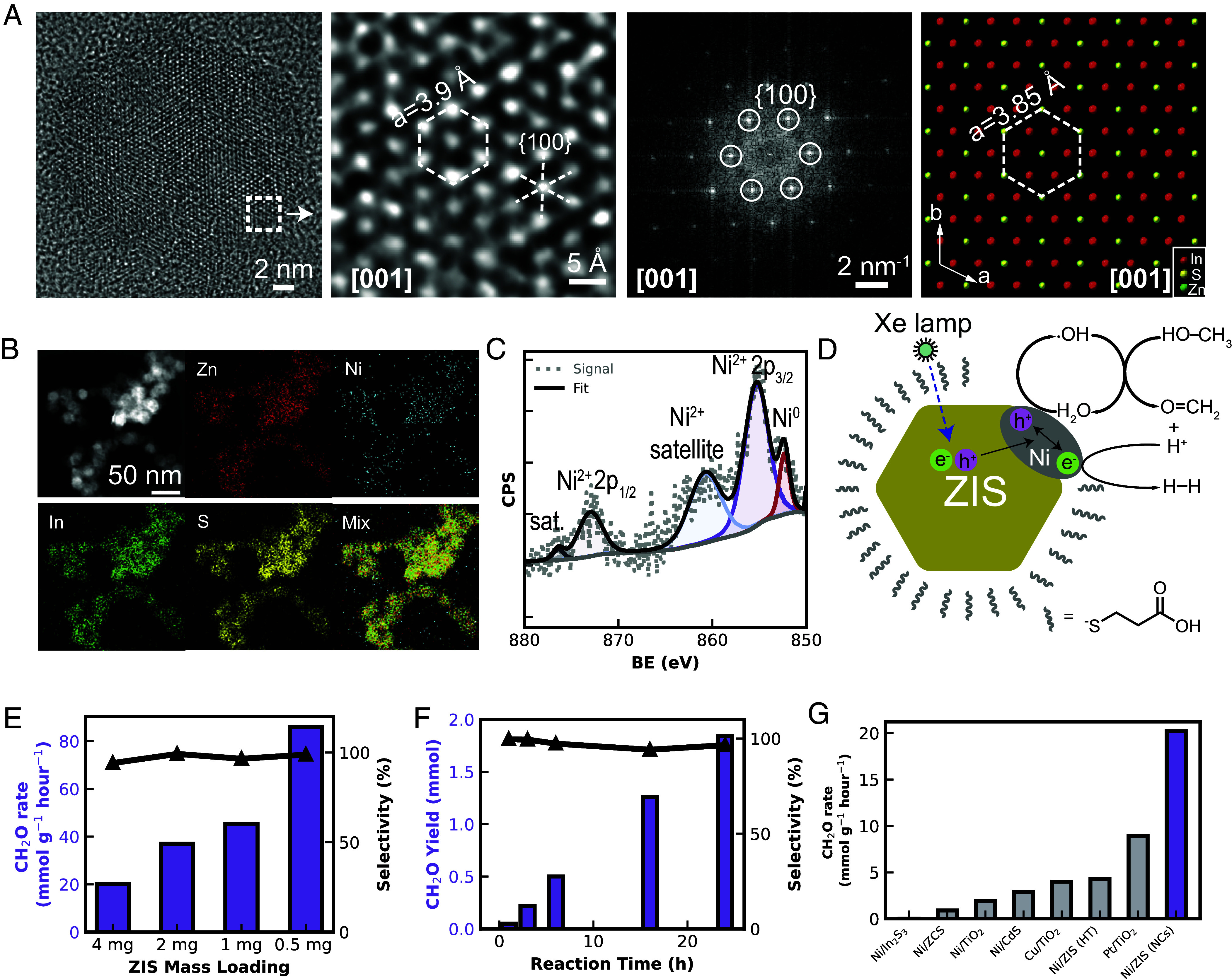
Characterization and photocatalytic MOR performance of ZIS nanocrystals. (*A*) Atomic resolution HR-TEM and corresponding Fourier transform and crystal model highlighting the hexagonal lattice structure of the ZIS nanocrystals close to the [001] zone axis. (*B*) STEM-EDX mapping indicating a homogeneous distribution of zinc, indium, sulfur, and nickel cocatalyst (2% loading by mass). (*C*) Ni XPS postcatalysis demonstrating the presence of metallic nickel after successful photodeposition. (*D*) Schematic showing the carrier diffusion, charge separation, and Ni-dependent dehydrogenation of methanol to formaldehyde. (*E*) Productivity and selectivity of Ni/ZIS catalyst as a function of total mass loading. Lower loading leads to higher specific activity, but lower total productivity. Reaction conditions: 4 mL MeOH:2 mL H_2_O 4 mg catalyst and 16 h illumination Xe lamp. (*F*) Productivity and selectivity of catalyst over a 24-h trial. (*G*) Comparison of formaldehyde generation rate for various photocatalysts measured in-house under identical conditions.

To achieve suitable activity, we optimized the colloidal synthesis of ZIS by employing the hot injection of sulfur into a mixture of zinc and indium chloride precursors to form nanocrystals with well-defined crystalline morphology (*SI Appendix,* Fig. S38) (43). The resulting ZIS NCs were characterized primarily as hexagonal nanoplates with an average width of approximately 20 nm (*SI Appendix,* Fig. S39). High-resolution transmission electron microscopy (HRTEM) images, along with Fourier transform analysis, confirmed the well-ordered hexagonal structure of ZIS, which was also supported by powder X-ray diffraction patterns (*SI Appendix,* Fig. S40). The HRTEM image of ZIS shows the lattice parameter of 3.9 Å, which is consistent with theoretical values of 3.85 in the crystal model on the same [001] zone axis (Fig. 4*A* and *SI Appendix,* Fig. S41).

Surface modification of semiconductor photocatalysts with a cocatalyst significantly accelerates the HER process associated with photocatalytic alcohol dehydrogenation, thereby improving efficiency and stability (44). Ni, known for its high HER activity, low cost, and high abundance, was introduced onto our developed ZIS nanocrystals through an in situ photodeposition method (45). The resulting Ni/ZIS NCs exhibited uniform elemental distribution as confirmed by STEM energy dispersive X-ray spectroscopy mapping, indicating successful deposition of Ni (Fig. 4*B* and *SI Appendix,* Fig. S42). Postdeposition XPS revealed the presence of Ni^0^, which may function as an active site for methanol dehydrogenation, and photodeposited Ni particles are visible by contrast in TEM (Fig. 4 *C* and *D* and *SI Appendix,* Figs. S43 and S44) (46). To probe the mechanistic role of Ni, we performed electron paramagnetic resonance spectroscopy, which showed strong signal enhancement of DMPO-trapped hydroxyl radical (HO·) species in aqueous methanol solutions containing the Ni cocatalyst. The HO· radicals are expected reactive species generated from water on the nickel surface during the photocatalytic process, which further leads to the generation of formaldehyde from methanol through the hydroxymethyl radical (·CH_2_OH) (*SI Appendix,* Figs. S45) (45, 47, 48).

After successfully engineering the catalyst nanostructure and cocatalyst loading, we investigated the catalyst performance in an air-free aqueous methanol solution under simulated sunlight using a Xe lamp (*SI Appendix,* Figs. S46–S49 and *Discussion S6*). The Ni/ZIS NCs demonstrated high conversion rates and near-unit selectivity in trials of photocatalytic methanol dehydrogenation. Optimization of catalyst mass loading indicated an increasing trend in activity when the mass loading was decreased, leading to a productivity exceeding 80 mmol g cat^−1^ h^−1^ approaching the benchmark results obtained with advanced oxide-based photocatalysts, such as engineered Pt/TiO_2_ in a highly dilute solution (164 mmol g cat^−1^ h^−1^) (Fig. 4 *E*–*G* and *SI Appendix,* Fig. S50) (49). The high production rate can be attributed to the prevention of nanoplate aggregation, which improves the specific activity by increasing Ni/ZIS surface area. For a potentially photosynthetic pathway of CO_2_ to sugars, the ZIS NCs are thus an excellent component to fill in the technology gap of direct CO_2_ conversion to formaldehyde, allowing multiple handles for tunability that may be suitable to treat the engineering task of postelectrolysis methanol conversion in a sustainable manner.

### Integration and Scalability of Each Module.

**Fig. 5.**
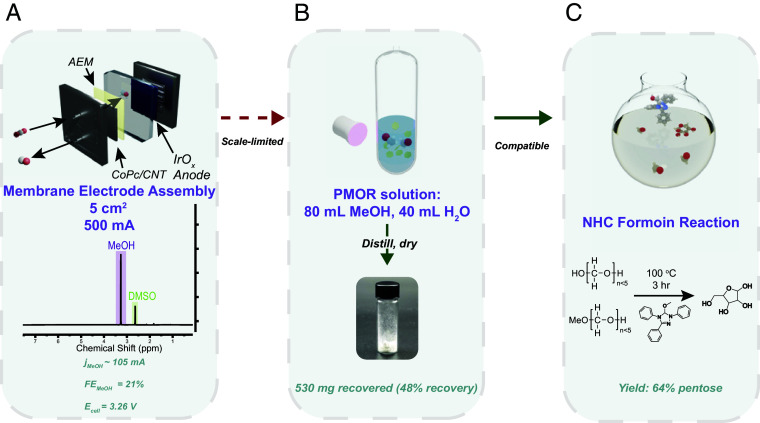
Process assessment and scale-up. (*A*) Utilization of a MEA for high-rate methanol production from CO_2_RR. (*B*) Photocatalytic reaction scale-up for gram-scale formaldehyde synthesis. (*C*) NHC reaction and utilization of methanol-derived anhydrous formaldehyde.

The multicatalytic optimization and benchmarking presented above represent a critical step in addressing the greater challenge of establishing an abiotic CO_2_-to-sugars framework. To explore the process viability at the lab scale, we scaled up each of the reactions to match the requirements of the formoin reaction. We translated the CoPc system to a 5 cm^2^ membrane electrode assembly (MEA) in CO_2_RR, improving full cell voltage performance by eliminating catholyte solution resistance (Fig. 5*A* and *SI Appendix,* Fig. S51). On a 5 cm^2^ electrode at 100 mA cm^−2^, we achieved a FE of 21% at a full cell voltage of 3.26 V, for a total partial current of 105 mA to methanol. This translates to 0.65 mmol MeOH h^−1^, which is approximately equivalent to 0.025 mL h^−1^. However, this rate is not suitable for preparative scale methanol production in the laboratory environment, suggesting a technology gap remains for nonthermochemical CO_2_ conversion to methanol. We expect ongoing CO_2_RR engineering developments will soon bridge this gap and help connect the methanol production and oxidation processes through a distillation-concentration step.

We then turned to the photocatalytic scale-up depicted in Fig. 5*B* to generate formaldehyde at the gram scale to produce carbohydrates from non-CO_2_-derived (commercial) methanol. This closes the gap for full integration of steps two and three of the proposed abiotic route for CO_2_ conversion (Fig. 5 *B* and *C*). The 20-fold scale-up was optimized in 120 mL 2:1 ratio of methanol to aqueous ZIS suspension. Application of 370 nm UV lamp enabled generation of 1 L of H_2_ gas over the course of 72 h, corresponding to 1.1 g of formaldehyde produced. As noted previously, the carbene catalysts are ostensibly deactivated by the presence of water or alcohol. As such, an anhydrous product is desired. Separation of formaldehyde as a dilute species from an aqueous mixture was achieved by directly drying the sample (at approximately 10% recovery yield), but improved recovery at the lab scale was achieved by distilling the lighter boiling species (monomers and oligomers of formaldehyde and excess methanol) and capturing the higher boiling fraction (110 to 140 °C). This fraction was then dried and yielded 48% carbon recovery as a dry white powder resembling commercial paraformaldehyde (Fig. 5*B* and *SI Appendix,* Fig. S52), which could be used for the organocatalysis step directly as described above. Use of the recovered formaldehyde product with **NHC-1** at 100 °C in 3 mL dioxane produced 64% carbon yield of pentose, balance tetrose, supporting that commercial PF is not necessary for formoin conversion (Fig. 5*C*). The sugar-trapped **NHC-1** is also amenable to recycling, and satisfactory aldose production can also be achieved in ambient atmospheric conditions without using argon (*SI Appendix*, Figs. S53 and S54 and Table S7). These steps toward improving practicality of the process illustrate the remarkable opportunities for C_1_ valorization with this class of catalyst. Scaling of electrocatalytic reduction of CO_2_ is proceeding rapidly and bodes well for near-term improvement in preparative methanol generation (*SI Appendix*, Fig. S55).

## Conclusion

In this work, we assessed the performance of several promising catalytic technologies in the context of a roadmap for valorizing carbon dioxide to aldose sugars through CO_2_RR liquid products. There have been significant advances recently in the subfields of catalysis that enable us to envision a renewable CO_2_ to sugar process. However, as with any nascent technology, some limitations remain, and competitive processes should be explored in parallel. In particular, the electrocatalytic system suffers from at least three common challenges of CO_2_RR: limited FE of the molecular catalyst which inhibits the energy efficiency of the process; high overpotential of the system, which similarly enforces an immediate ceiling on energy efficiency, and low production rate which is also intrinsic to electrochemical reactions. Despite demonstrably high production rates and selectivity, the total photocatalytic formaldehyde yield is a small fraction of total methanol in the reactor, suggesting low carbon yield of the process. However, as we have demonstrated, the solvent can be readily recycled, improving the outlook for isolating anhydrous formaldehyde (as paraformaldehyde) for subsequent reactions, such as the NHC formoin reaction.

Despite current technical limitations, one consistent strength of abiotic catalytic strategies lies in the potential for rapid conversion of carbon products compared to natural and biocatalytic strategies. Additionally, conventional thermal catalysis to generate formaldehyde is a much more mature process with high energy efficiency in comparison to the presented electro/photocatalytic process (*SI Appendix, Supplementary Note 1*). If powered with renewable energies, and if the hydrogen source for CO_2_ hydrogenation were from “green hydrogen,” this is also a viable alternative that would couple favorably with the formoin chemistry shown here.

Though the current generation of synthetic sugars for petrochemical displacement by biomanufacturing is not economically competitive with conventional agricultural sugars, the finitude of petroleum resources imposes the need to develop parallel and sustainable alternatives for important carbon feedstocks. We expect that the valuable one-pot formoin conversion of formaldehyde to tetrose, pentose, and hexose should be investigated for CO_2_ valorization as a preferable paradigm over the formose reaction. The clear value of crosstalk between adjacent fields of catalysis and engineering, as explored here, will inform practical advantages of strategies for CO_2_ recycling and limitations thereof. The goal of renewably synthesized sugars using electro- or photochemical processes remains a scientific challenge worthy of discussion, demonstration, and improvement.

## Materials and Methods

Detailed information regarding all experimental methods is provided in the *SI Appendix*. Briefly, NHCs were prepared according to a modified literature procedure. Sugar generation from formaldehyde was performed by addition of the alkoxy NHC precursor, the paraformaldehyde, and solvent to a Schlenk flask under inert atmosphere and heat. Product quantitation was performed by acetylation and analysis on a Shimadzu GC-2010 Plus by comparison to a sorbitol internal standard. Sugar identity was further investigated using an Agilent Infinity II HPLC equipped with an Aminex HPX-87H column. Mass spectra were obtained with an Agilent 7890B GC/5977A MSD system. NMR spectroscopy was performed on a Bruker AV-600 or NEO-500 spectrometer at the Pines Magnetic Resonance Center, UC Berkeley. Electrodes for CO_2_ reduction were prepared by cosonicating commercial cobalt phthalocyanine with multiwall carbon nanotubes in *N,N,*-dimethylformamide (DMF), washing with DMF before drying and resuspension in an ethanol/Nafion dispersion and drop-casting onto carbon paper with a gas diffusion layer. Electrolysis was performed in a custom flow cell, and products were characterized by GC, proton NMR, and formaldehyde-specific colorimetric assay. ZIS NCs were colloidally synthesized. The photocatalysis was performed as a water/methanol suspension of ZIS exposed to a 300 W Xe lamp (or 370 nm Kessil lamp) in a quartz tube under inert atmosphere. The headspace gas was used to measure hydrogen yield, and formaldehyde selectivity was determined by proton NMR.

## Supplementary Material

Appendix 01 (PDF)

## Data Availability

All study data are included in the article and/or *SI Appendix*.
