# Supplementary material for: Multistep catalytic abiotic CO2 conversion to sugars through C1 intermediates
Source: Proc Natl Acad Sci U S A. 2025 Aug 26;122(35):e2514826122. doi: 10.1073/pnas.2514826122 (PMC12415257; doi:10.1073/pnas.2514826122)
Supplement: Supplementary file 1 — Appendix 01 (PDF) [file pnas.2514826122.sapp.pdf]

PNAS

Supporting Information for
Multi-step Catalytic Abiotic CO_2 Conversion to Sugars Through C_1 Intermediates

Nathan Soland[a,1], Jie Luo[a,1,2], Arifin Luthfi Maulana[b], Julian Feijoo[a], Hyejin Jo[c], Alexander M. Oddo[a,d], Yu Shan[b], Tianle Wang[a], Geonhui Lee[a,3], Jihoon Choi[b], Wei-Shan Huynh[a,4], Maria Fonseca Guzman[a], Lihini Jayasinghe[a], Cheng Zhu[b,d,e], Yao Yang[a,5], Peidong Yang[a,b,d,e,6]

[a]Department of Chemistry, University of California Berkeley; Berkeley, CA 94720, USA
[b]Department of Materials Science and Engineering, University of California Berkeley; Berkeley, CA 94720, USA
[c]Department of Chemical and Biomolecular Engineering, University of California, Berkeley, CA 94720, USA
[d]Materials Sciences Division, Lawrence Berkeley National Laboratory; Berkeley, CA 94720, USA
[e]Kavli Energy NanoScience Institute; Berkeley, CA 94720, USA

[1]These authors contributed equally to this work
[2]Current address: Department of Chemistry, Zhejiang University, Hangzhou, Zhejiang, China 310058
[3]Current address: Department of Chemical and Biological Engineering, Seoul National University, Seoul, Republic of Korea 08826
[4]Current address: Department of Chemistry, Northwestern University, Evanston, IL 60208, USA
[5]Current address: Department of Chemistry and Chemical Biology, Cornell University, Ithaca, NY 14850, USA
[6]Corresponding author: Peidong Yang

Email: p_yang@berkeley.edu

This PDF file includes:

Supporting text
Figures S1 to S55
Tables S1 to S7
SI References

Supporting Information Text

Materials and Methods

NHC catalyzed formoin condensation for selective carbohydrate generation from formaldehyde

NHC synthesis:

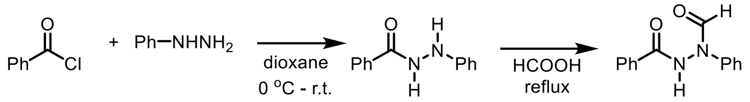

A solution of benzoyl chloride (8.8 mL, 76 mmol, in 30 mL dioxane) was added dropwise slowly to a solution of phenylhydrazine (15 mL, 150 mmol, in 30 mL dioxane) at 0 °C. The mixture was stirred at room temperature for 0.5 h. The precipitate of phenylhydrazine hydrochloride was filtered off. The filtrate was added to 100 mL hexanes and the precipitate of 2-benzoylphenylhydrazine was collected by filtration and washed several times with hexanes (3 × 30 mL). The product was dried under vacuum and formic acid (75 mL) was added. The resulting mixture was refluxed for 6 h after which the excess of formic acid was evaporated to dryness. The residue was dissolved in acetone and precipitated with the addition of large excess of hexane. The white solid was filtered off and dried to afford 1-formyl-2-benzoylphenylhydrazine, which was pure enough for further utilization.

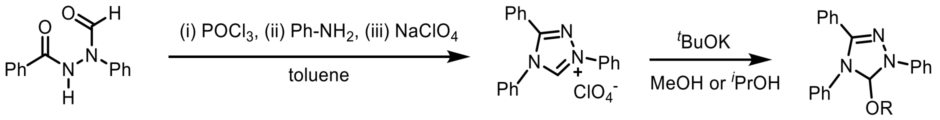

Phosphorus oxychloride (0.22 mL, 2.5 mmol) was added dropwise to 1-formyl-2-benzoylphenylhydrazine (0.6 g, 2.5 mmol) in toluene (5 mL). The reaction mixture was stirred for 20 min at room temperature and then aniline (0.28 mL, 2.5 mmol) was added. The resulting mixture was stirred at room temperature for 4 h, following which the solution was refluxed overnight. Then the upper toluene solution was poured off, and 0.2 g activated carbon and 60 mL water was added and heated at 90 °C for 0.5 h. The resulting hot solution was filtered quickly and sodium perchlorate (0.46 g, 3.7 mmol) was then added to the solution. The resulting white precipitate was filtered off, washed with water, a mixture of hexane and isopropanol (v/v = 3/1) and subsequently dried to afford NHC perchlorate precursor.
^{t}BuOK (0.23 g, 2.1 mmol) and NHC perchlorate precursor (2 mmol) was mixed in anhydrous methanol or isopropanol (16 mL) under N_2 atmosphere and stirred at room temperature overnight. Then the solvent was evaporated in vacuo, and Et_2O (16 mL) was added to dissolve the resulting solid. The precipitate was filtered and the filtrate was evaporated to dryness. The resulting fairly pure NHC was then transferred into glovebox for catalytic reactions or further purified by recrystallization in MeOH.

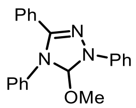

5-methoxy-1,3,4-triphenyl-4,5-dihydro-1H-1,2,4-triazole: ^{1}H NMR (500 MHz, C_6D_6) δ 7.60 – 7.46 (m, 4H), 7.25 – 7.14 (m, 2H), 6.98 – 6.87 (m, 5H), 6.86 – 6.76 (m, 3H), 6.73 (t, J = 7.3 Hz, 1H), 6.48 (s, 1H), 2.93 (s, 3H). ^{13}C NMR (126 MHz, C_6D_6) δ 145.21, 142.76, 140.78, 129.53, 129.30, 129.11, 128.71, 128.67, 128.35, 125.06, 123.21, 120.58, 113.58, 101.09, 46.95.

Organocatalysis trials from paraformaldehyde:

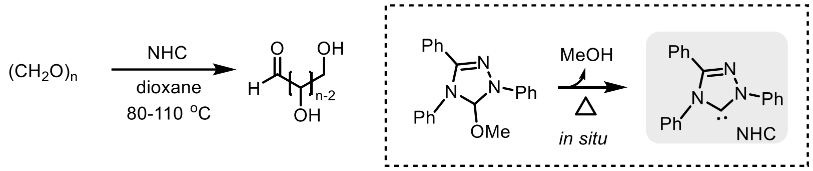

General conditions for C4 sugars: To a Schlenk flask was added alkoxy NHC precursor (3 mg, 0.01 mmol), paraformaldehyde (60 mg, 2 mmol) and solvent, such as dioxane (5 mL) under argon. The reaction flask was then sealed and placed in an 80 °C silicon oil bath with moderate stirring (200 rpm). Upon heating, paraformaldehyde rapidly dissolved and the solution became clear. The reaction was stopped at the desired time by removing from the heat. Rapid quenching was achieved by placing it in an ice bath.

General conditions for C5 sugars: To a Schlenk flask was added alkoxy NHC precursor (3 mg, 0.01 mmol), paraformaldehyde (60 mg, 2 mmol) and solvent, such as dioxane (5 mL) under argon. The reaction flask was then sealed and placed in a 90 °C silicon oil bath with moderate stirring (200 rpm). Upon heating, paraformaldehyde rapidly dissolved and the solution became clear. The reaction was stopped at the desired time by removing from the heat. Rapid quenching was achieved by placing it in an ice bath.

General conditions for C6 sugars: To a Schlenk flask was added alkoxy NHC precursor (9 mg, 0.03 mmol), paraformaldehyde (60 mg, 2 mmol) and solvent, such as dioxane (2.5 mL) under argon. The reaction flask was then sealed and placed in a 110 °C silicon oil bath with moderate stirring (200 rpm). Upon heating, paraformaldehyde rapidly dissolved and the solution became clear. The reaction was stopped at the desired time by removing from the heat. Rapid quenching was achieved by placing it in an ice bath.

NHC Product Analysis:
GC of Acetylated Product: The sugar product was dried by rotary evaporation. The crude product was dissolved in pyridine (3 mL), to which a sorbitol internal standard (36 mg, 0.2 mmol) was added, along with *N*-dimethylaminopyridine (10 mg, 0.08 mmol) as a catalyst, and a suitable amount of acetic anhydride (based on estimated -OH content. Typical: 1 mL). The reaction was stirred overnight and quenched by HCl aqueous solution (3 mL, 1 M). The acetylated sugars were extracted in ethyl acetate (5 mL) and washed five times by the addition of 3 mL 1 M HCl and excess water. The resulting solution was dried with Na_2SO_4 and directly injected into the GC (Shimadzu GC-2010 Plus, SH-PONA Capillary column, temperature 220°C).

GC-MS of Acetylated Product: The sugar product was acetylated as above and analyzed by an Agilent 7890B GC/5977A MSD system.

HPLC: Additional chromatographic analysis was performed with an Agilent 1260 Infinity II with MWD and RI detector. The column used was Aminex HPX-87H, 5 mM H_2SO_4, 0.5 mL/min flow rate, column temperature 55 °C, 5 μL loop, manual injection.

Bacterial Strain and Growth Conditions:

E. coli BW25113 cells were purchased from Horizon Discovery. The bacterial stock was stored at −80 °C. The NHC sugar solution was filtered to remove precipitates and sterilize. The appropriate mass of sugars was added to achieve a final concentration of 1% (w/v) in the M9 minimal medium (Table 1). For experimental use, the stock was thawed and inoculated into lysogeny broth (LB), followed by incubation at 37 °C overnight. After overnight incubation, the cells were inoculated to an OD_{600nm} of 0.08 in 250 mL baffled flask containing 50 mL M9 minimal medium supplemented with the specified sugars. The cells were grown aerobically at 37 °C with shaking at 200 rpm. Optical density (OD) at 600 nm was measured every 3 h using a UV-Vis spectrometer to assess

cell growth. The samples were collected after removing cells by centrifuging at 15000 rpm for 5 min in a table-top microcentrifuge to remove the cells.

Cobalt phthalocyanine for electrochemical conversion of carbon dioxide to methanol

CoPc/CNT Synthesis: 1.2 mg cobalt phthalocyanine (Alfa Aesar, 98%) was dissolved in 10 mL *N*,*N*-dimethylformamide (DMF) and sonicated thoroughly until clear. 30 mg of multiwall carbon nanotubes (XFNano, 10-20 nm O.D., 95%) were typically washed by calcination in air at 500°C for 1 hour, followed by stirring in 5 wt% HCl overnight, washing with deionized water until the pH was neutral, and dried via vacuum oven at 60 °C overnight. Then 30 mg of dried CNTs were vigorously sonicated with cooling in 30 mL of DMF until well-dispersed. 1.2 mg CoPc was dissolved in 10 mL DMF, assisted by sonication. The CoPc and CNT suspensions were mixed and sonicated together for one hour before stirring at 500 rpm overnight. The final product was washed by centrifugation three times with DMF and three times with ethanol before being dried in the vacuum oven or by air stream. For the twice-loaded catalyst, we repeated the synthesis, substituting for CNTs from the above protocol and neglecting to repeat the initial washing steps.

CoPc/CNT Characterization:
CV: Verification of the $Co^{II/I}$ redox wave was performed in an undivided cell. 0.1 mg of catalyst was drop-cast onto a Sigracet 39AA carbon paper (1 cm^2) and dried before placing in 0.1 M $NaClO_4$ in 18.2 MΩ H_2O. The reference electrode (3M KCl, Dri-Ref, World Precision Instruments) and counter electrode (Pt wire, 99.99%, Sigma Aldrich). Scans were performed at 50 mV/s and cycled until steady state behavior was observed.

X-ray Absorption Spectroscopy (XAS): XAS was collected in total fluorescence mode with a 100-element Ge monolith detector system at the Stanford Synchrotron Radiation Light Source (SSRL) 9-3 beamline. Experiments were performed in a custom PEEK electrochemical divided H-cell. For CoPc/CNT, loading was 1 mg cm^{-2}; electrolyte: 0.1 M $KHCO_3$. CoPc powder was drop-cast from an ethanol dispersion (50 μg cm^{-2}). A Biologic SP-300 potentiostat in floating configuration was used for electrochemical testing. Ar and CO_2 gases at 20 sccm were used for OCV and -0.6 V vs. RHE trials, respectively. Data normalization and alignment to a Co foil reference was performed in ATHENA.

ICP-OES: Inductively coupled plasma optical emission spectroscopy (ICP-OES) measurement was performed using PerkinElmer Optima 7000 DV.

X-ray photoelectron spectroscopy (XPS): XPS was performed using a Thermo Scientific K-Alpha, monochromatic aluminum K*a* with a spot size 400 μm and 50 eV pass energy at Molecular Foundry at Lawrence Berkeley National Laboratory. Peak fitting was performed using a Shirley background subtraction, and spectra were energy corrected to set adventitious carbon at 284.8 eV.

Scanning electron microscopy (SEM): Scanning electron microscopy characterization of the post-electrolysis CoPc/CNT electrodes was performed on the Zeiss Ultra-55 SEM at Molecular Foundry at Lawrence Berkeley National Laboratory.

Scanning transmission electron microscopy (STEM): High-resolution STEM imaging of the CoPc/CNT atom catalyst was performed on the double-aberration-corrected TEAM I microscope at the National Center for Electron Microscopy (NCEM) at Lawrence Berkeley National Laboratory. The instrument was operated at 300 keV with a beam convergence semi-angle of 30 mrad.

Energy dispersive X-ray spectroscopy (EDS): High-angle annular dark-field scanning transmission electron microscope (HAADF-STEM) and energy dispersive X-ray spectroscopy (EDS) mapping analysis were performed with Thermo Fisher Scientific TitanX 60-300 with an

accelerating voltage of 80 kV. STEM-EDS 140 eV energy resolution; windowless Si drift detectors, total solid angle 0.7 steradian; Au grid.

CoPc/CNT Electrochemistry:
Electrode Preparation: For typical ink preparation, 4 mg of CoPc/CNT catalyst was suspended in 2 mL of ethanol and 30 μL Nafion D520 ionomer and sonicated thoroughly. The desired mass was drop-cast onto carbon paper (AvCarb GDS2230) 2 x 2 cm and dried thoroughly by desiccation overnight before use.

Flow Cell: The schematic of the flow cell is shown in Fig. 3d. Briefly, gas and catholyte are passed in separate chambers around the gas diffusion electrode loaded with CoPc/CNT catalyst. The gas flow rate was controlled by a mass flow controller (OMEGA) and measured downstream of the cell by the soap-bubble method. The cell was operated by a Biologic Potentiostat SP200. The catholyte (0.5 M K_2HPO_4, Fisher Scientific ACS grade) and anolyte (1 M KOH, Fisher Scientific ACS grade) were separated by an anion exchange membrane (Fumasep FAA-3-PK-130) and individually recirculated (electrolyte volume: 30 mL). A commercial IrO_x counterelectrode supported on Ti fiber felt was used (Magneto). A 1 M KCl Ag/AgCl reference electrode (CH Instruments CHI111) was used for the three-electrode configuration. The iR compensation was post-corrected. RHE values are then calculated as follows:

$$E_{RHE} = E_{Ag/AgCl} + 0.23 + (0.0591 * pH) + iR$$

Membrane electrode assembly (MEA): The schematic of the membrane electrode assembly cell is shown in Fig. 5a. Briefly, gas and electrolyte are passed in separate sides around the gas diffusion electrode loaded with CoPc/CNT catalyst. The gas flow rate was controlled by a mass flow controller (OMEGA) and measured downstream of the cell by the soap-bubble method. The cell was operated by a Biologic Potentiostat SP200. Chambers (0.1 M $KHCO_3$, Sigma Aldrich, ACS reagent 99.7%) were separated by an anion exchange membrane (Sustainion X37-50 Grade RT) and individually recirculated (electrolyte volume: 20 mL). Methanol was measured from the recirculated electrolyte. A commercial IrO_x counterelectrode supported on Ti fiber felt was used (Magneto).

Product Analysis*:* The gaseous products of the flow or MEA cell (H_2, CO, CH_4) were analyzed by gas chromatography (Agilent Technologies, 7890B) using a flame ionization detector (FID) and a thermal conductivity detector (TCD). Gas products were collected directly by syringe at the outlet of the flow cell to obtain time resolved gas evolution data. Quantitation of products was performed vs. multi-point calibration for high concentration flow cell products. Liquid products were analyzed by ^{1}H NMR and quantified vs. an internal standard (DMSO).The liquid products were analyzed with a Bruker AV-600 or Bruker NEO-500. Dimethyl sulfoxide was used as the internal standard, and electrolyte solution was added to D_2O in a 9:1 ratio. Solvent suppression by excitation sculpting was used to suppress the water peak.

Formaldehyde Assay*:* The colorimetric Nash assay was performed on the CoPc/CNT liquid product stream. To prepare the assay reagent, 0.75 g ammonium acetate was dissolved in 5 mL H_2O, with 10 μL acetylacetone. For each sample, 150 μL of sample and 150 μL reagent were mixed in a well plate and developed in the dark until absorbance reading was stable. 412 nm absorbance was measured on a Biotek Synergy LX Multi-Mode plate reader. A calibration series accounting for electrolyte matrix effects ranging from 10-600 μM was prepared for quantitative analysis. Samples were diluted to within this linear range as necessary.

Photocatalytic conversion of methanol to formaldehyde by zinc indium sulfide

Colloidal synthesis of ZIS NCs:
$ZnCl_2$ (138 mg, 1 mmol, 98%+), $InCl_3$ (147 mg, 0.66 mmol, 99.99%), trioctylphosphine oxide (2.3 g, TOPO, 99%) were added into a three-neck flask in a glovebox. Then the flask was moved out from the glovebox and 10 mL of oleylamine (OAm, 70%, technical grade) was quickly added. The

resulting mixture was heated at 120 °C under vacuum for 60 min, after which the reaction solution was heated further to 220 °C (internal temperature). Hot injection of sulfur (57.6 mg, 1.8 mmol, dissolved in 2 mL OAm) resulted in an immediate color change from light yellow to dark red, which then gradually changed to orange yellow upon heating at 220 °C for 1 h. Then mixture was then cooled down to room temperature and 30 mL of ethanol was added. The precipitate was collected by centrifugation at 12000 rpm for 5 min and redissolved in toluene (10 mL). The solution was further centrifuged at 12000 rpm for 10 min and the supernatant was collected. The composition of the resulted ZIS NCs was determined by ICP-OES as $Zn_{1.65\pm0.1}In_2S_x$ after sonicating in aqua regia overnight. Size screening was further carried out using ethanol as the antisolvent for TEM images.

Ligand exchange: The hydrophobic native surface-capping ligands of the as-synthesized ZIS NCs were subsequently replaced with 3-mercaptoproponate (3-MPA) ligands following a previously reported procedure. Briefly, an aliquot (10 mg, ~700 μL in toluene) of the native ligand capped ZIS NCs was added to a solution of 1.25 mmol 3-MPA and 300 mg tetramethylammonium hydroxide in 20 mL methanol, the resulting suspension was stirred at room temperature for 20 min. 20 mL of toluene was then added and the resulting mixture was centrifuged at 12000 rpm for 5 min. The solid was collected and washed three times with 3 mL MeOH and then redispersed in 5 mL water.

ZIS Characterization

Powder X-ray Diffraction (PXRD): PXRD data were collected using a Rigaku Miniflex 6G Benchtop Powder XRD with a Cu Kα radiation source in ambient conditions.

UV-vis Absorption Spectroscopy (UV-vis): The absorption spectrum was acquired using a UV-vis spectrometer (UV-2600, Shimadzu). The sample can be either a liquid sample (0.4 mg/mL in water) or a thin film. For thin film preparation, initially, 10 μL of ZIS NCs dispersion (0.4 mg/mL in water) was deposited onto a quartz slide, and upon heating to 70 °C, water was evaporated to form a thin film of ZIS NCs. A background scan was conducted solely for the quartz slides. Subsequently, the Tauc plot was derived from the absorption spectrum, plotting $(\alpha h\nu)^2$ against $h\nu$.

X-ray photoelectron spectroscopy: X-ray photoelectron spectroscopy (XPS) spectra were recorded using Thermo Fisher Scientific K-Alpha Plus X-ray Photoelectron Spectroscope with a monochromatic Al-Kα source at Molecular Foundry at Lawrence Berkeley National Laboratory (LBL). Shirley background was used for background subtraction. The C1s peak was used to calibrate the binding energy.

Ultraviolet photoelectron spectroscopy (UPS): XPS and UPS were measured with a PHI Versa Probe IV multi-technique instrument at Stanford Nano Shared Facilities (SNSF, RRID:SCR_023230). For XPS, the measurement was carried out using an Al Kα source (Photon energy 1486.6 eV) and 224 eV pass energy, with electron and ion neutralization. UPS was performed using He I as the photon source operating at 1000 V and 100 mA. The sample was prepared by dropping a 10 μL aliquot of ZIS NCs dispersion (0.4 mg/mL in water) onto an indium tin oxide (ITO) substrate, and upon heating to 70 °C, water was evaporated to form a thin film of ZIS NCs. The data was collected with 10 V bias. The work function and valence band maximum (VBM) of ZIS nanocrystals were measured by UPS to be –3.78 eV and -7.01 eV versus vacuum, respectively. The work function was calculated by subtracting the He I radiation energy of 21.2 eV from the high-binding energy cutoff at 17.42 eV: (7.42 + 10) eV – 21.2 eV = –3.78 eV versus vacuum. VBM: -3.78-(-6.77+10) = -7.01 eV versus vacuum, which is -2.57 V vs SHE.

Electron Paramagnetic Resonance (EPR): Continuous-wave (CW) X-band electron paramagnetic resonance (EPR) measurements were performed at room temperature using a Bruker Elexys E580 spectrometer equipped with a ER4119HS High-Q CW resonator operating at a frequency of 9.83 GHz. All spectra were recorded with a modulation frequency of 100 Hz and modulation amplitude of 1 G. The microwave power used for all spectra was 0.5972 mW and was spot-checked by attenuating the power to determine that the signal response was linear. While these

parameters may lead to minor broadening of the narrow lines for the organic radicals in the present study, they were necessary to improve the sensitivity to allow for the detection of the low concentration photoradical products.

To prepare the samples, specific amounts of $NiCl_2·6H_2O$ stock solution (0 wt% and 3 wt% Ni) were measured and added to the ZIS NCs dispersion. Additionally, 100 μL of fresh 5,5-dimethyl-1-pyrroline-N-oxide (DMPO) solution (0.1 mg/mL) was added to the mixture. The ZIS NCs dispersion consisted of 2 mg in 4 mL MeOH and 1 mL H_2O.
After thorough mixing, 40 μL of the resulting mixture was transferred into an EPR tube with a 2 mm diameter. The EPR tube was placed in the microwave cavity of the EPR spectrometer. Spectra were recorded under two conditions: either in the dark, or under a 365 nm LED with data collected after 4 minutes of irradiation at room temperature.

Flat-band Measurements (Mott-Schottky Plot): The photoelectrochemical measurements utilized a conventional three-electrode configuration. Initially, an aliquot of ZIS NCs dispersion (0.4 mg/mL in water) was deposited onto an indium tin oxide (ITO) substrate, forming a thin film of ZIS NCs (0.6 cm × 0.8 cm) upon heating to 70 °C to facilitate water removal. This ZIS NCs layer functioned as the working electrode. An Ag/AgCl electrode with a potential of 0.21 V versus the Standard Hydrogen Electrode (SHE) was utilized as the reference electrode, while a platinum wire served as the counter electrode. The electrolyte solution employed was 0.5 M Na_2SO_4 for Mott-Schottky analysis. Flat band measurement was conducted under dark conditions with the potential and frequency ranging from -1.0 to 1.2 V (vs. Ag/AgCl, pH = 7) and from 500 to 1500 Hz, respectively.

Transmission electron microscopy: The low-magnification images were taken on the Hitachi H-7650 microscope. High resolution TEM images were acquired with the TEAM0.5 microscope at 80 kV, located at the National Center for Electron Microscopy. Focal series data were acquired and processed with minor changes. The focal series was recorded from −20 to 0 nm defocus with a 2 nm step to avoid sample drift, and the beam was not monochromated. ZIS NCs dispersion (25 μg/mL) were drop-cast onto ultrathin carbon-coated Cu or Au TEM grids for imaging.

Energy dispersive X-ray spectroscopy (EDS): High-angle annular dark-field scanning transmission electron microscope (HAADF-STEM) and energy dispersive X-ray spectroscopy (EDS) mapping analysis were performed with Thermo Fisher Scientific TitanX 60-300 with an accelerating voltage of 80 kV. STEM-EDS 140 eV energy resolution; windowless Si drift detectors, total solid angle 0.7 steradian; Au grid.

ICP-OES: Inductively coupled plasma optical emission spectroscopy (ICP-OES) measurement was performed using PerkinElmer Optima 7000 DV. The molar ratio of Zn to In was determined to be 1.65±0.5 : 2 for different batches.

Photocatalytic methanol dehydrogenation:
The dispersion of ZIS NCs (2 mg/mL in water) was sonicated for 30-60 seconds to ensure uniform dispersion. Then ZIS NCs (4 mg, dispersed in 2 mL water), $NiCl_2·6H_2O$ (0.33 mg, 2 wt% Ni, from a 160 μL stock solution in water), and methanol (4 mL) were added into a 30 mL quartz tube (with a diameter of 1.5 cm). The tube was sealed with an adaptor equipped with a three-way valve and purged with argon. The reaction mixture was then exposed to light from a 300 W Xe lamp (with a light intensity close to 120 mW/cm^2) for a specified duration, while a fan maintained the reaction at room temperature. The gas generated was analyzed using GC chromatography, while the organic product was measured by proton ^{1}H NMR.

Scale up reaction:
A dispersion of ZIS NCs (80 mg in 40 mL H_2O), $NiCl_2·6H_2O$ (1.6 mg Ni, 2 wt%, in 3.2 mL stock solution in H_2O), and methanol (80 mL) were added to a 350 mL quartz tube (5 cm diameter). The tube was sealed with an adaptor equipped with a three-way valve, purged with argon, and connected to an inverted gas cylinder refilled with water (eudiometer). The reaction mixture was exposed to 370 nm light from a Kessil lamp at an intensity of 30-40 mW/cm^2 (variation of the

intensity due to the bigger diameter of the tube) for 72 hours, with a fan maintaining room temperature throughout the reaction. The collected gas was analyzed by GC, and the crude reaction mixture was measured by NMR with ethyl acetate as an internal standard. The mixture was then transferred to a 250 mL round-bottom flask for distillation, and the fraction boiling between 110 °C and 140 °C was collected. After removing water and methanol by air blowing, paraformaldehyde was obtained as a white solid. Its polymerization degree was further determined by NMR (DMSO-d_6) through the analysis of remaining end-OH protons.

Apparent quantum efficiency (AQE) test: The AQE (φ) calculation follows φ = nR/I, where n, R and I are the number of involved electrons, the hydrogen evolution rate and the amount of incident photons, respectively. The irradiation surface area was 8 cm^2 and the incident light intensity was measured to be 30 mW/cm^2. The experiment was carried out under 370 nm light irradiation for 3600 s. The generated HCHO was 0.235 mmol.

$\Phi = nR/I \times 100 = nR/(E\lambda/hc\times S) \times 100 = 17.5\%$
where E is the energy of photons, λ is wavelength, h is Plank's constant, c is speed of light, and S is the irradiated area.

Product Analysis
Nuclear magnetic resonance (NMR): All ^1H NMR, ^{13}C NMR spectra associated with ZIS experiments were recorded on a Bruker AVANCE III HD 500MHz NMR spectrometer and reported in ppm (δ). Chemical shifts were referenced to the residual solvent peaks.
NMR sample preparation: 0.5 mmol of ethyl acetate (49.1 μL) was added into the reaction mixture, and 8 drops of the resulting solution was transferred into the NMR tube followed by adding 0.6 mL DMSO-d_6 (NMR measuring parameters: d1 = 4 s, NS = 32; auto linear correction was applied for the integration).

Gas chromatography (GC): Gas products were quantified by a gas chromatograph (SRI) equipped with a thermal conductivity detector and a flame ionization detector. Quantification of the gas products was based on the conversion factor, which was calibrated using standard samples.

Discussion S1. Solar to Sugar Efficiency

To draw reasonable comparison of the theoretical efficiency of an abiotic 3-step process such as the one detailed in this work with the efficiency of conventional agricultural crops, we performed an analysis similar to those described in Blankenship and Cai *et al.* as well as our previous work, based on an integrated photovoltaic-driven electrolysis and photocatalytic process (1–3). This is predicated on the idea that one may want to effectively utilize total energy irradiating a specific land area by storing it as chemical energy. When one considers the total efficiency of solar-driven CO_2 – to – X, the two may be more directly compared as a starting point for considering future research targets. Here, X = harvestable *biomass* for crop (not the total extracted edible sugars, which is lower) or *total* NHC sugar for the abiotic process.

Defining the energy efficiency of a process is possibly contentious. We take a moment to acknowledge that there are several other ways of assessing the system efficiency. However, to present our estimate, we opted to use a simple definition related to that of the hypothetical solar to stored-chemical-energy calculations used in Blankenship et al. One can envision, as suggested in the main text, a process in which electrocatalysis is powered by a photovoltaic (PV) source. The individual components of the total efficiency calculation are each an energy conversion efficiency value, described below. We do not incorporate estimated energy penalties from real process inefficiencies, nor do we account for separation costs as indicated in the process integration discussion.

For our abiotic process, we compute the total system efficiency (solar to sugars efficiency, SSE):

$$SSE = (STP \times EE_{MeOH}) \times LTC \times TE_{formoin} \times \gamma_{formoin}$$

Each component is described below:

1) To account for the conversion of solar energy to electrical energy to power the electrochemical process,

STP = solar to power conversion of the PV

STP is assumed to be 20%

2) $\mathbf{EE_{MeOH}}$ is intrinsically described by two factors, namely the overpotential of the catalyst (and cell configuration) and the faradaic efficiency to a particular product:

$$EE_{MeOH} = FE_{MeOH} \times \frac{E^{o}_{CO2\rightarrow MeOH}}{E_{cell}}$$

$\mathbf{E_{cell}}$ = Actual full-cell operating potential, made up of all kinetic overpotentials (e.g. ohmic losses through solution and ion exchange membrane, anodic and cathodic overpotentials). The overpotential associated with the CO_2 cathodic reduction to methanol varies in principle by system; in practice, the CoPc/CNT system has remarkably consistent cathodic overpotential across reports and can be treated as a fixed value, ~ 1 V.

$\mathbf{FE_{MeOH}}$ **=** Faradaic (current) efficiency associated with the CO_2 cathodic reduction to methanol (in principle, variable and to be optimized; in this work, 25%)

$\mathbf{E^{o}_{MeOH}}$ **=** Thermodynamic potential for a cell with CO_2 reduction to methanol at the cathode and oxygen evolution reaction (OER) at the anode. (1.21 V)

In this work, $\mathbf{FE_{MeOH}}$ is experimentally determined to be approximately **27%**. In this work, the flow cell is not optimized to minimize overpotential at the anode, and as such we draw optimistic estimates from mature CO_2RR technologies. In the best configurations, a minimum anodic + ohmic overpotential may be approximately 500 mV. The additional cathodic overpotential of approximately 1 V in this work leads to the hypothetical E_{cell} = 1.21+1.5 = 2.71 V, close to that obtained for state-of-the-art CO_2-to-CO electrolyzers (4). This cell potential will be used in the summary table for "future target" calculations.

Therefore, our estimated flow cell EE_{MeOH} (drawn from achieved efficiency and estimated achievable improvements) is 11.2%, an overestimate of the true value, but which is within reason for lab-scale implementation.

The real value, determined from the full cell voltage (3.26 V) and faradaic efficiency (20%) of the MEA, would be 7.4%. This value is used in the summary table as "this work, two-step."

For an assessment of the future targets of CoPc/CNT to methanol, we consider the pace of incremental advancements to the CoPc/CNT system by Wang, Robert, Ye and others. Currently, CORR is more efficient, with methanol efficiency ~ 66%. With significant improvements of the system to achieve similar literature benchmarks for CO_2RR, FE_{MeOH} = 66% and the theoretical lowest full cell voltage of 2.71 V, would yield an EE_{MeOH}= 29.5% ("future target, two-step"). The highest reported FE_{MeOH} for CO_2RR using strained CoPc on single wall-nanotubes as a support was 53%, indicating a promising possibility for future improvements. Tuning selectivity to achieve similar yield to formaldehyde is used in the summary table as "future target, one-step."

3) **LTC =** light-to-chemicals, here defined as the useful energy stored as product per total incident energy on the photocatalytic system. This is analogous to the well-known Solar-to-Hydrogen or Solar-to-Chemicals concepts in photocatalysis literature. The equation relates useful energy achieved by the transformation divided by incident light energy used:

$$LTC = \frac{\Delta G^o \times n}{PAT}$$

ΔG = Free energy change of reaction (J/mol)
n = moles of product produced
P = power density of incident light, measured for full spectrum ($J/s/cm^2$)
A = area of irradiation (cm^2)
T = irradiation time (s)

LTC as used here represents the energy conversion efficiency for a photocatalyst as chemical energy stored (out) per incident light energy (in). It is a more conservative/pessimistic value than a quantity such as apparent quantum efficiency (AQE), which is commonly reported for photocatalysts and is also described in this work. LTC *de facto* includes losses due to thermalization, charge recombination, and other side reactions and inefficiencies. It is also a more tangible and realistic value, as it aims to represent the total energy cost of producing the chemical. The challenging factor is the choice of the energy basis in the numerator. Drawing from Blankenship et. al, and to present the most *conservative* estimate, we use the free energy of formation of formaldehyde from methanol (CH_3OH (l) → CH_2O (g) + H_2 (g)) as opposed to the heat of combustion of the formaldehyde product.

LTC thus is quite similar to AQE but compares energies instead of numerical accounting for photons in and photons out. Thus, using a higher energy light source which may be better suited for the photocatalyst's band gap, such as a UV lamp, will incur a penalty in the LTC calculation that is not described in reporting AQE.

Then for our conditions that produced the AQE of 17.5%,

$$LTC = \frac{63.74\ \frac{kJ}{mol} \times 0.235\ mmol}{0.03\ \frac{J}{scm^2} \times\ 8\ cm^2\ \times 3600\ s} = \frac{14.9\ J}{864\ J} = 1.7\%$$

Which doubled at 70 °C to yield, not accounting for the energy required to heat the reaction, 3.4% LTC (from conditions leading to AQE of 35%). The low energy conversion efficiency is common in high light flux photoreactors where not all photons are absorbed or transformed into useful chemical energy.

4) **$TE_{formoin}$** = thermal efficiency of the organocatalytic step, assumed to be 80%, on par with low-T thermal reactors

$\gamma_{formoin}$ = carbon conversion efficiency of the NHC formoin to monosaccharides, ~ 80% (experimental)

With the fixed values from above, the SSE reduces to:

$$0.2\ \times\ EE_{MeOH}\ \times LTC\ \times 0.64$$

Immediately, the peak efficiency is limited by factors such as solar to power conversion for electrocatalysis, the carbon yield of the formoin reaction, and the energy inefficiency of the thermal organocatalytic reaction. This product is estimated to be 12.8%, which is coincidentally close to the theoretical limit to photosynthetic efficiency (~12%), far beyond what actual crops accomplish, and similarly likely far beyond what a real photovoltaic-powered abiotic process might achieve with current technology.

In Fig. 1b we indicate a range of a favorable "real" crop SSE of 0.1-1% as a target. Noteworthy is that "real" solar-to-biomass conversions of agricultural crops, annualized, is often below 1%, and thus though the SSE is an optimistic value that does not describe real process inefficiencies, it shows that total process is within range of land allocation utility with extensive room for improvement.

The following table summarizes the various scenarios of electro/photocatalytic efficiencies for the current implementation and the optimistic cases:

Scenario	STP (%)	EE_{MeOH} (%)	LTC (%)	Formoin# (%)	SSE (%)
This work, two-step	20	7.4	1.7	64	0.01
This work, one step*	20	2 (as EE_{HCHO})	-	64	0.26
Optimistic future target, two-step**	20	29.5	3.4	64	0.1
Future target, one-step***	20	32 (as EE_{HCHO})	-	64	4

* Here, FE_{HCHO} = 5%, and thus the energy efficiency approaches 2% for the electrochemical step. This, multiplied by STP (20%) and Formoin (64%) yields the SSE shown.

**Where CO_2 – to – methanol efficiency approaches the highest reported CO – to -methanol faradaic efficiency (66%) and full cell voltage reduces to the lowest estimated viable for this system, 2.71 V. Where PMOR LTC efficiency is calculated from the improved conditions at 70 °C.

*** Where we envision that, on a system similar to CoPc/CNT, *CO_2 – to – formaldehyde* efficiency approaches the highest reported *CO – to -methanol* faradaic efficiency (66%) and full cell voltage reduces to the lowest estimated viable for this aqueous system, 2.71 V. Then the one-step electrochemical energy efficiency to formaldehyde is approximately 30%, and direct integration to the NHC formoin reaction (again neglecting isolation steps) affords a SSE exceeding that of conventional agricultural plants.

Formoin (%) is the product of $TE_{formoin} \times \gamma_{formoin}$

We emphasize that even with low Faradaic efficiency, the energy gains by avoiding a second photo- (or electro-) chemical step can place the abiotic sugars process quite close to the efficiency range that crops achieve annually. In the future target optimistic one-step scenario, where the CO_2 reduction performance of a catalyst such as CoPc is improved to be on par with that of the efficiency of CoPc/CNT utilizing CO as the reactant to make methanol, and with minor improvements to full cell potential in an electrolyzer such as the MEA, the energy efficiency would approach or exceed parity with biomass. In our system, we see greater calculated efficiency in even the low-yield formaldehyde case. Isolating the anhydrous formaldehyde will require great improvement to the production rate but may be within reach of the quickly advancing CO_2RR community. Thus, one can state that the photocatalytic oxidation step is a current necessity for high-yield formaldehyde production but is energetically costly and must be greatly improved or eliminated in future iterations.

For comparison, mature thermocatalytic technologies may be difficult to translate into solar or renewably powered processes due to their high temperature and pressure requirements. However, the individual steps have been reported to have high energy conversion efficiencies, and industry is taking steps toward utilizing green hydrogen and low-carbon energy technologies. Therefore, incorporating formaldehyde generated in this way may enable highly efficient sugar synthesis from carbon dioxide with the advent of the new NHC formoin chemistry:

Scenario	EE_{MeOH} (%)(3)	EE_{HCHO} (%)(5)	Formoin (%)	EE
Thermocatalytic	68	66	64	28.7 (neglects solar power conversion)

In Fig. 1b, we de-emphasize certain important catalytic factors in the selection of a two-parameter description, as each parameter itself is made up of several experimentally relevant factors. Here, we have elaborated. Some of these, such as cell ohmic loss due to membrane and solution resistance, are fairly readily addressed (in this work, by employing a MEA instead of a flow cell configuration). Some, such as the usable photon energies, are best targeted by materials engineering and will be relevant to a PMOR process using the solar spectrum. Some, such as the cathodic overpotential of CoPc/CNT, may be intrinsic to the selected system (based on molecular activation governed by intrinsic electronic properties of the catalyst) and must also be improved by further exploration of suitable catalysts.

The productivity comparison was calculated by considering the bottleneck (slowest) step in the total abiotic conversion process. Total conversion rate of carbon is considered per unit catalyst (in the case of biocatalytic strategies, μmol/mg enzyme; for abiotic sugars, cm^{-2} electrode or mg^{-1} photocatalyst). A representative value of an enzymatic process along with an estimate for maize CO_2 fixation rate is drawn from Cai et al.(3). The abiotic process is here rate-limited by the electroreduction of CO_2 to methanol. From the partial current density described in the main text, rate is easily seen:

$$\mu mol\ conversion\ CO_2\ cm^{-2}\ h^{-1} = \frac{j\left(\frac{mA}{cm^2}\right) \times 3600\left(\frac{s}{h}\right)}{1000\left(\frac{mA}{A}\right) \times 6\left(\frac{mol\ e^-}{mol\ MeOH}\right) \times 96485\left(\frac{C}{mol\ e^-}\right) \times \left(\frac{1\ mol}{1000000\ \mu mol}\right)}$$

Which amounts to an approximately two orders of magnitude increase over biocatalytic approaches.

Although the foregoing analysis is not comprehensive, it is included in the sense that when allocating land for solar-to-fuels or agriculture, it may be essential to consider theoretical efficiencies. If a process is uneconomical or marginally competitive on a thermodynamic basis, inevitable efficiency losses of a real implementation will inhibit industrialization, as has been the case for many promised sustainable technologies. Though the current implementation of abiotic sugars (<0.1%) does not surpass natural photosynthetic efficiency, it is clear that reduction of catalytic process steps (CO_2 → CH_2O) and incremental improvements to formoin efficiency and selectivity will enhance the energy-based appeal of this strategy, which already has noted benefits of higher rate and fewer intrinsic externalities shown in Fig. 1.

Discussion S2. NHC Formoin Sugar Quantification

The quantification of carbohydrate products from the NHC catalysis is achieved by the use of response factors of carbohydrates of a certain type. That is, there are different FID response factors, e.g., for (aldo/keto)tetrose, pentose, and hexose products, but of the (e.g.) group of aldopentoses, the response factors are similar.

We first established response factors for the calibration standards (triose, tetrose, pentoses, hexoses) vs. an internal standard, sorbitol, which is added to each sample before acetylation (0.2 mmol). We find that each class of carbohydrate may have different FID responses but that agreement within each is adequate (aldopentoses have similar responses at the concentration range used for screening reactions).

We directly find mmol of the sugar by utilizing this response factor, and the sorbitol internal standard ensures consistency, as is typical for GC quantification. Then multiplying by number of carbons per molecule yields mmol of carbon attributed to each sugar, which when divided by mmol of formaldehyde (by weight of paraformaldehyde loaded) gives us percent yield. Selectivity, where reported, is normalized to 100% carbon yield. Due to variations in response factors, especially for the triose and tetroses, there is a systematic error for direct quantification leading to slight overestimation of yield.

	C3	C4	C5	C6		Sorbitol
**Area (Counts)**	**0.00**	**134953.00**	**1329212.00**	**120004.00**		**1672859.00**
**RF**	**0.09**	**0.08**	**0.67**	**0.77**		
**Amount (mmol)**	**0.00**	**0.19**	**0.24**	**0.02**		**0.20**
**Yield (% Carbon conversion)**	**0.0**	**38.6**	**59.2**	**5.6**		
**Total Carbon**	**2.1**				**103.4**	**%**
**Selectivity**	0.0	37.3	57.3	5.4		

Discussion S3. NHC Mechanistic Comparison With Formose

An interesting point arises from comparison with the classic formose reaction, broadly suggested as a concept for implementation of CO_2 upgrading. The pre-generated NHC allows for selective utilization of the C_1 species as a substrate, enabling controlled C_1 addition and limiting the likelihood of unwanted side products. Unlike the formose reaction, the condensation decreases the generation of branched sugars. Lastly, the exclusion of base prevents loss of aldehydes through Cannizzaro reactions, improving carbon yield to sugars.

Discussion S4. Electrochemical Calculations

Faradaic efficiency is calculated as the number of electrons that are converted to a desired product divided by the total number of electrons passed during electrolysis:

$$\frac{n \times z \times F}{Q_{tot}} \times 100\%$$

Where n is moles of product generated, z is electrons required per equivalent of i, F is Faraday's constant (96485 C/mol electrons^{-1}).

The partial current density relates directly to the rate of production of i:

$$j_i = FE_i \times j_{tot}$$

Discussion S5. CoPc Optimization Considerations

Although significant challenges remain in standardizing, democratizing and improving the performance efficiency of CoPc/CNTs for electrocatalytic production of methanol from carbon

dioxide, two major considerations were kept in mind for this report: 1) catalyst synthesis and 2) cell configuration/kinetic effects.

Regarding 1), efforts were made to improve the dispersion of CNTs and CoPc to improve the molecular loading. Initial efforts were stymied by variation between CNT manufacturers and batch-to-batch sonication differences. After screening a variety of CNTs for best DMF dispersion, we were able to achieve adequate catalytic performance with the most vigorous combination of tip and bath sonication available to us. To the best of our knowledge, the characterization of the resulting catalyst matched that of other literature reports, within reasonable scrutiny. Therefore, consideration 2) was explored further based on a simple expectation of the cascade reduction mechanism posited for the system. In a 6 e^- reduction, the pathway proceeds through a CO intermediate (2e^- reduction) before potentially desorbing as free CO or being retained for further reduction to formaldehyde (total 4 e^-), which may also desorb but is more readily reduced further to methanol (total 6 e^-). This “cascade” scheme implies a major role for recapturing the lost CO species, which is most directly achieved in the tortuous diffusion path of the CNT electrode by simply increasing total catalyst loading, increasing the diffusion path thickness and allowing for higher j_{MeOH} to be achieved.

Discussion S6. ZIS Quantification Procedure

1. HCHO converts into methoxymethanol in water and MeOH solution spontaneously, which can be detected by NMR (peaks at 4.5 ppm and 6.2 ppm). One can also use HPLC equipped with RI detector (less sensitive) or the Nash Colorimetric Assay to determine its concentration.
2. To determine the average light intensity on the photoreactor vessel:

$$\frac{88\ \text{mW/cm}^2 * 2.5\ \text{cm}^2 + 120\ \text{mW/cm}^2 * 2.25\ \text{cm}^2 + 220\ \text{mW/cm}^2 * 0.5\ \text{cm}^2}{5.25\ \text{cm}^2} = 114\ \text{mW/cm}^2$$

3. HCOOH, EG and CH_4 are the major side products, which usually count as less than 2% in total.

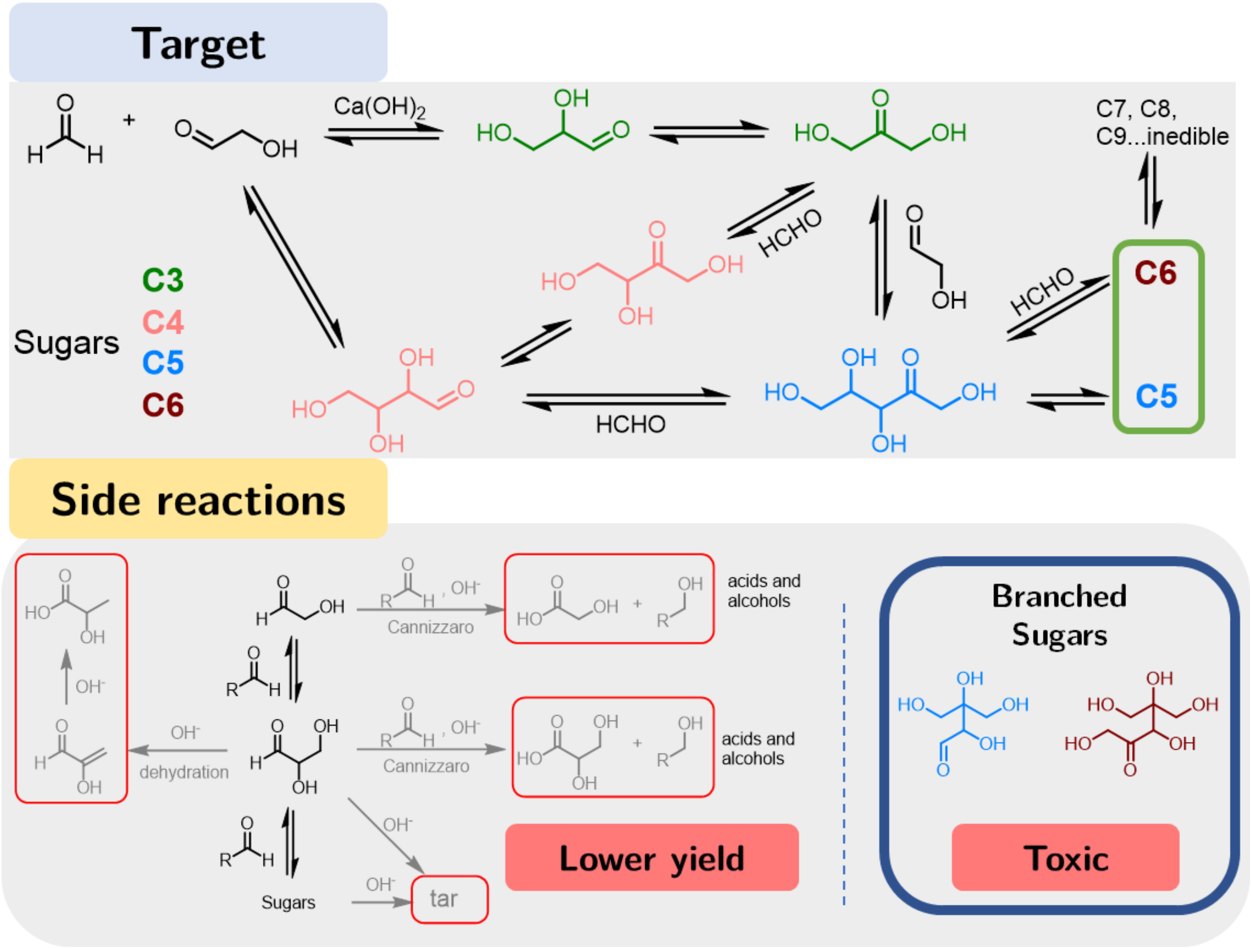

Fig. S1.
Formose reaction mechanism for comparison to formoin. The alkaline environment promotes undesirable side reactions such as dehydration, Cannizzaro disproportionation, and coupling to produce branched sugars.

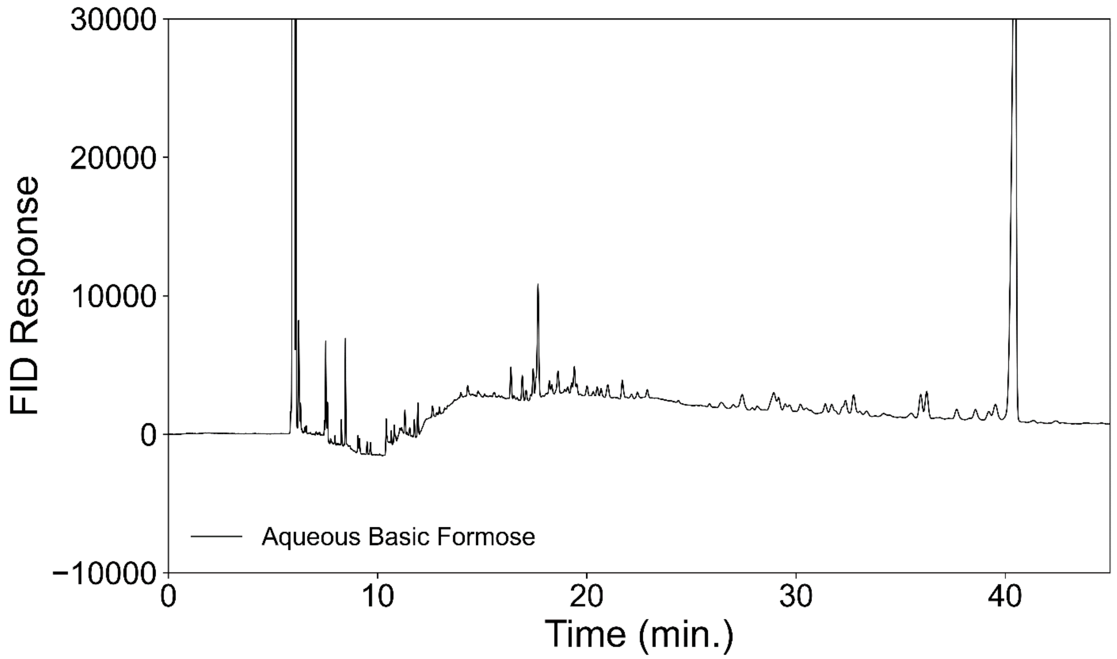

Fig. S2.
Gas chromatogram of the optimized classic formose reaction (acetylated). A broad range of species with relatively low yield is observed (see Table S1).

Table S1.
Select conditions screening for carbohydrate synthesis from formaldehyde, including comparisons with abiotic formose reactions.

Entry	Cat.	T (°C)	Time (h)	Selectivity (%) C3	C4	C5	C6	Yield (%)
1	**NHC-1**	80 °C	3	0	84	16	0	#N/A
2	**NHC-1**	90 °C	3	0	37	57	5	103
3[a,b]	**NHC-1**	100 °C	3	0	23	47	30	67
4[c]	$Ca(OH)_2$	80 °C	0.5	25	13	33	28	36
5[d]	$Ca(OH)_2$	60 °C	0.5	43	24	22	12	44
6[e]	$Sr(OH)_2$	80 °C	1	6	27	46	20	33
7[f]	$Sr(OH)_2$	80 °C	1	10	30	16	45	17
Solvent screening								
8[g]	**NHC-1**	120 °C	3	36	62	2	0	52
9[h]	**NHC-1**	100 °C	3	0	27	68	4	9
10[i]	**NHC-1**	100 °C	3	0	67	30	3	105
11[j]	**NHC-1**	120 °C	3	0	62	38	0	24
12[k]	**NHC-1**	120 °C	5	0	37	45	18	79
13[l]	**NHC-1**	120 °C	3	0	43	43	13	109
Catalyst screening								
14	**NHC-2**	100 °C	3	22	67	2.5	0	92
15	**NHC-2**	100 °C	2	0	85	14	0	105
16	**NHC-3**	100 °C	3	7	82	11	0	100
17[l]	**NHC-3**	120 °C	3	14	52	25	10	73
18	**NHC-4**	100 °C	2	100	0	0	0	10
19[l]	**NHC-4**	100 °C	2	100	0	0	0	53
20	**NHC-5**	100 °C	2	0	0	0	0	0
21	**NHC-6**	100 °C	2	25	73	3	0	92

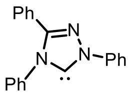

NHC-1

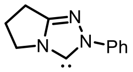

NHC-2

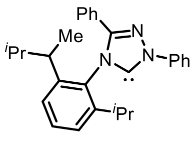

NHC-3

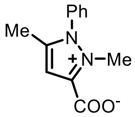

NHC-4

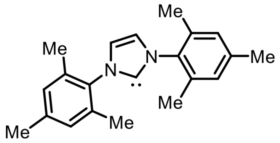

NHC5

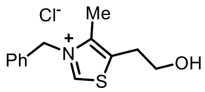

NHC-6

Standard conditions: HCHO (60 mg, 2 mmol), **NHC-1** (3 mg, 0.01 mmol), 80 °C, dioxane (5 mL), yield was estimated on carbon basis. a. dioxane (2.5 mL) b. **NHC-1** (9 mg, 0.03 mmol). c. Classic Formose conditions: HCHO (120 mg, 4 mmol), 5 mL H_2O, pH 11 by $Ca(OH)_2$, 80 °C, 100 *m*M glycolaldehyde. d. Air company: 0.87 M HCHO (120 mg, 4 mmol), 10% methanol v/v in 4.6 mL water, 60 °C, 0.1 g $Ca(OH)_2$, 0.1 g L-proline. e. MORose Analogue: 0.4 M HCHO in 4 mL MeOH/0.5 mL H_2O, 80 °C, 100 mM glycolaldehyde, 0.1 M $NaClO_4$, 40 mM $Sr(OH)_2$. f. Methanolic Formose: 0.4 M HCHO in 4 mL MeOH/0.5 mL H_2O, 80 °C, 100 mM glycolaldehyde, 40 mM $Sr(OH)_2$. g. DMF (5 mL) as solvent. h. Methanol (5 mL) as solvent. i. Dioxane (5 mL) and methanol (0.1 mL) as solvent. j. Dibutyl ether, 5 mL. k. Diglyme (bis(2-methoxyethyl) ether), 5 mL. l. 2-methoxyethanol, 5 mL. m. triethylamine (TEA) base addition for catalyst activation, 50 µL.

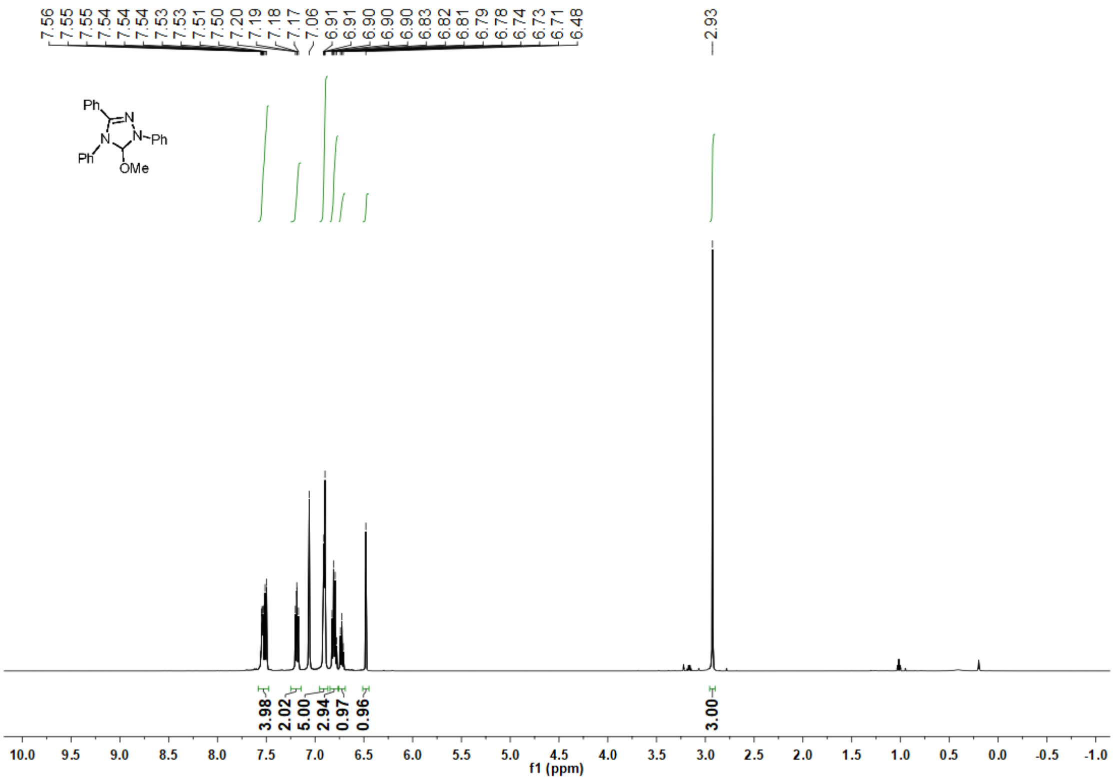

Fig. S3.
^{1}H NMR (500 MHz, C_6D_6) spectrum of **NHC-1**.

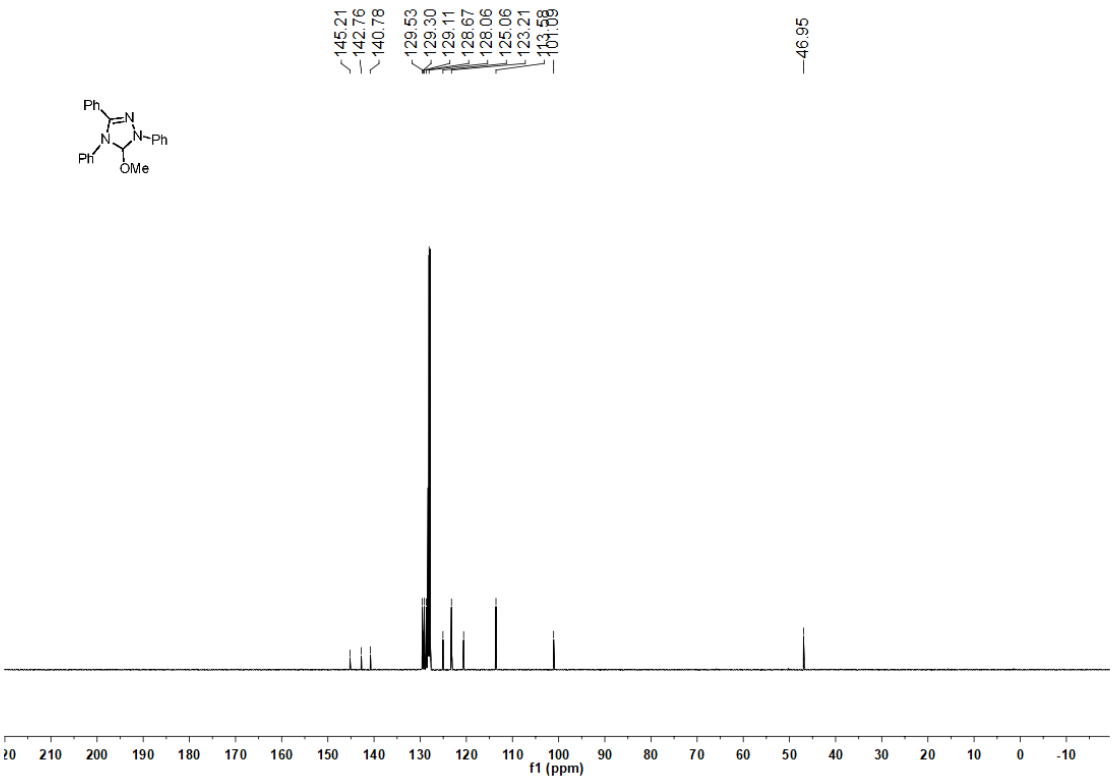

Fig. S4.
^{13}C NMR (126 MHz, C_6D_6) spectrum of **NHC-1**.

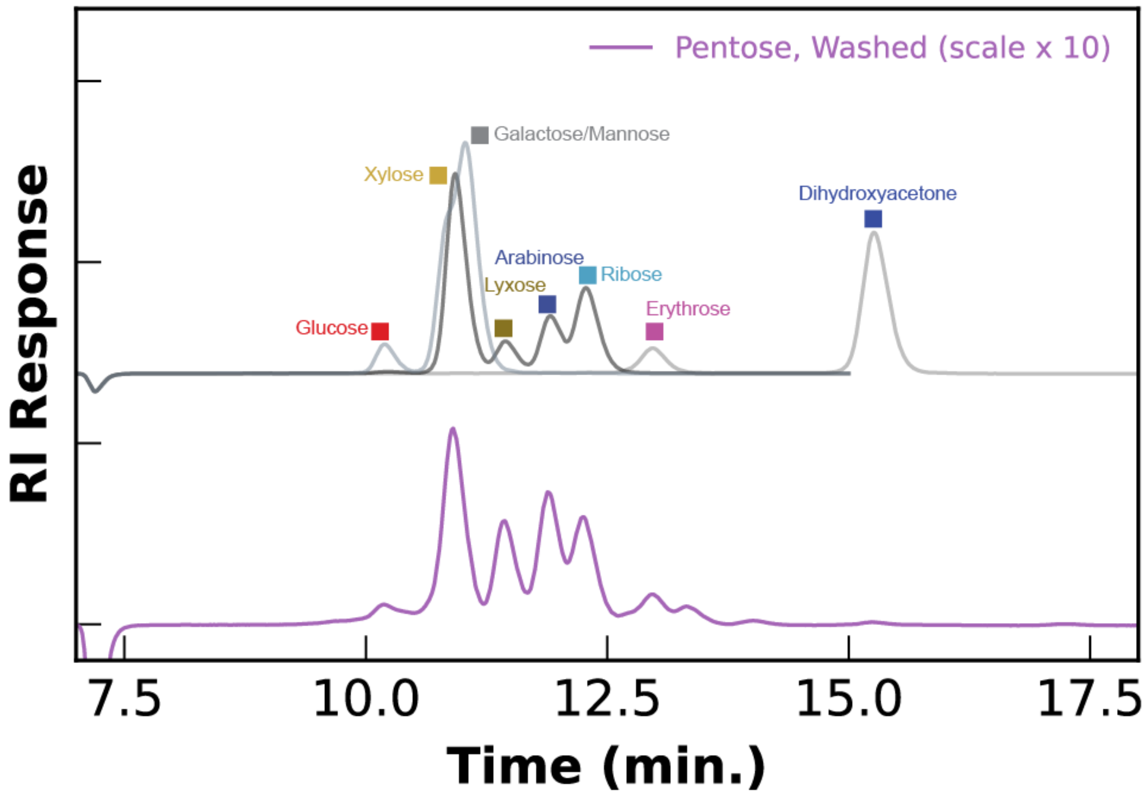

Fig. S5.
HPLC chromatogram of pentose and hexose sugars generated by **NHC-1** with formaldehyde as substrate. Sugars were prepared by removal of dioxane solvent and diethyl ether wash followed by dissolving in 10 mL H_2O. Comparison to monosaccharide standards shown (grey traces, upper). Positive ID is not possible for such measurements, as complete monosaccharide separation in HPLC is not always achievable. Therefore, secondary chromatographic confirmation by GC(-MS) of acetylated sugars and finally anomeric proton peaks by ^{1}H NMR combined with mechanistic interpretation allows for robust validation of aldose products.

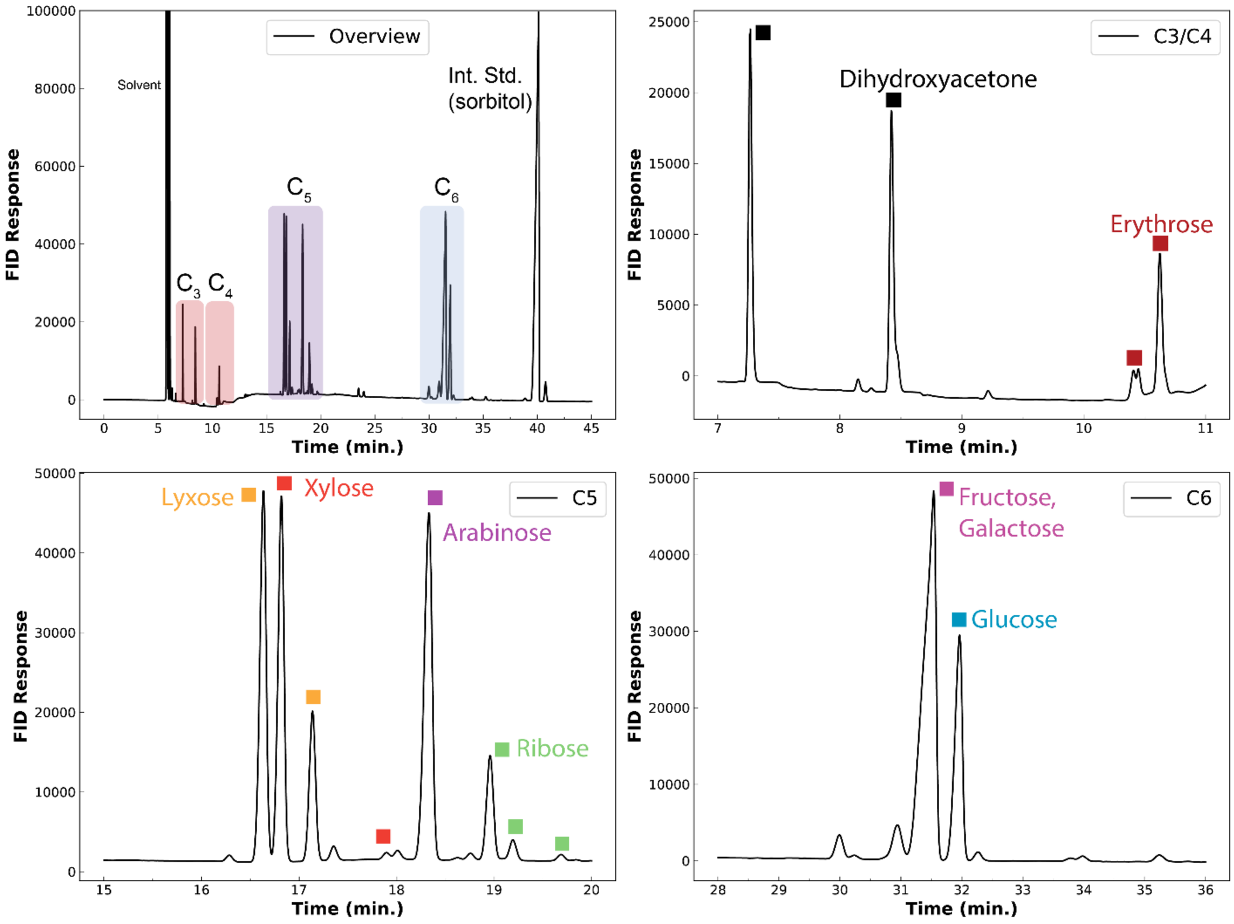

Fig. S6.
Annotated gas chromatograms of acetylated sugar products for reader scrutiny of following data. Regions utilized for quantitative analysis of triose, tetrose, pentose and hexose are shown. Some peaks associated with commercial (acetylated) monosaccharide standards are indicated. Multiple peaks apparent in standard mixtures are possibly different conformers/structural isomers or minor impurities.

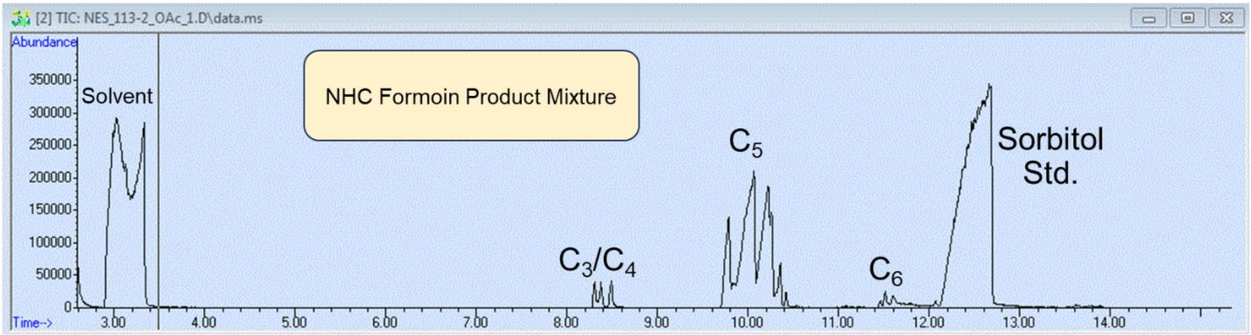

Fig. S7.
GC-MS total ion counts trace for acetylated NHC formoin product with sorbitol as an internal standard. Good separation by mass was achieved as in the previous GC method.

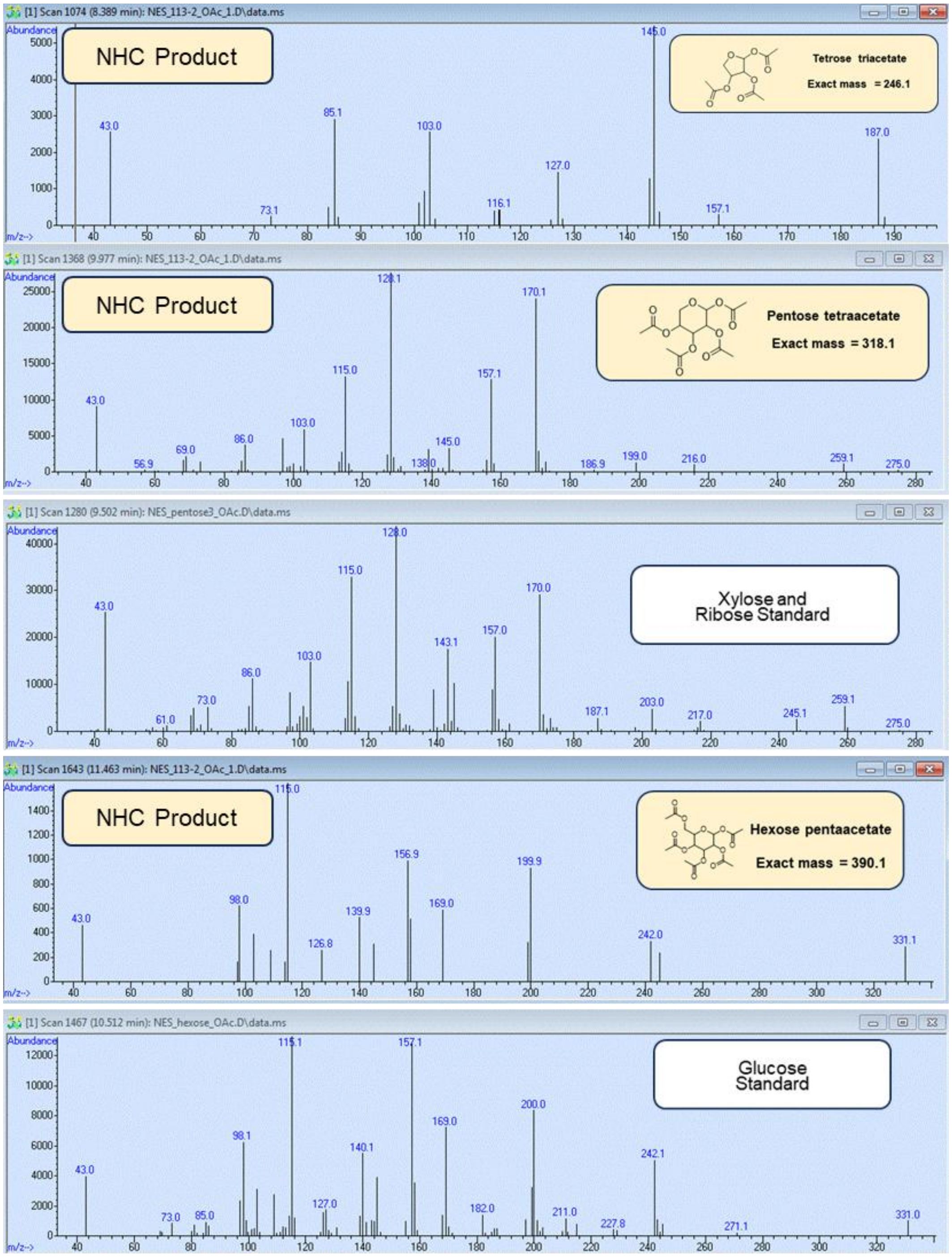

Fig. S8.
Mass analysis for acetylated NHC formoin product. Discrepancy of parent ion with expected m/z is consistent with acetate group fragmentation (m/z = 59). NHC pentose and hexose product peak mass chromatograms match well with fragmentation patterns of commercial standards prepared in the same fashion.

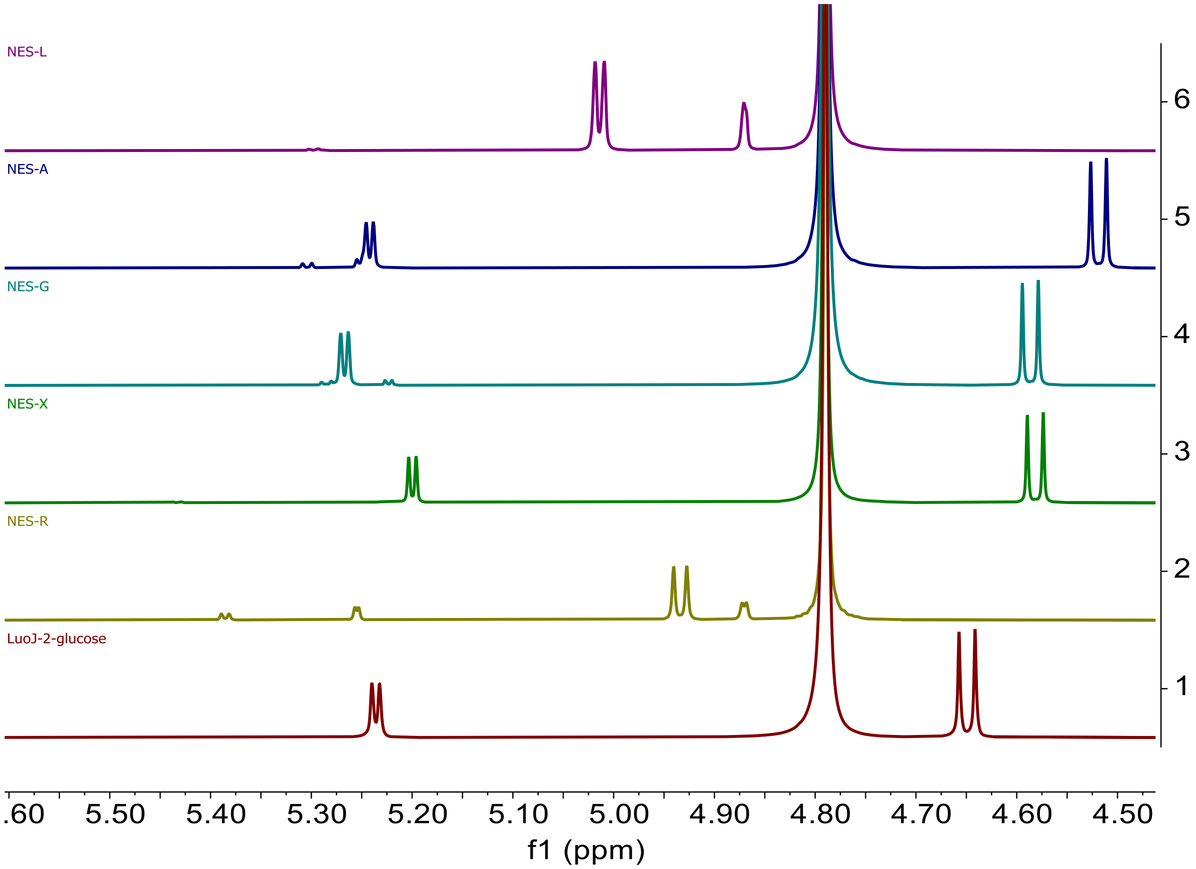

Fig. S9.
^1H NMR (500 MHz, D_2O) of aldose standards. L: Lyxose; A: Arabinose; G: Galactose; X: Xylose; R: Ribose; Glucose. Anomeric proton peaks are characteristic of the monosaccharide in question and may be used to assist in identification of simple mixtures. Large solvent peak at 4.78 ppm is water.

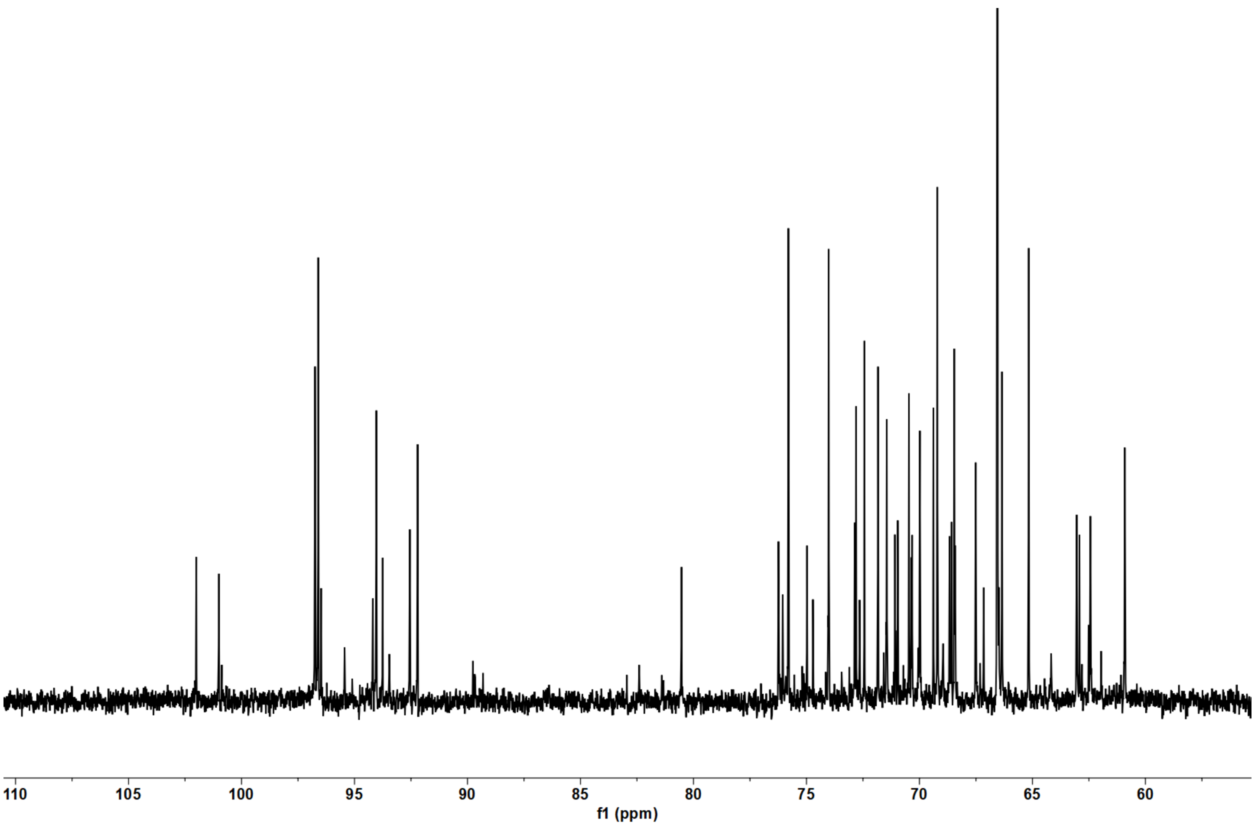

Fig. S10.
^{13}C NMR (126 MHz, D_2O) of pentose/hexose product showing distinct carbohydrate anomeric carbon peaks from 88-105 ppm. Backbone carbon signals ranged from 60 to 78 ppm.

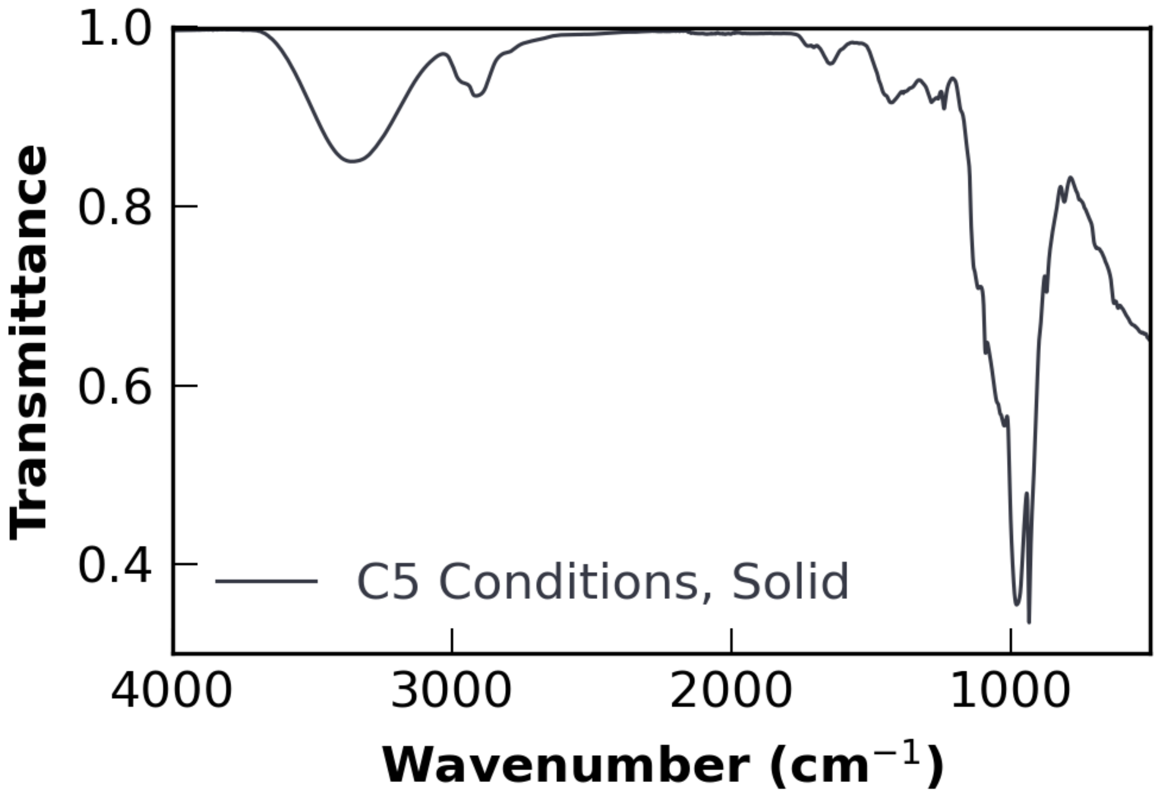

Fig. S11.
IR spectrum of formoin sugar product from pentose optimized conditions.

Table S2.
Essential IR peak assignments for carbohydrate product.

n (cm^{-1})	Assignment
ca. 1000-1050	C-O (ether)
ca. 2920	C-H
ca. 3400	O-H

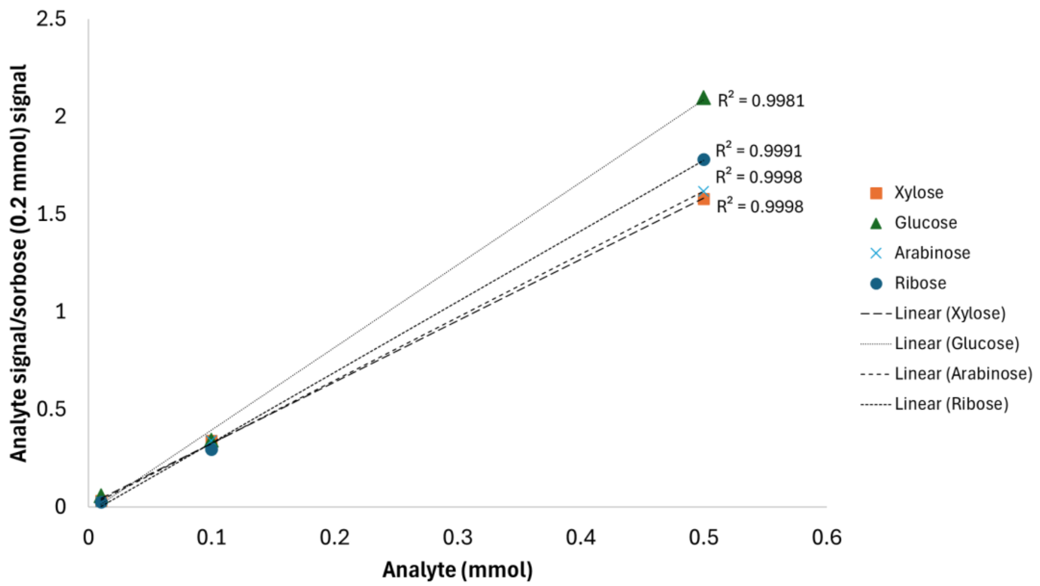

Fig. S12.
Example calibration curve of acetylated sugars compared to sorbitol internal standard, displaying similar response factors for pentoses and differing for hexose.

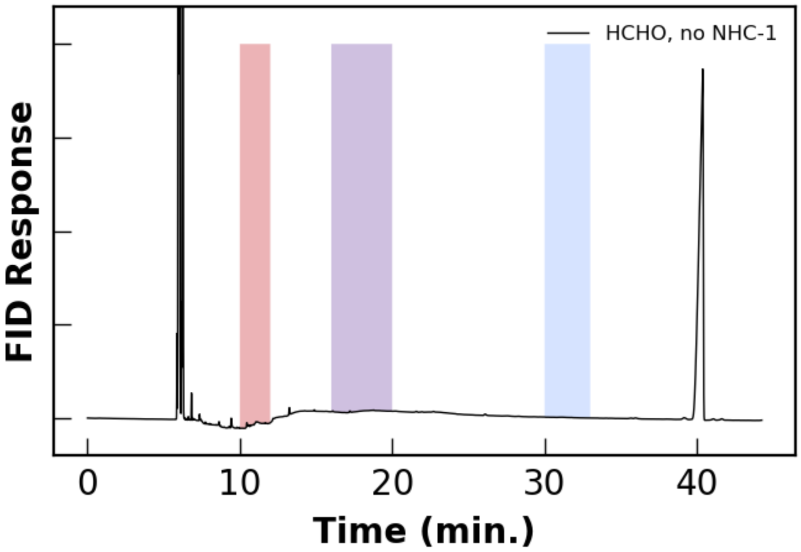

Fig. S13.
Control reaction without NHC catalyst: absence of NHC does not yield any quantifiable products along the lines of those produced in the presence of NHC and formaldehyde. There are some very minor (not quantified) peaks that may represent oligomers of formaldehyde, but there is no discernible spontaneous formation of carbohydrate-associated peaks.

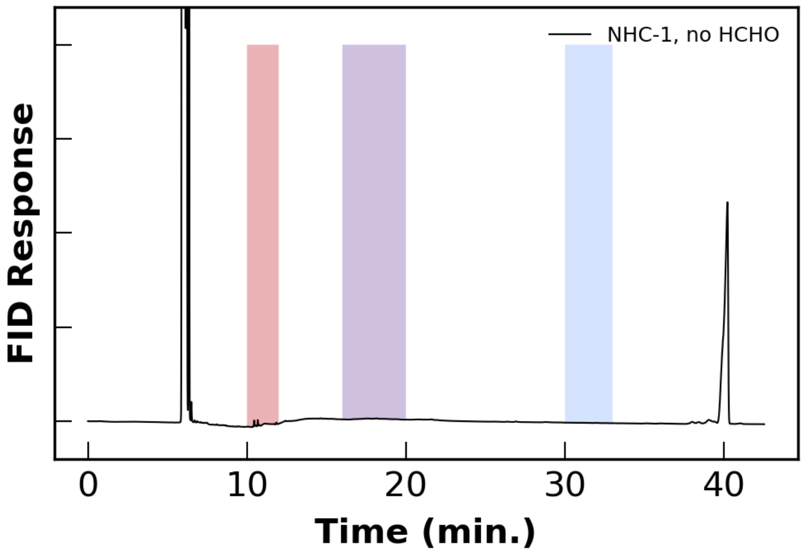

Fig. S14.
Control reaction without formaldehyde: Conversely, the absence of formaldehyde (or other suitable aldehyde) substrate in the presence of NHC and dioxane does not yield any suitable products, indicating that the formaldehyde is indeed necessary for generation of products.

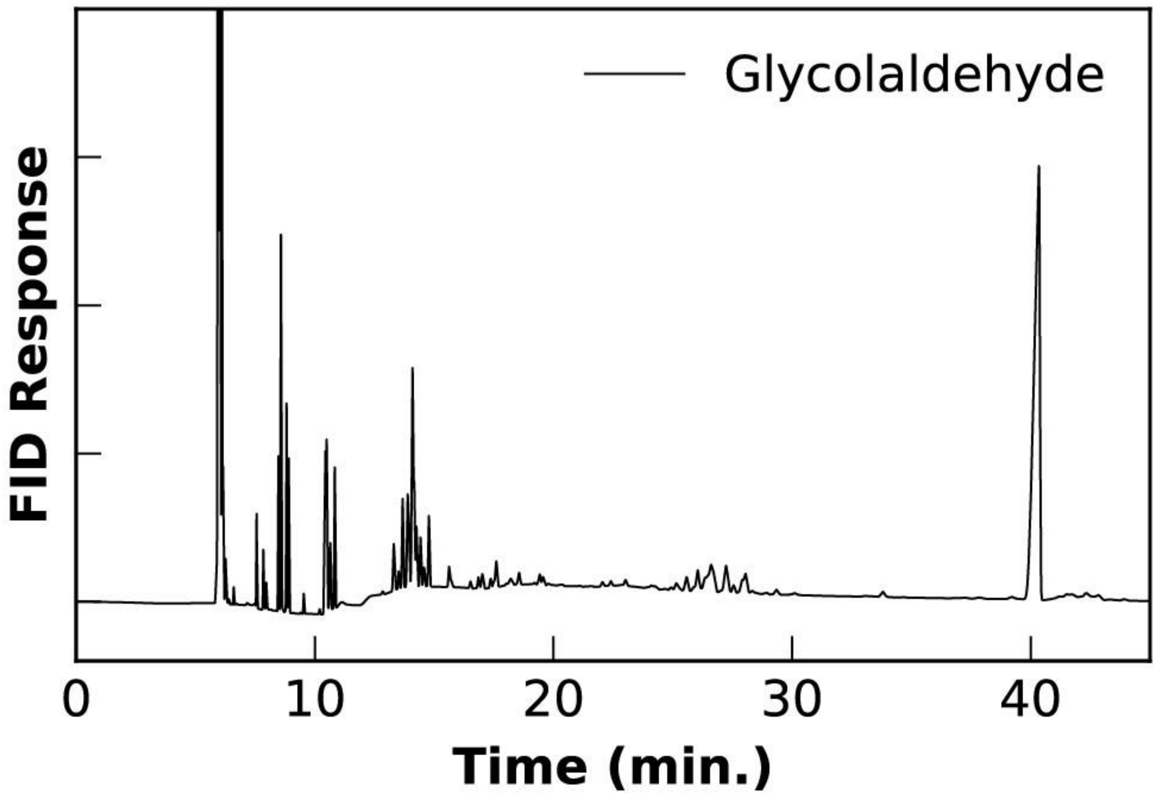

Fig. S15.
NHC-1 with glycolaldehyde (C_2 aldose) as the substrate under pentose conditions. Several products are generated with intermediate retention times compared to the known aldopentose and aldotetrose standards and are currently unidentified but expected to be indicative of C_2-C_n coupling leading to ketoses and branched sugars.

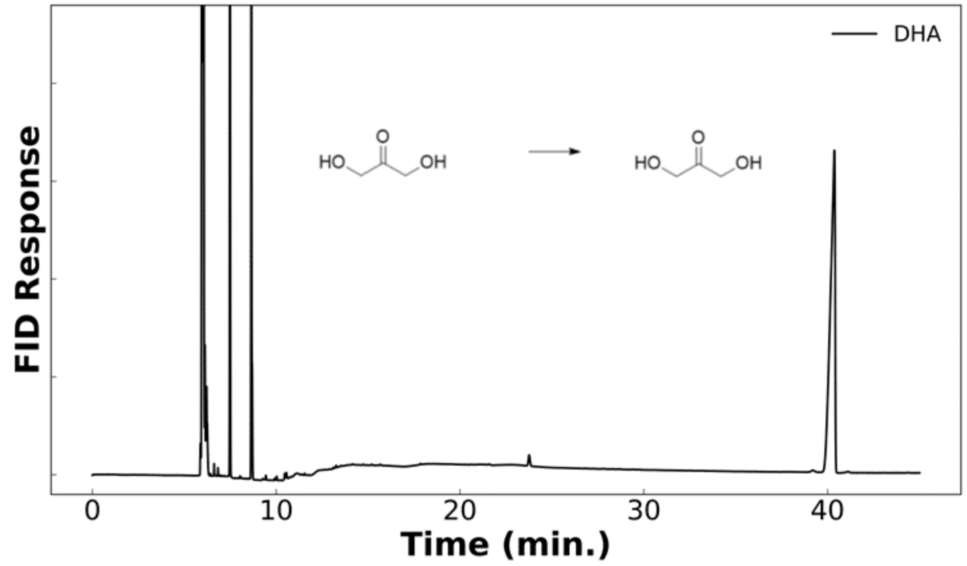

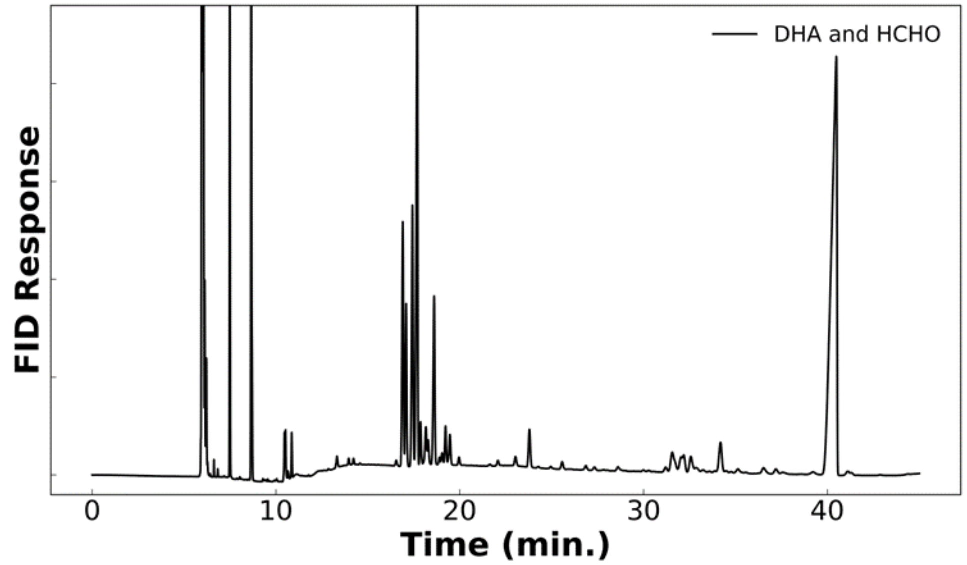

Fig. S16.
Top: **NHC-1** with dihydroxyacetone (DHA) as the sole substrate under pentose reaction conditions. No unique products are generated. Bottom: DHA with paraformaldehyde (PF) addition. Most DHA remains unreacted and the remaining product distribution is nearly identical to that of pentose conditions for PF alone, suggesting that PF is the preferred substrate and that DHA is a terminal and nonproductive reaction product.

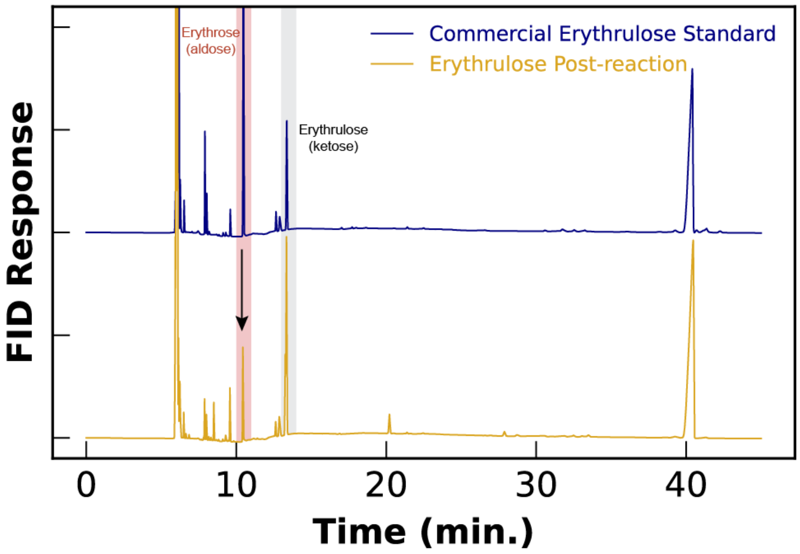

Fig. S17.
NHC-1 with erythrulose (C_4 ketose) as the substrate under pentose conditions. Several products are generated with intermediate retention times compared to the known aldopentose and aldotetrose standards and are currently unidentified. Given the presence of other impurities in the original reactant, including the aldotetrose erythrose, any generated products are likely to have resulted from those species, leaving the majority of erythrulose intact. This is indicated by the relative reduction in the erythrose peak at 10.5 min relative to the content in the unreacted erythrulose (highlighted in red above).

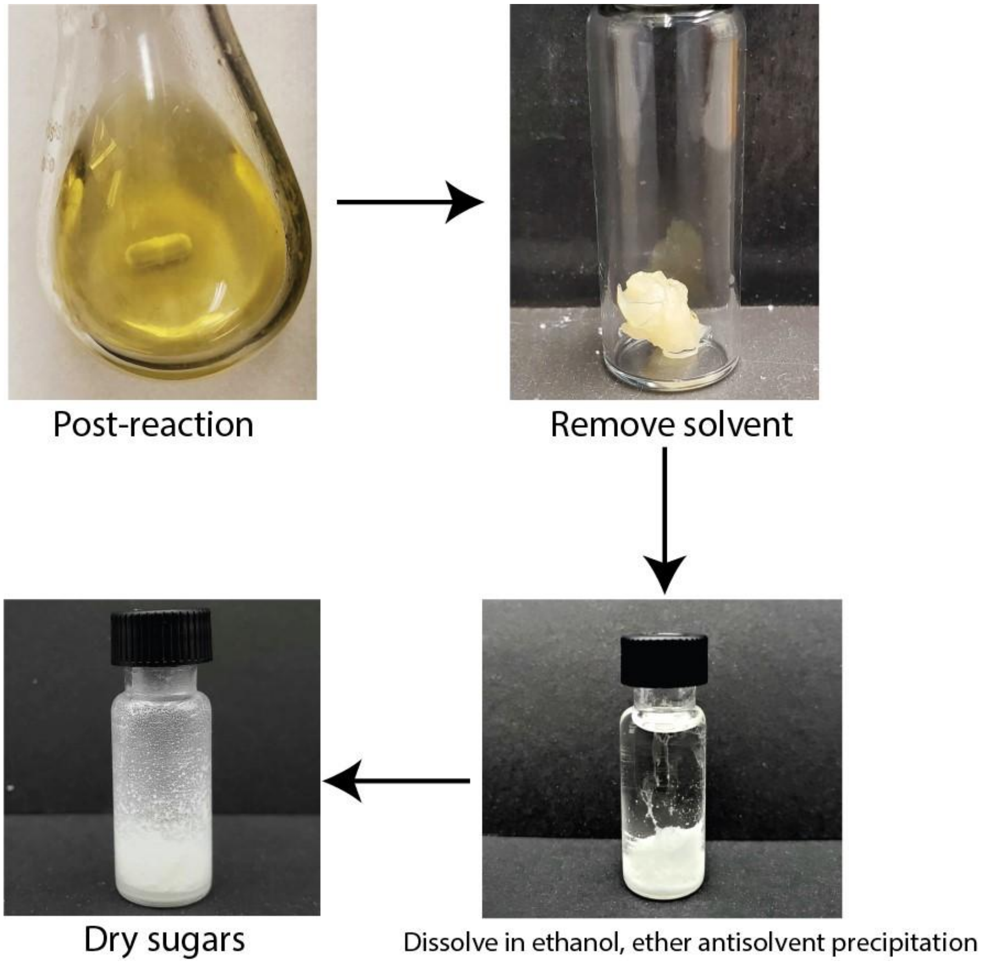

Fig. S18.
Processing of NHC formoin sugar product. Purified sugars are obtained by removing reaction solvent (e.g. dioxane), followed by a diethyl ether wash to remove NHC catalyst. The sugars are dissolved in minimal ethanol and precipitated with addition of cold ether. The dried sugars are quite hygroscopic and to some degree have a syrup-like consistency due to presence of lower order sugars, solvent persistence, and inconsistent co-crystallization behavior. Mass yield is similar to that expected based on carbon yield estimated by gas chromatography.

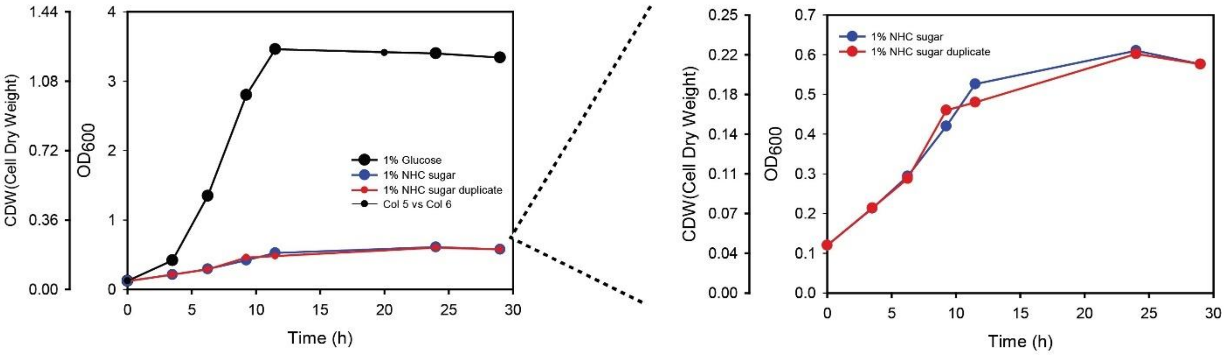

Fig. S19.
Bacteria growth in NHC formoin product and pure glucose substrates. Strain: *E. coli* BW25113. Carbon source: Pure glucose: Glucose 1% (10 g/L). NHC sugar: NHC sugar 1% (10 g/L). Media: M9 minimal media.

Table S3.
Growth rates derived from Fig. S18.

Specific growth rate (h^{-1})	
Pure glucose	0.347
NHC sugar	0.134
NHC sugar duplicate	0.142

Table S4.
M9 minimal media composition.

Component	M9 minimal
NaCl	8.5mM
KH_2PO_4	22 mM
Na_2HPO_4	48 mM
NH_4Cl	19 mM
$MgSO_4$	2mM
$CaCl_2$	0.1mM

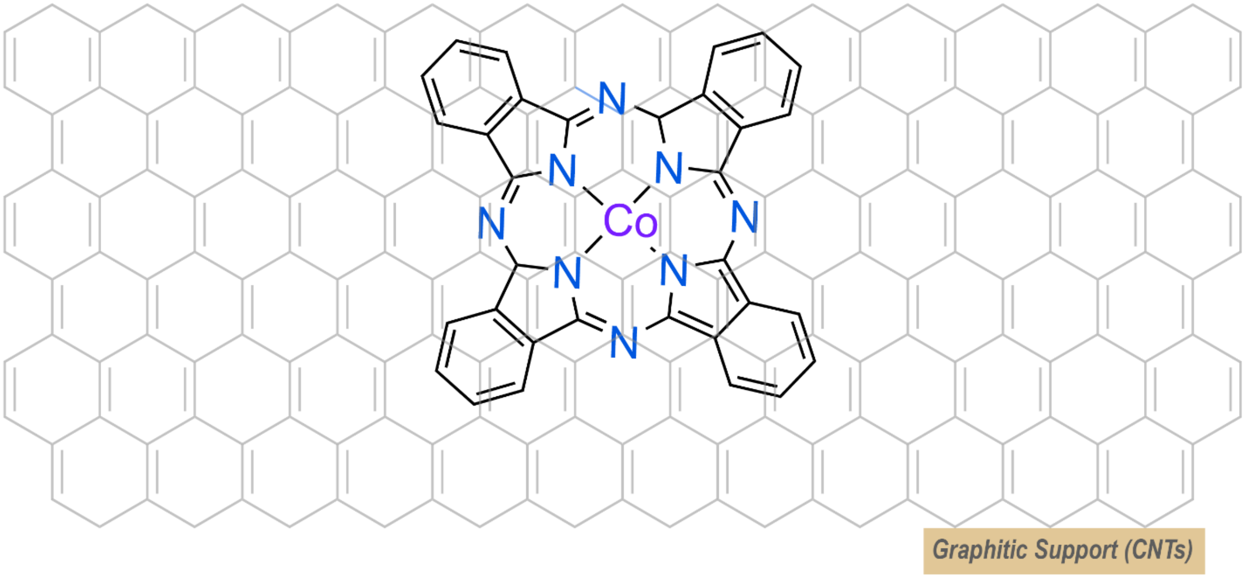

Fig. S20.
Schematic of the hypothesized structure of adsorbed cobalt phthalocyanine on carbon nanotube substrate.

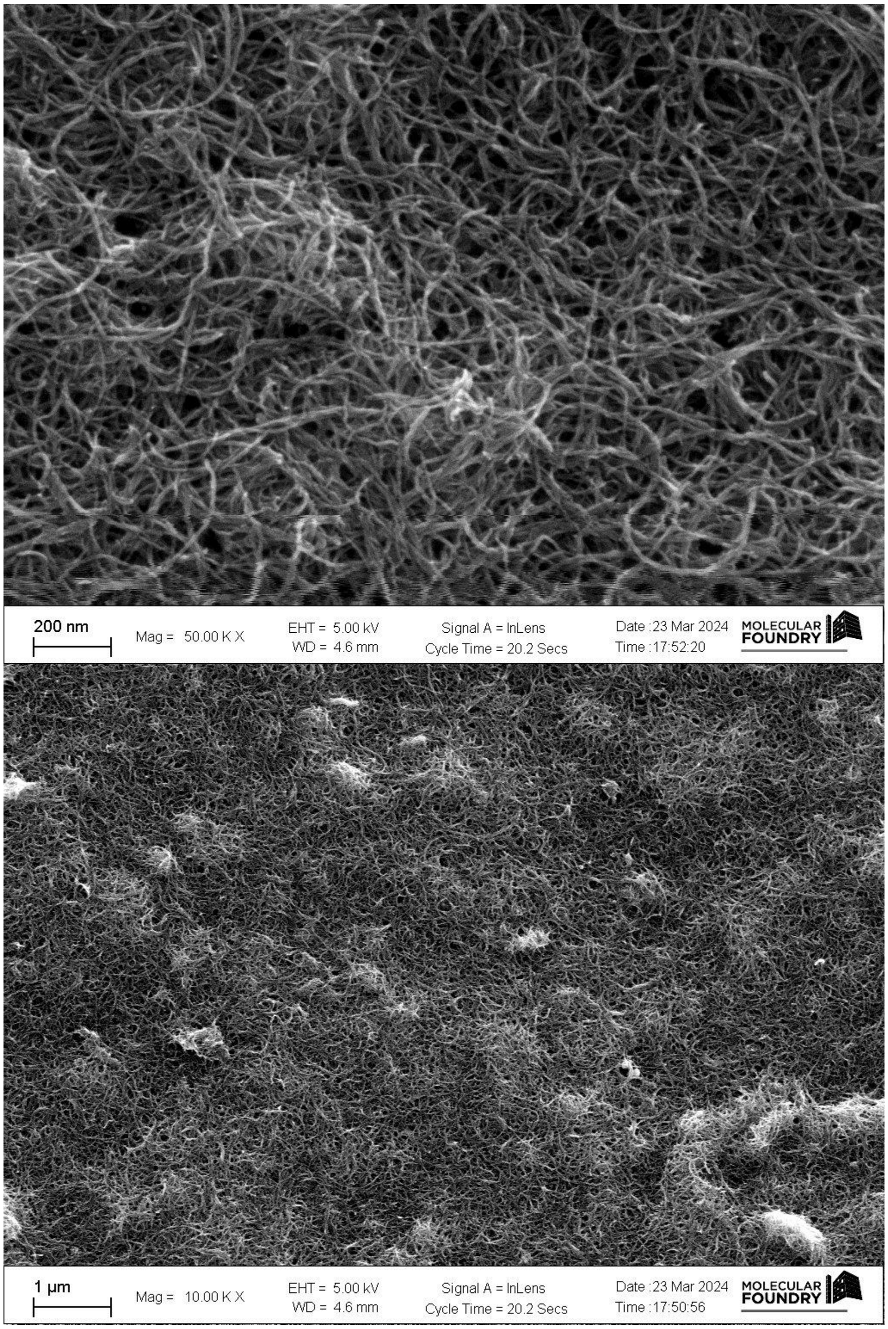

Fig. S21.
SEM of CoPc/CNT electrode showing tortuous/net-like morphology. Some bundling of CNTs is inevitable (lower) but general film morphology is smooth. No visible crystallites of CoPc remain after washing.

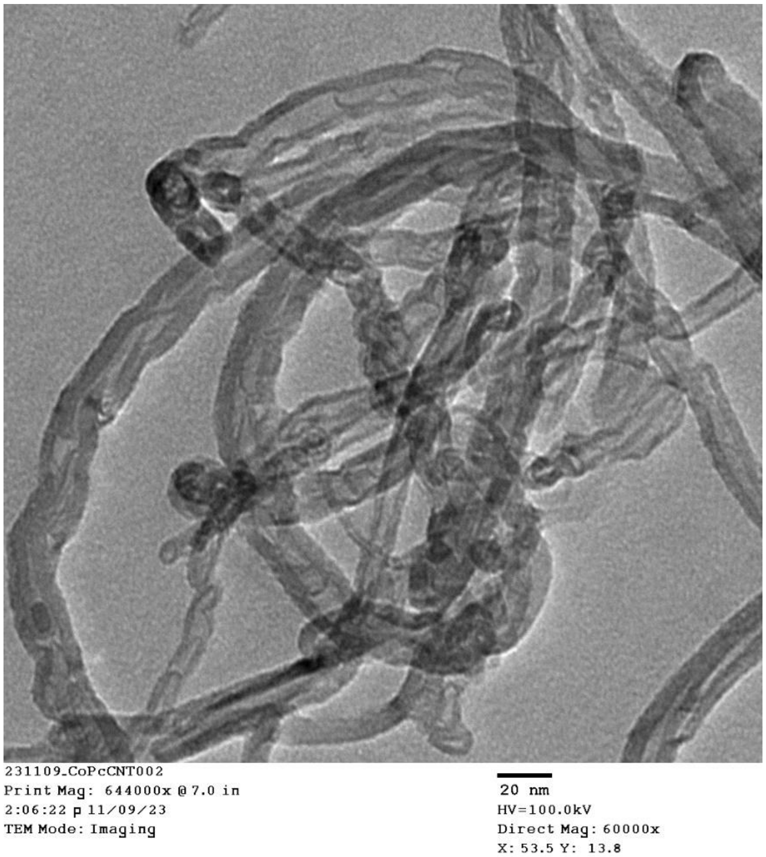

Fig. S22.
TEM image of CoPc/CNT. O.D. of MWCNTs is typically between 10-20 nm.

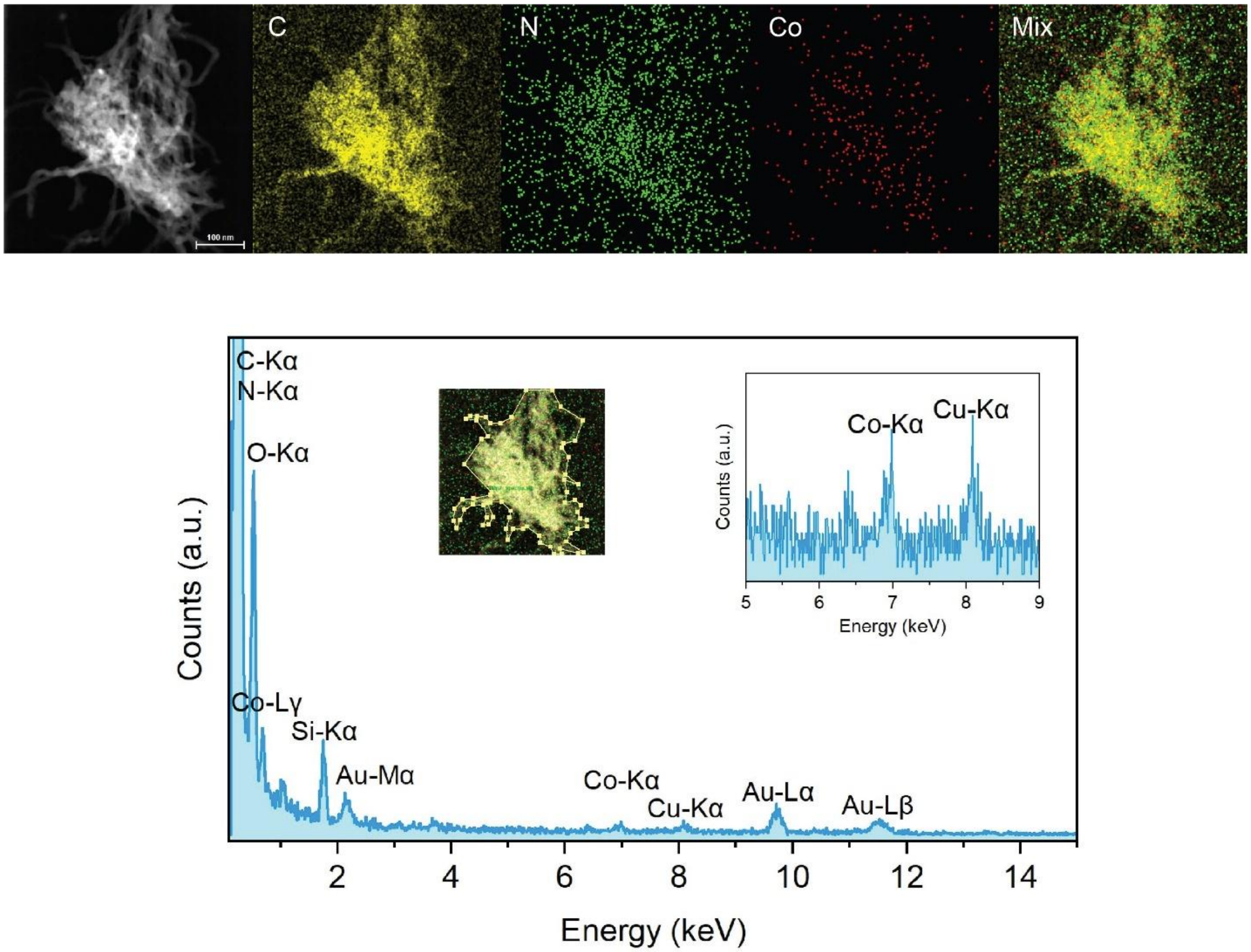

Fig. S23.
HAADF-STEM and corresponding element mapping (STEM-EDS analysis) of CoPc/CNT showing localization of Co sites on CNTs. The Au signals are from the Au TEM grid. The Cu signals are commonly from clips and washers used to fixate the grid on the TEM holder.

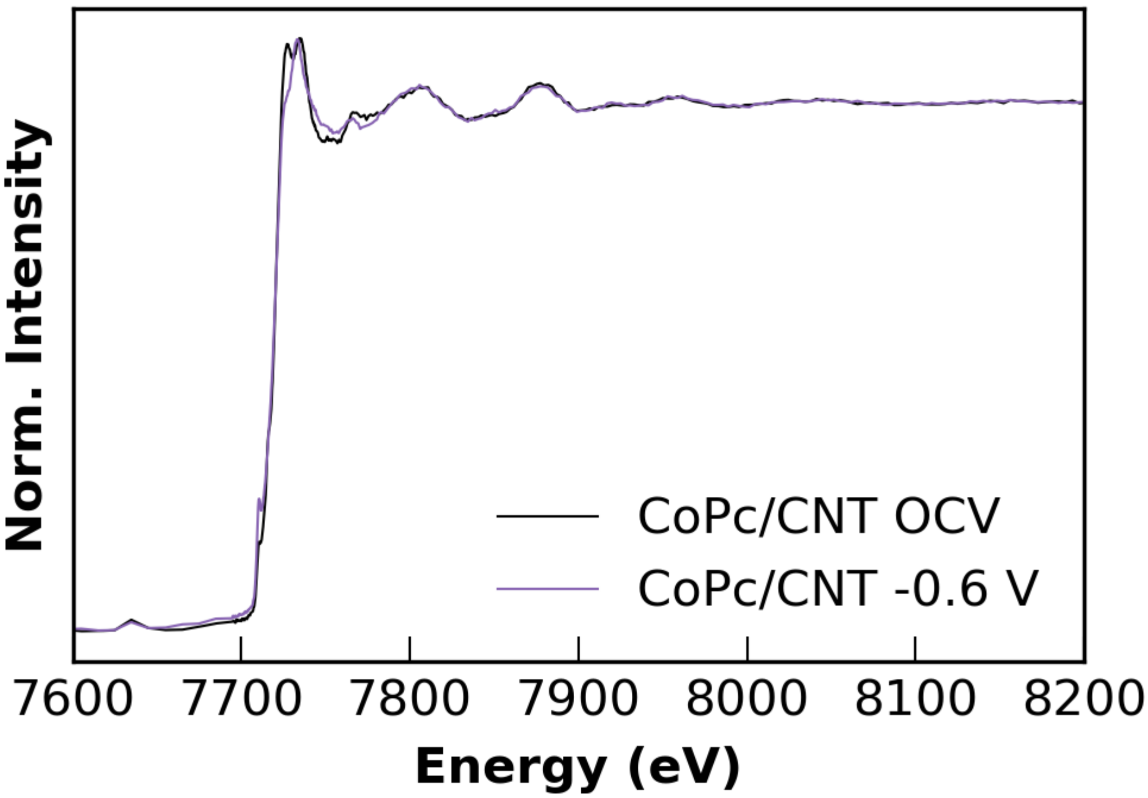

Fig. S24.
Co K-edge XAS of CoPc/CNT catalyst. Under bias with CO_2 flow, edge features are diminished due to electronic conjugation and donation from the electrode. The feature at 7710 eV is assigned in literature to the 1s → 3d transition which increases under bias, possibly as a result of molecular distortion caused by adsorption to the CNT or axial binding of CO under bias (6, 7). The feature at 7715, caused by 1s → 4p, similarly decreases under bias due to this effect. In summary, there are clearly identifiable XANES features that distinguish the highly dispersed and graphite-supported CoPc from its aggregated precursor and indicate electronic conjugation with the substrate.

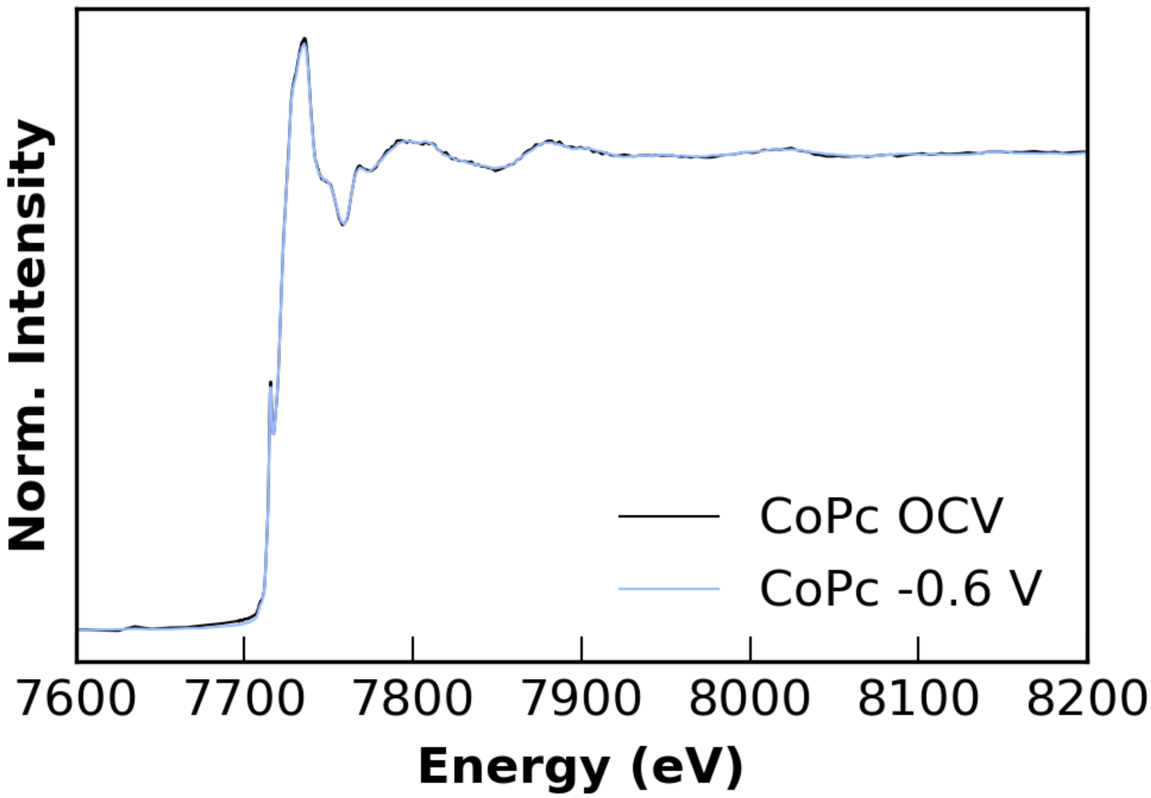

Fig. S25.
Co K-edge XAS of CoPc powder drop-cast onto Sigracet 39AA paper. With bias under CO_2 flow, edge features do not change significantly. The species are virtually unaffected by applied bias due to their insulated nature.

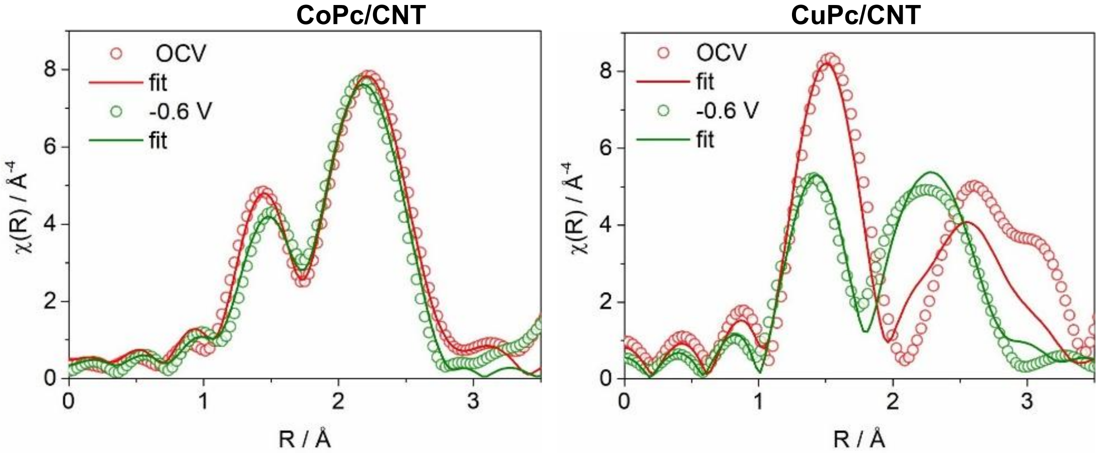

Fig. S26.
EXAFS analysis. Our CNTs contain small amounts of cobalt from their synthesis, despite conventional washing protocols. The Fourier-transformed EXAFS spectra of our CoPc-CNT catalyst shows a feature near 2.2 A, very similar to Co foil, whereas cobalt oxides tend to show a feature near 2.5 A, indicating the presence of metallic Co rather than cobalt oxides.
We investigate the potential agglomeration of CoPc by determining the ratio of CoPc:Co at OCP and at -0.6 V vs. RHE by EXAFS fitting. For a first-shell fit, we use three scattering paths for the CoPc phase (@-N-@, @-C-@, @-C-N-@), and one path for the Co phase (@-Co-@). We weigh all CoPc paths by the fraction x, and the Co path by (1-x). We determined S_0^2 from the data at OCP (which has higher quality due to the absence of bubble formation) by iteratively adjusting x and S_0^2 until finding the best fit (Table S5). As the measurement at -0.6 V was taken shortly after the OCV one, we use the energy shift ΔE determined for the OCV sample for the sample under applied potential, varying merely x to see if it has changed (Table S6).
In the tables, ΔR1 and $\sigma^2$1-3 are the relative adjustment to the half path length and the Debye-Waller factors of the CoPc phase, while ΔR2 and $\sigma^2$4 are those of the Co phase. Bold parameters are held fixed. The best fits for the two samples are highlighted in green.
Comparison may be drawn for the reader with copper phthalocyanine stability, CuPc, measured under similar conditions at CHESS. There is significant restructuring of the catalyst under negative bias, which can be attributed to demetallization. Cobalt phthalocyanine, by comparison, shows little comparable restructuring, and therefore is a more stable electrocatalyst for CO_2RR.
In total, this EXAFS analysis indicates that the initial CoPc/CNT sample is made of approximately 86% monodispersed CoPc and 14% metallic-like Cobalt. During and after strong applied bias, we see a reduction in single-atom fraction to approximately 69%. This can be contrasted with the metallo-analogue CuPc, which shows significant restructuring and demetallation under similar electrochemical conditions, as elaborated in recent work (8). This helps support the concept that has been presented in several reports of the CoPc/CNT system that isolated and immobilized CoPc molecules are predominantly active in the electrocatalytic system.

Table S5: EXAFS fitting parameter for CoPc-CNT at OCV.

**reduced χ2**	**R-factor**	**S_0^2**	**x**	**ΔE**	**ΔR1**	**$\sigma^2$1**	**$\sigma^2$2**	**$\sigma^2$3**	**$\sigma^2$4**	**ΔR2**
1231.862	0.227591	**1**	**1**	-3.48868	-0.04086	0.008543	0	0.016399	0.003	0
32.73458	0.005527	**1**	**0.9**	8.028539	-0.00319	0.009399	0.001914	0.006022	0.001894	-0.00834
30.91619	0.00523	**1**	**0.89**	8.116315	-0.00264	0.009364	0.002263	0.005794	0.002561	-0.00754
30.72433	0.005229	**1**	**0.88**	8.205363	-0.00209	0.009324	0.002608	0.005561	0.003177	-0.00679
31.85964	0.005471	**1**	**0.87**	8.292653	-0.00153	0.009279	0.002948	0.00532	0.003749	-0.00607
62.12525	0.011313	**1**	**0.8**	8.868822	0.002757	0.008874	0.00536	0.00336	0.00691	-0.00198
35.75998	0.003005	**0.9**	**0.88**	8.262741	-0.00251	0.007689	-0.00082	0.004495	0.002207	-0.00793
17.41736	0.002071	**0.87**	**0.88**	8.436002	-0.00236	0.007309	0.001243	0.005918	0.002052	-0.00714
17.40117	0.001953	**0.86**	**0.88**	8.454199	-0.00238	0.00715	0.001123	0.005938	0.001959	-0.00718
17.56322	0.001858	**0.85**	**0.88**	8.478579	-0.00238	0.006992	0.001002	0.005961	0.001863	-0.00721
21.32601	0.001729	**0.8**	**0.88**	8.57089	-0.00245	0.006189	0.000368	0.006066	0.001372	-0.00746
12.38891	0.001953	**0.86**	**0.88**	**8.45**	-0.00239	0.007149	0.001123	0.005937	0.001959	-0.00719
10.03342	0.001769	**0.86**	**0.87**	**8.45**	-0.00215	0.007082	0.001387	0.005554	0.002509	-0.00668
9.308003	0.001825	**0.86**	**0.86**	**8.45**	-0.00191	0.007012	0.001643	0.00516	0.003024	-0.00621
9.737058	0.002084	**0.86**	**0.85**	**8.45**	-0.00172	0.006939	0.001901	0.004798	0.003526	-0.00576

Table S6: EXAFS fitting parameter for CoPc-CNT at -0.6 V.

**reduced χ2**	**R-factor**	**S_0^2**	**x**	**ΔE**	**ΔR1**	**$\sigma^2$1**	**$\sigma^2$2**	**$\sigma^2$3**	**$\sigma^2$4**	**ΔR2**
48.53916	0.010671	**0.86**	**0.75**	**8.45**	-0.00846	0.008318	0.011152	0.004115	0.007465	0.001573
29.16597	0.006075	**0.86**	**0.7**	**8.45**	-0.00802	0.00754	0.012044	0.002387	0.008905	0.002297
28.34267	0.005722	**0.86**	**0.69**	**8.45**	-0.00792	0.007373	0.012237	0.002157	0.009177	0.002436
28.45899	0.005552	**0.86**	**0.68**	**8.45**	-0.0078	0.007204	0.01242	0.001924	0.009445	0.002573
34.12887	0.006065	**0.86**	**0.65**	**8.45**	-0.00745	0.006685	0.012931	0.001209	0.010217	0.00297

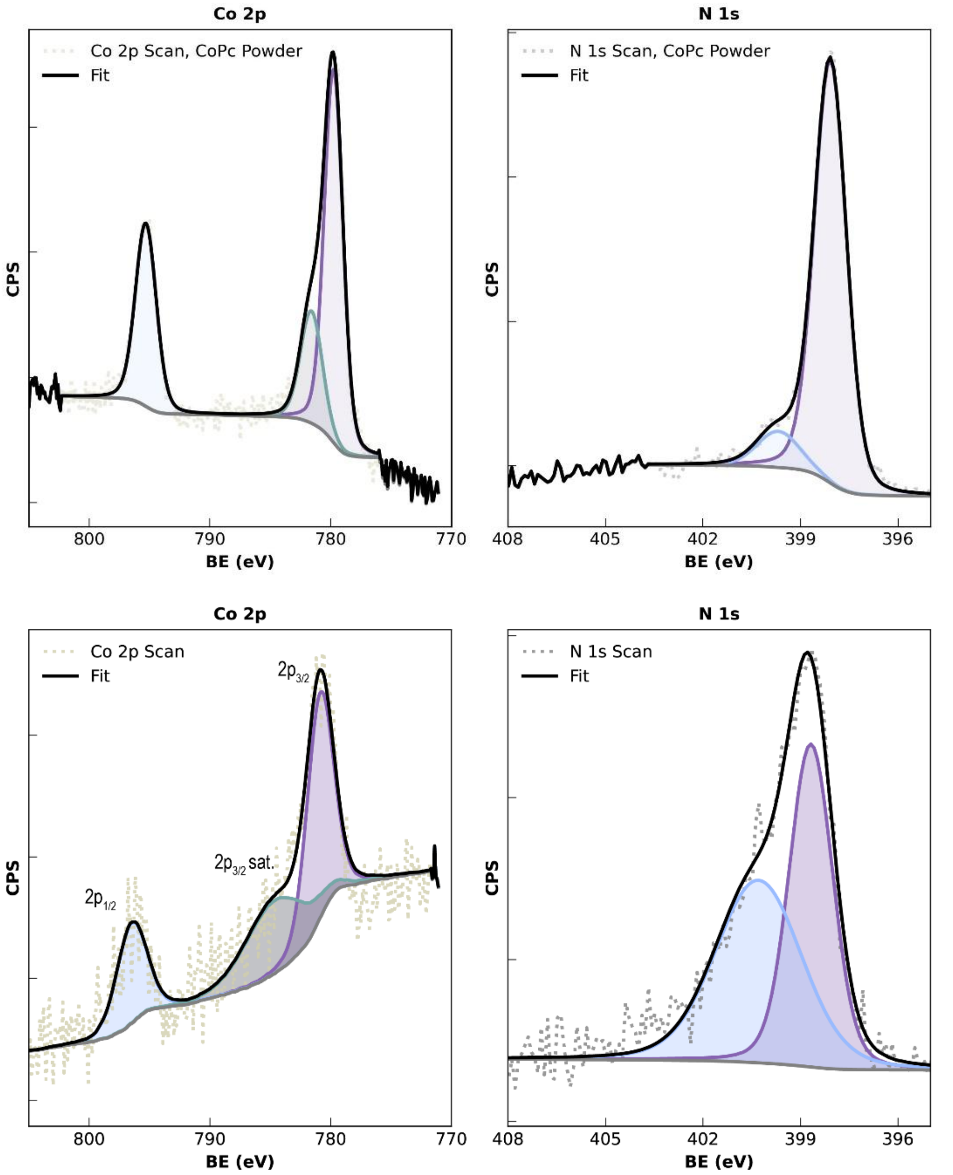

Fig. S27.
XPS analysis of CoPc Powder (top) vs. CoPc/CNT, post-catalysis (bottom). Left: Co 2p Right: N 1s. The higher binding energy (400.4 eV) shoulder has been reported as indicative of distinct nitrogen speciation in the strained phthalocyanine molecule on CNT support (9).

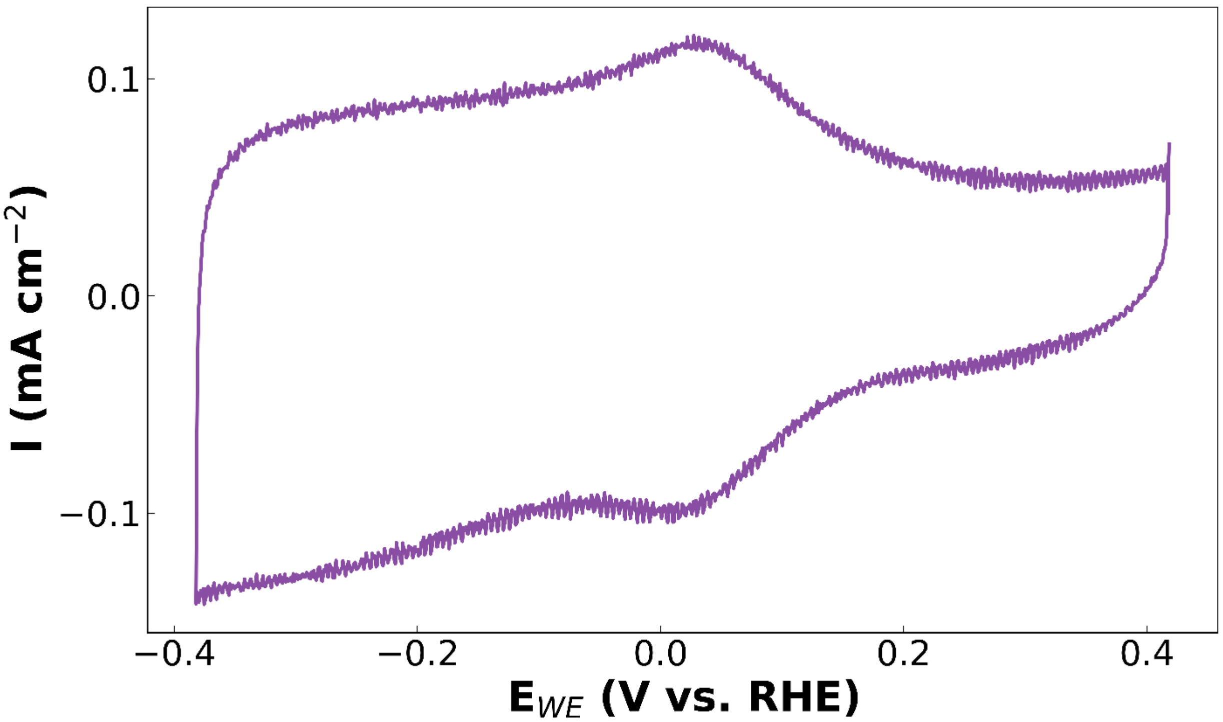

Fig. S28.
CV of CoPc/CNT electrode showing a reversible $Co^{II/I}$ redox wave at ca. 0.05 V vs. RHE.

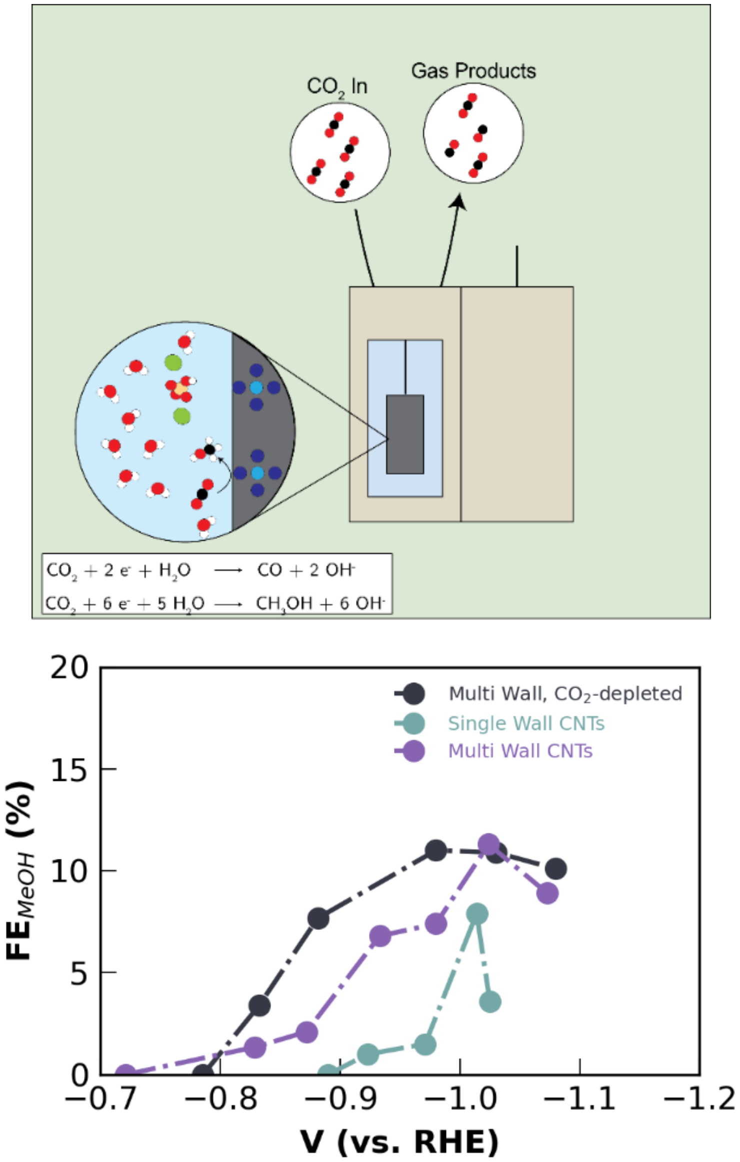

Fig. S29.
H-cell schematic (top) and performance (bottom) of several CoPc/CNT inks. Comparison was drawn between multi-walled and single-wall CNTs and for utilizing 2 sccm CO_2 flow rate without stirring to deplete the electrolyte and improve local CO concentration. The H-cell provided much lower tunability of mass transport factors, and the decision was made to translate the system into the flow cell using a gas diffusion electrode (GDE).

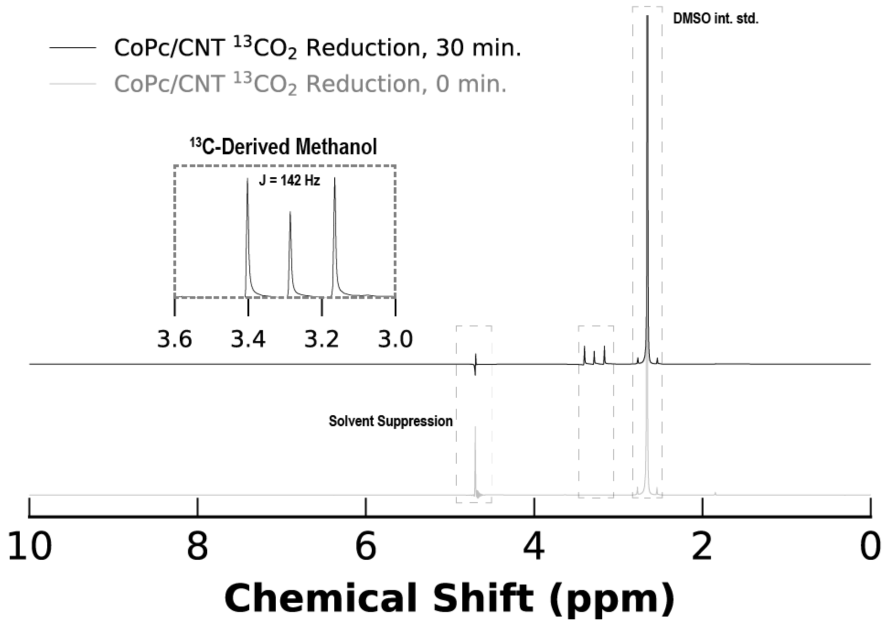

Fig. S30.
The $^{13}C{-}^{1}H$ coupling constant for ^{13}C-derived methanol was observed as J=142 at a chemical shift of 3.28 ppm in 9:1 H_2O:D_2O solvent, measured on a 600 MHz Bruker NMR spectrometer. No methanol was detected in the blank electrolyte (0.1 M $KH^{12}CO_3$) after poising at OCP for 30 minutes. After briefly purging the electrolyte with 99% $^{13}CO_2$ (Sigma Aldrich) for 15 minutes, cathodic bias at -1.01 V vs. RHE was applied with minimal stirring for 30 minutes. ^{1}H-NMR, shown below, reveals the production of methanol highly enriched in ^{13}C, as indicated by the doublet centered at 3.28 ppm. Some $^{12}CH_3OH$ is observed due to the non-isotope enriched CO_2-saturated bicarbonate electrolyte.

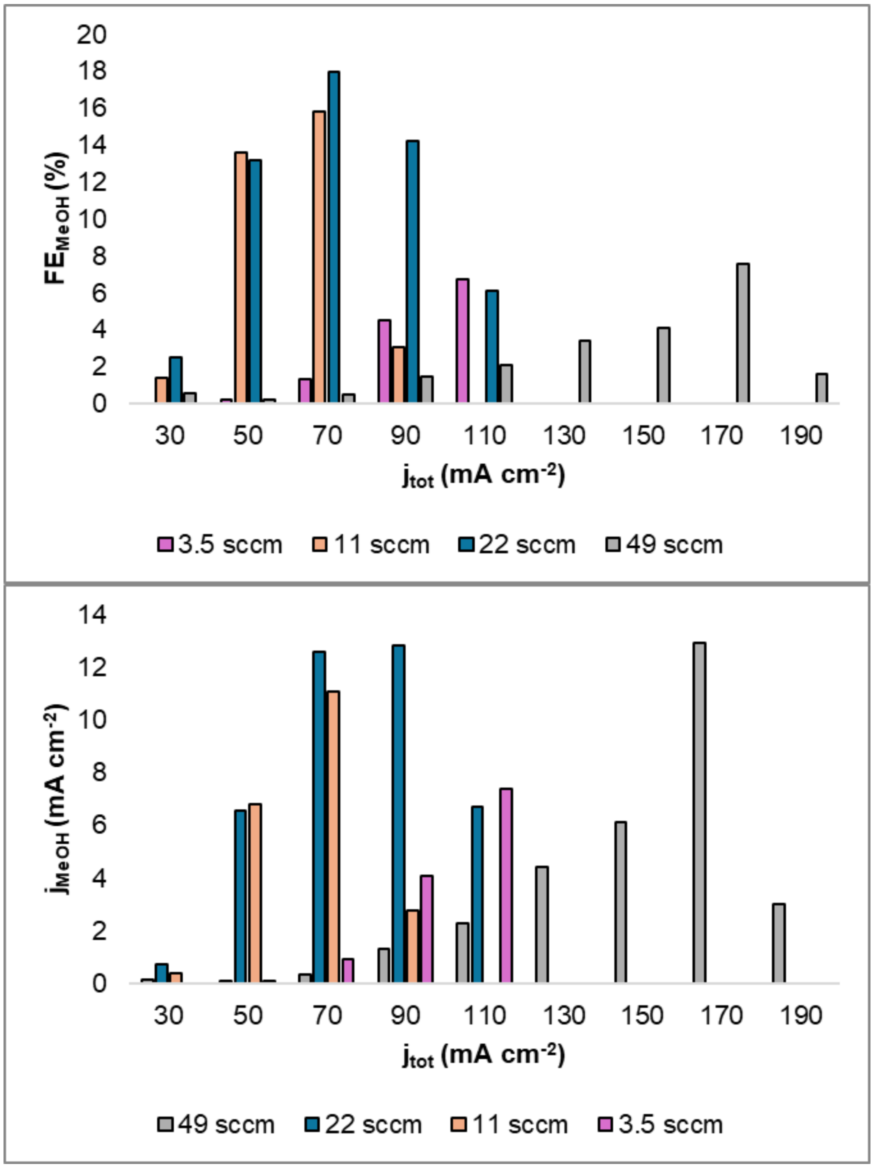

Fig. S31.
Flow cell results for adjusting total CO_2 flow rate as standard cubic centimeters per minute (sccm). Higher current densities could be accessed at higher flow rate, at the cost of faradaic efficiency, and the total methanol partial current density as a result was only slightly affected by these flow conditions.

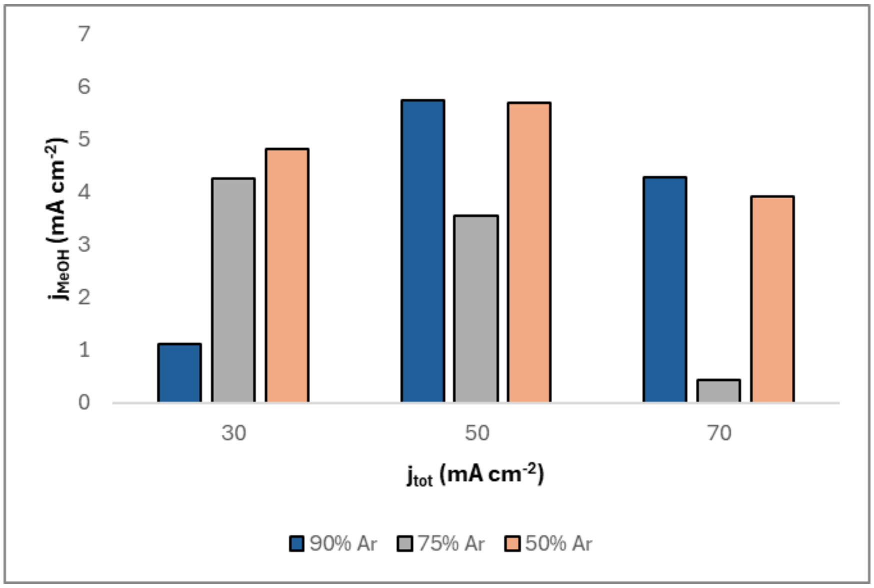

Fig. S32.
Flow cell results for adjusting feed gas composition. CO_2 input was mixed with an inert gas, argon, to decrease the local CO_2 concentration.

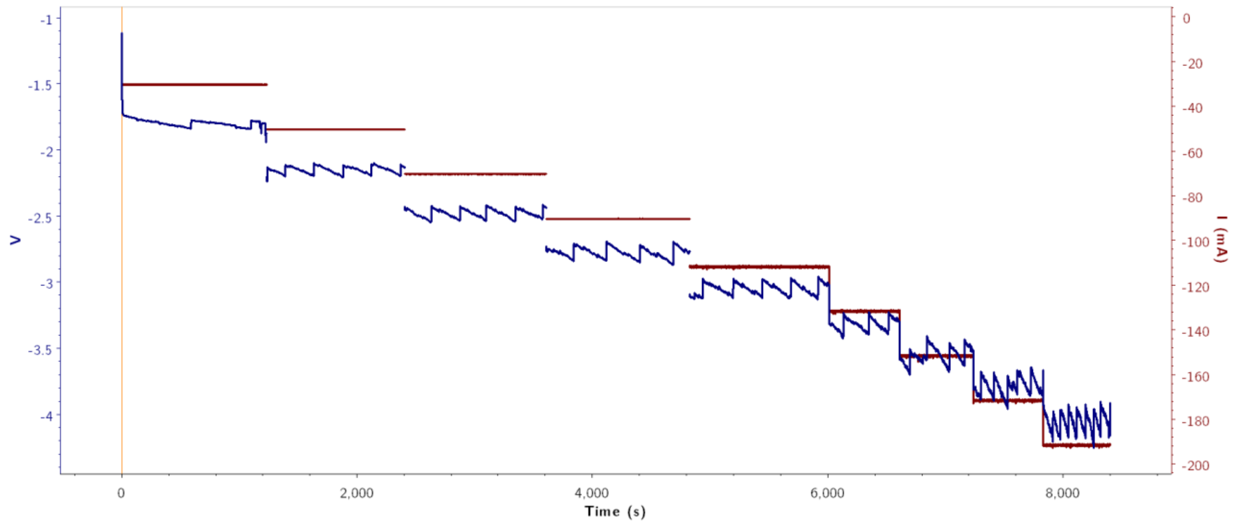

Fig. S33.
Example CoPc/CNT flow cell I + E_{WE} vs. t traces, double loading experiments. Oscillatory E_{WE} is commonly observed in this system, which we surmise to be a combination of bubble formation/release and consequent alternating CO_2 and CO local atmosphere caused by trapped gas product, allowing for improved further reduction to methanol.

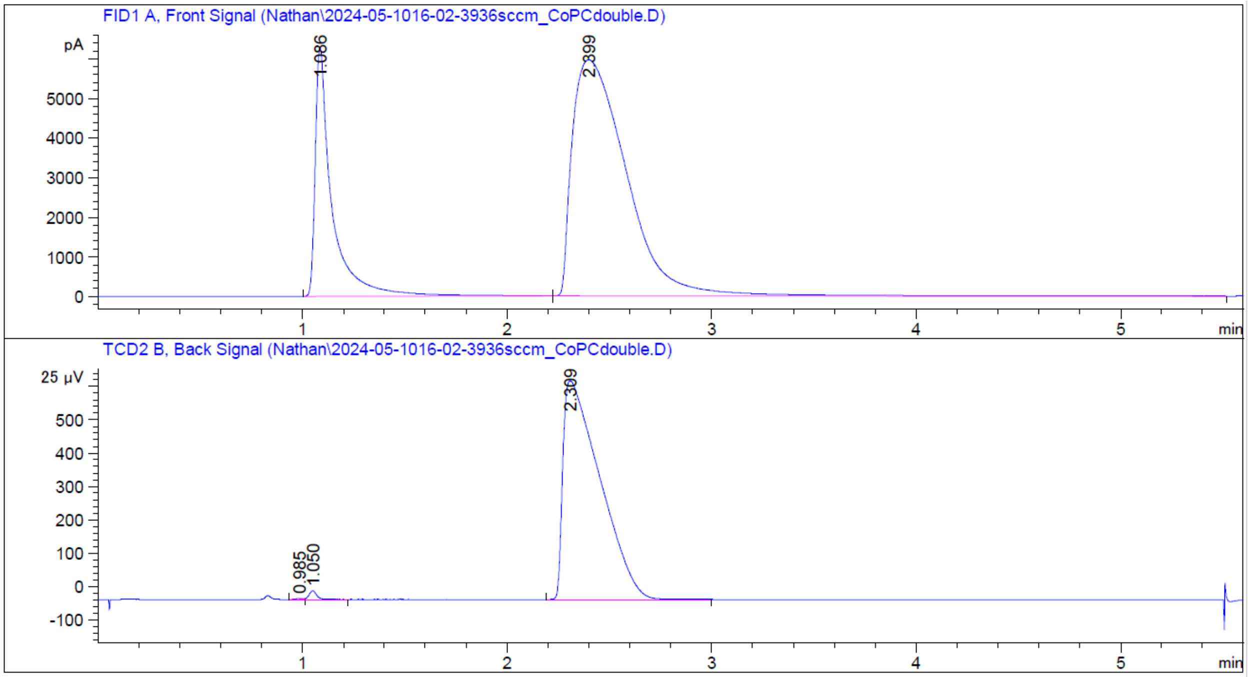

Fig. S34.
GC traces of CO_2RR electrolysis product in the flow cell. Peak at 1.086 min. in FID is CO product, while the TCD peak at 0.87 indicates hydrogen. Peak at 2.4 min. is excess CO_2.

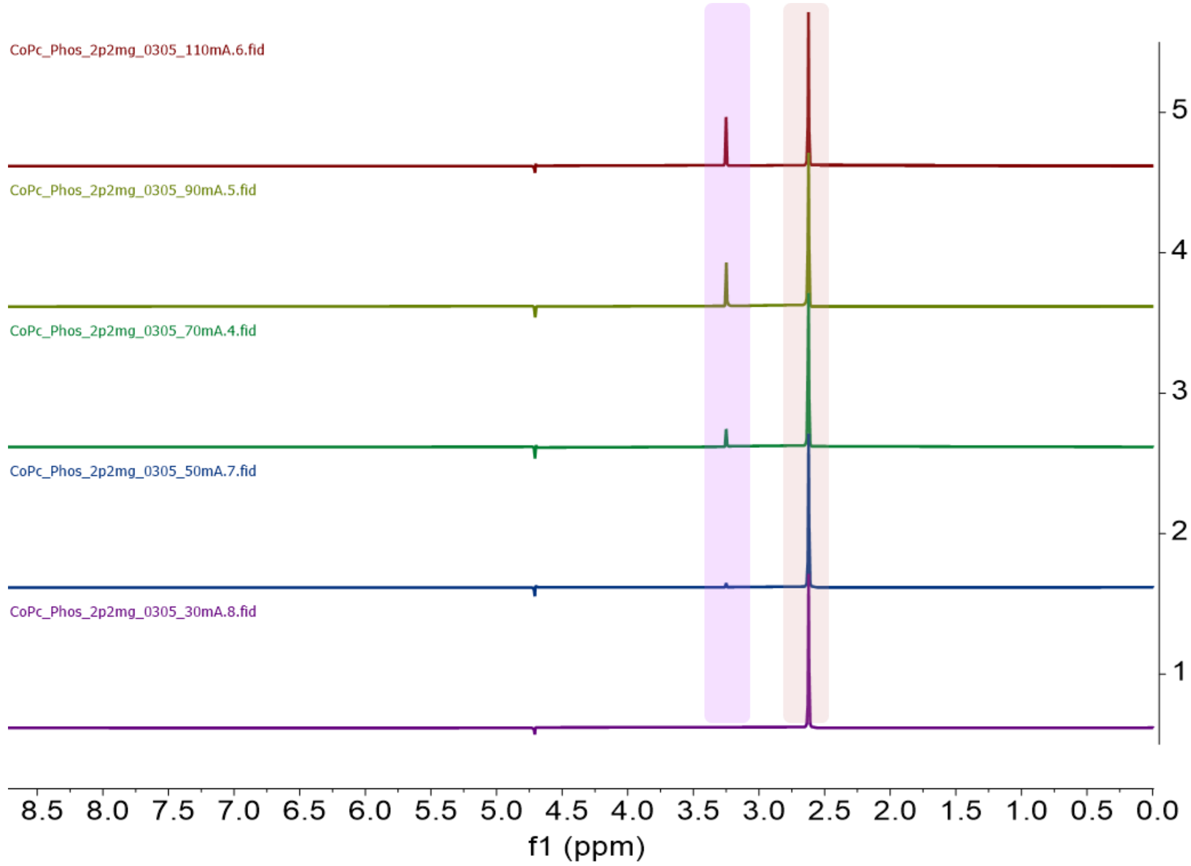

Fig. S35.
^{1}H NMR (600 MHz, D_2O) of CO_2RR electrolysis product in the flow cell at various applied currents. Singlet at 2.65 ppm (orange) is DMSO internal standard used for quantification. Singlet at 3.29 (highlighted in violet) is methanol product.

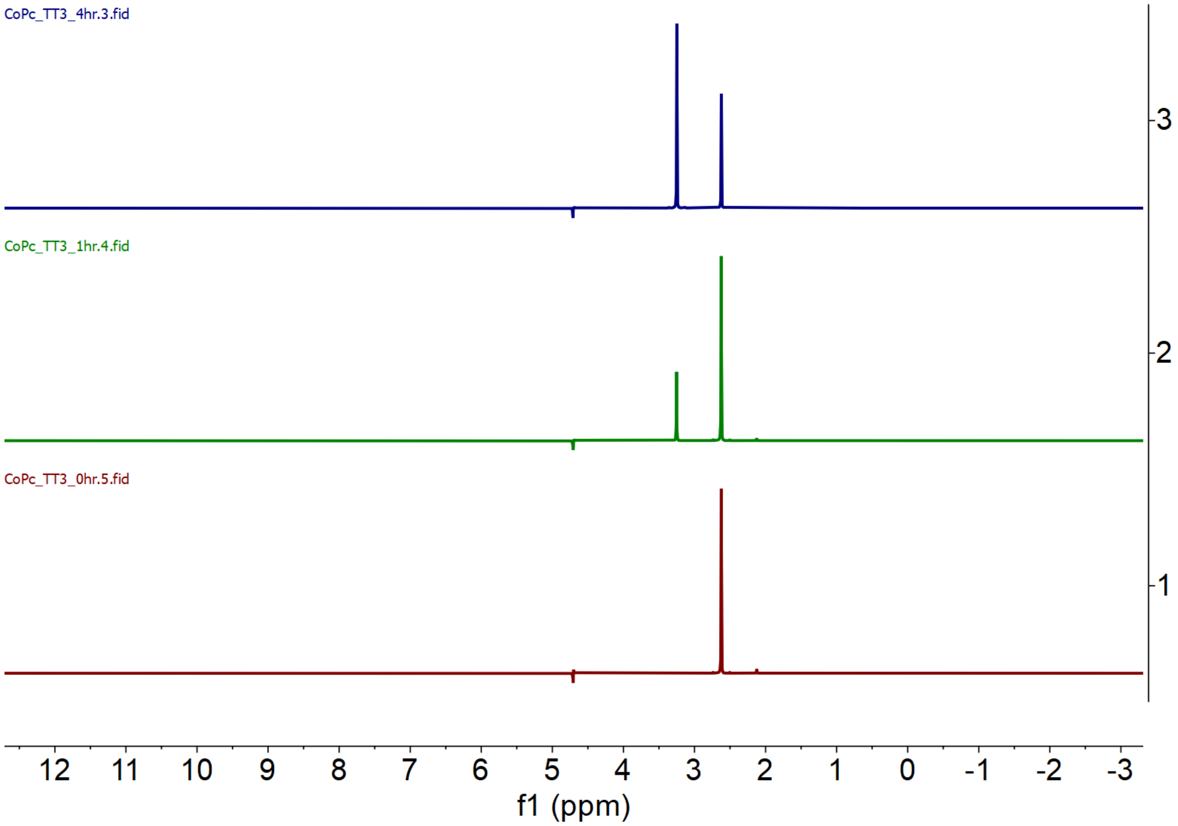

Fig. S36.
^{1}H NMR (600 MHz, D_2O) of 4-hour trial of CO_2RR at j_{tot} = 90 mA. Cumulative FE_{MeOH} was calculated to be 17% (final methanol concentration of 1 mM methanol).

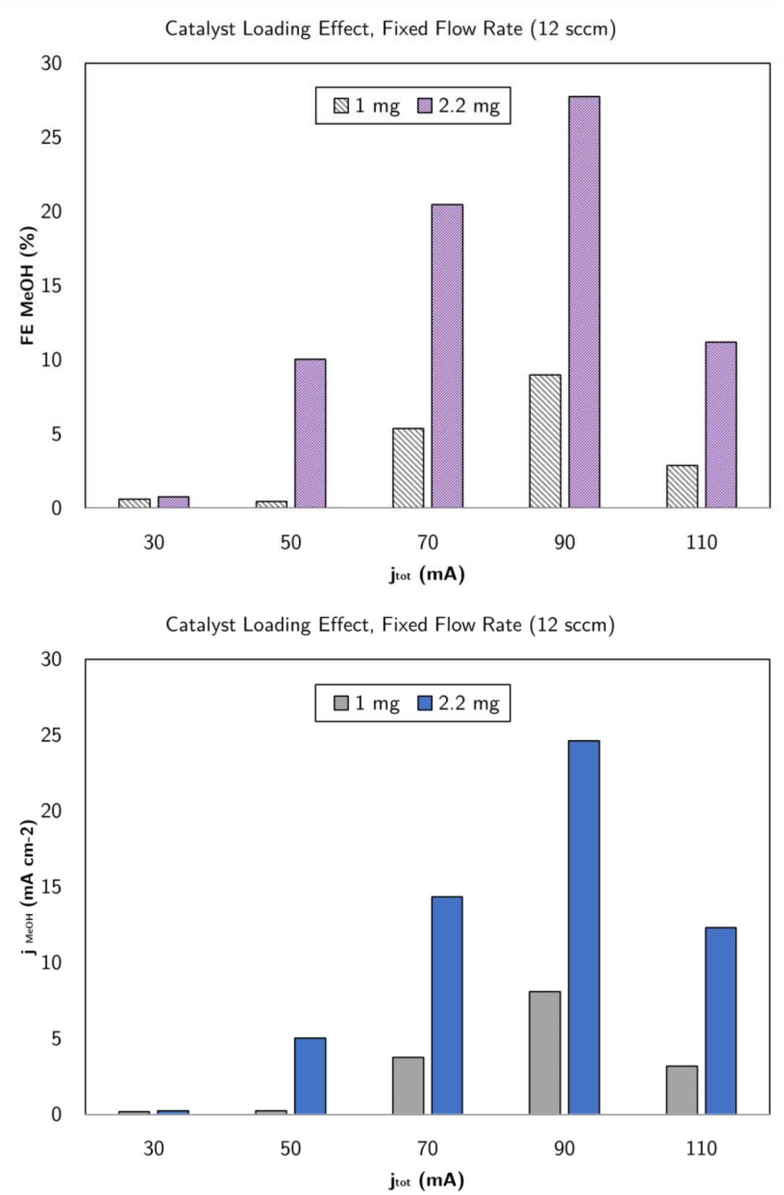

Fig. S37.
Flow cell results for CoPc/CNT total mass loading. Total electrode area: 4 cm^2 (1 cm^2 active area for electrolysis). Increasing total CoPc is hypothesized to improve the re-adsorption of desorbed CO product for cascadic reduction.

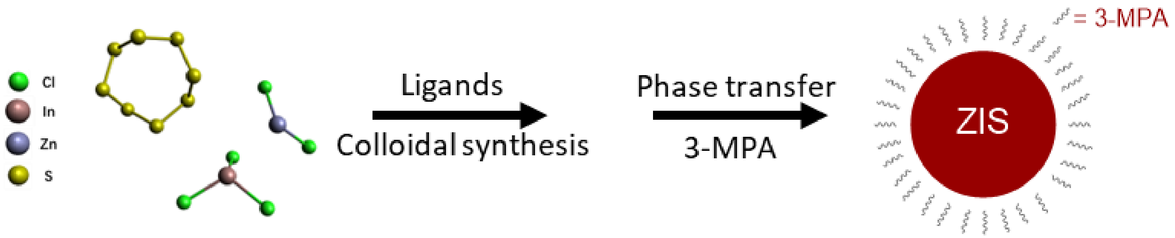

Fig. S38.
Colloidal synthesis scheme for ZIS NCs.

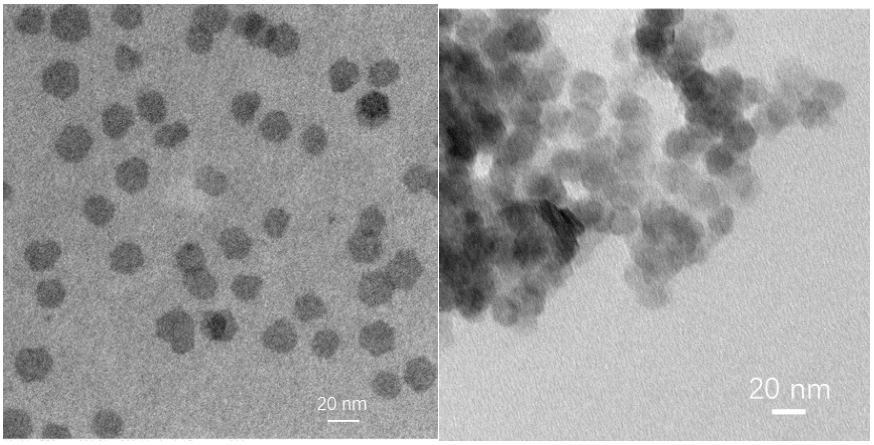

Fig. S39.
TEM image of pristine ZIS NCs (left) and ZIS NCs capped with 3-MPA ligand (right).

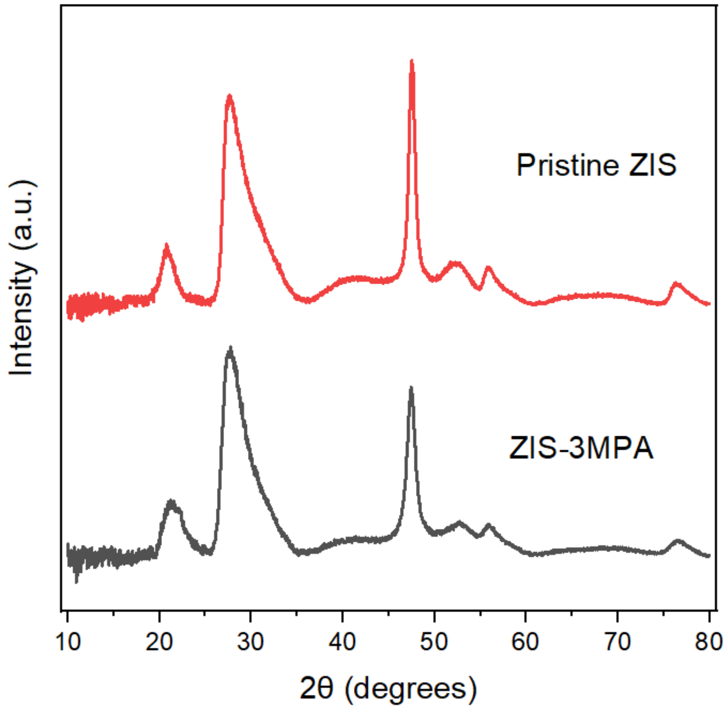

Fig. S40.
Powder XRD patterns of ZIS NCs before and after ligand exchange.

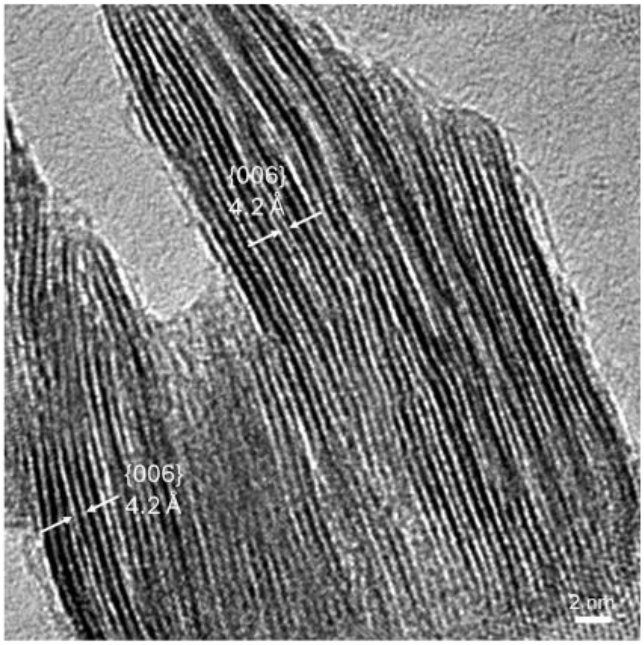

Fig. S41.
HRTEM showing ZIS plate stacking.

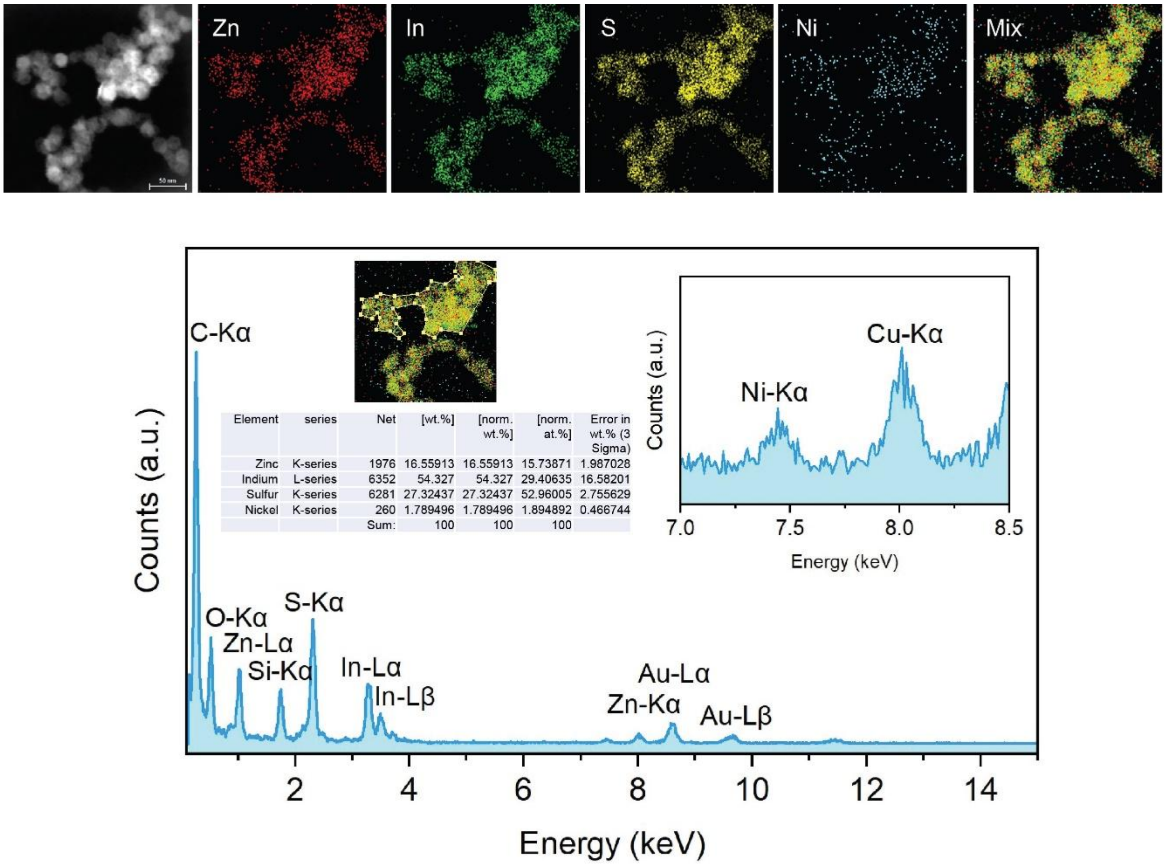

Element	series	Net	[wt.%]	[norm. wt.%]	[norm. at.%]	Error in wt.% (3 Sigma)
Zinc	K-series	1976	16.55913	16.55913	15.73871	1.987028
Indium	L-series	6352	54.327	54.327	29.40635	16.58201
Sulfur	K-series	6281	27.32437	27.32437	52.96005	2.755629
Nickel	K-series	260	1.789496	1.789496	1.894892	0.466744
		Sum:	100	100	100	

Fig. S42.
STEM-EDS spectral analysis of ZIS NCs loaded with Ni (see the STEM-EDS map inset). The Au signals are from the Au TEM grid. The Cu signals are from clips and washers used to fixate the grid on the TEM holder. Quantification: Cliff-Lorimer method.

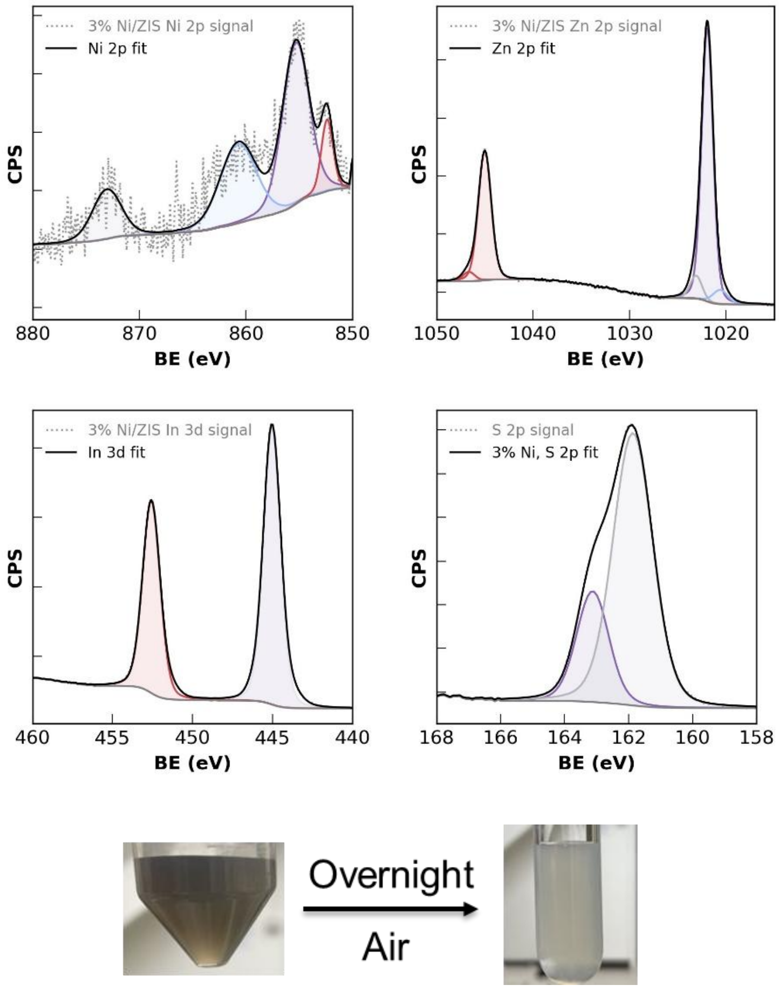

Fig. S43.
XPS spectra of Ni 2p, Zn 2p, In 3d, and S 2p of ZIS NCs after photodeposition (3 wt% Ni), along with the color change upon exposure to air, corresponding to the oxidation of Ni to NiO. It is believed that metallic Ni was the catalytically active site during photocatalytic dehydrogenation.

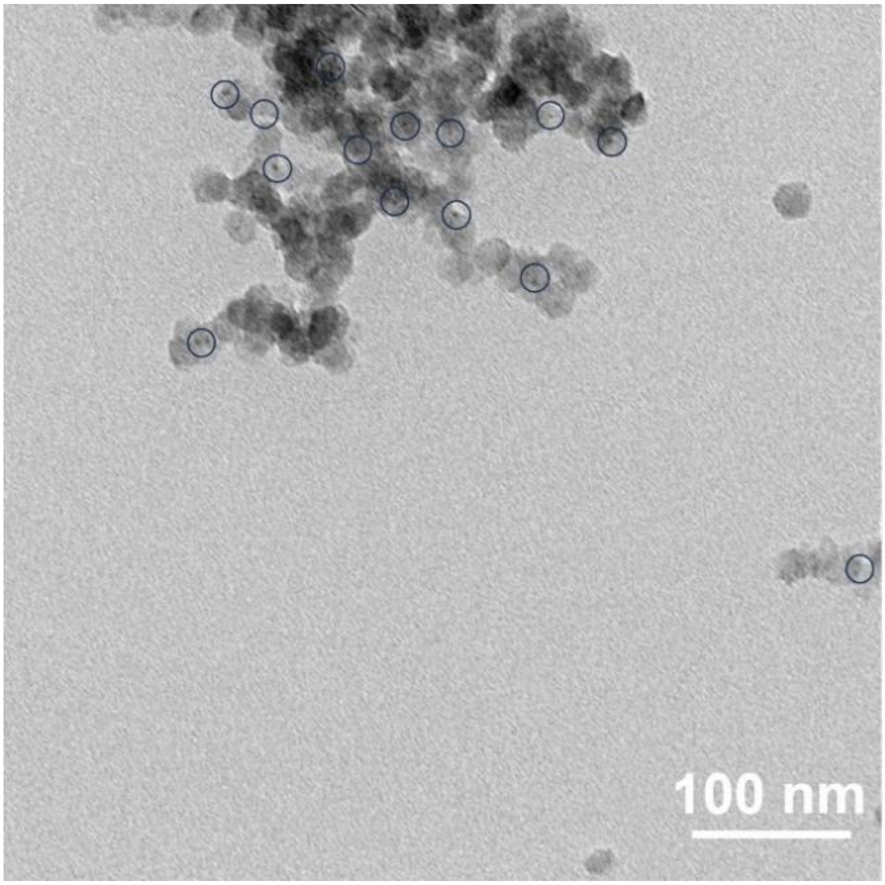

Fig. S44.
TEM images of ZIS NCs loaded with 5% Ni. The black spots marked in the image represent nickel particles.

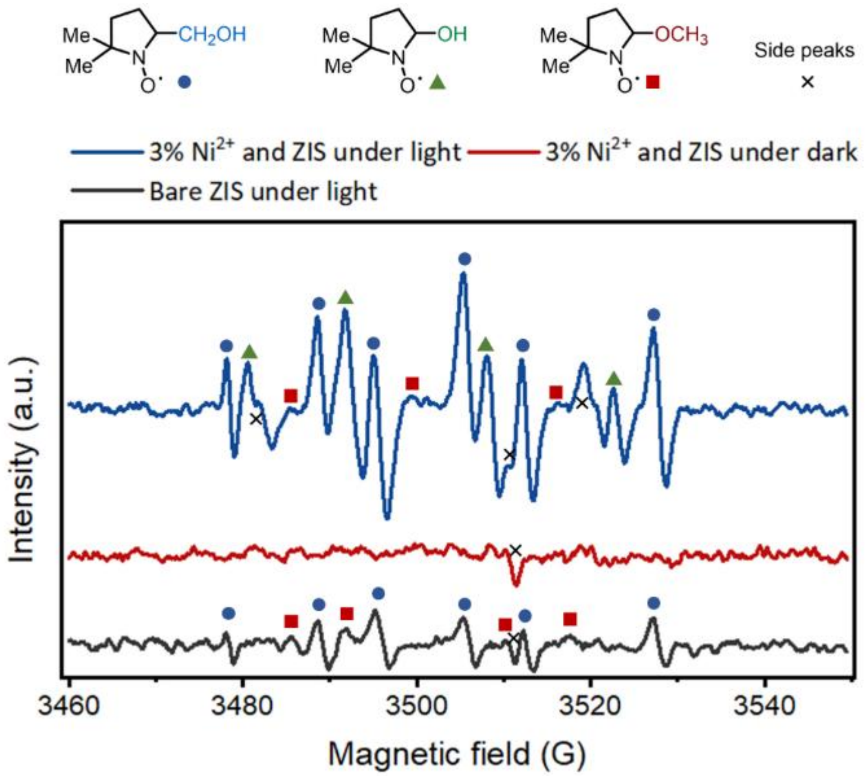

Fig. S45.
Electron paramagnetic resonance spectroscopy showing the generation of photochemically relevant intermediates on Ni/ZIS. In situ EPR experiments with and without Ni. Distinct radical species were detected in these two cases, suggesting the involvement of different reaction sites. The system with 3 wt% nickel exhibits a stronger signal intensity than bare ZIS, along with a markedly higher concentration of hydroxyl radical (HO·) in addition to $\cdot CH_2OH$. The generation of HO· implies that the Ni particles formed upon photodeposition might serve as reaction sites, which are prone to cleaving polar bonds. Side peaks include a background dip peak and likely DMPO-$O_2^{\cdot -}$ peaks.

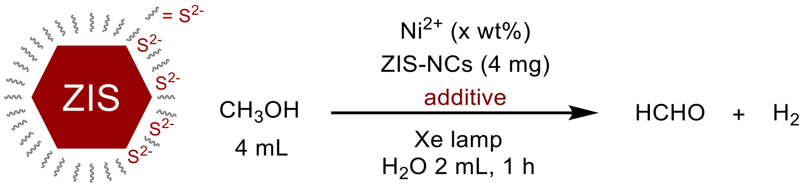

Entry	Catalyst	additive	HCHO mmol g^{-1} h^{-1}
1	Bare ZIS NCs	0.1 mmol $(NH_4)_2S_2O_8$	13.8
2	Bare ZIS NCs	none	3.8
3	ZIS NCs + 2 wt% Ni^{2+}	none	22.5

Fig. S46.
Control experiments. Control experiments conducted in the presence of an electron acceptor revealed a higher production rate of HCHO when $(NH_4)_2S_2O_8$ was used compared to bare ZIS (entries 1 and 2), suggesting that the hydrogen evolution reaction (HER) is the rate-limiting step in this photocatalytic process without a cocatalyst. These findings highlight the role of nickel in facilitating the HER. However, the production rate remains lower than that observed with the Ni cocatalyst (entries 1 and 3), indicating that nickel likely serves as an active site for methanol oxidation and may also contribute to charge separation, further enhancing the photocatalytic activity.

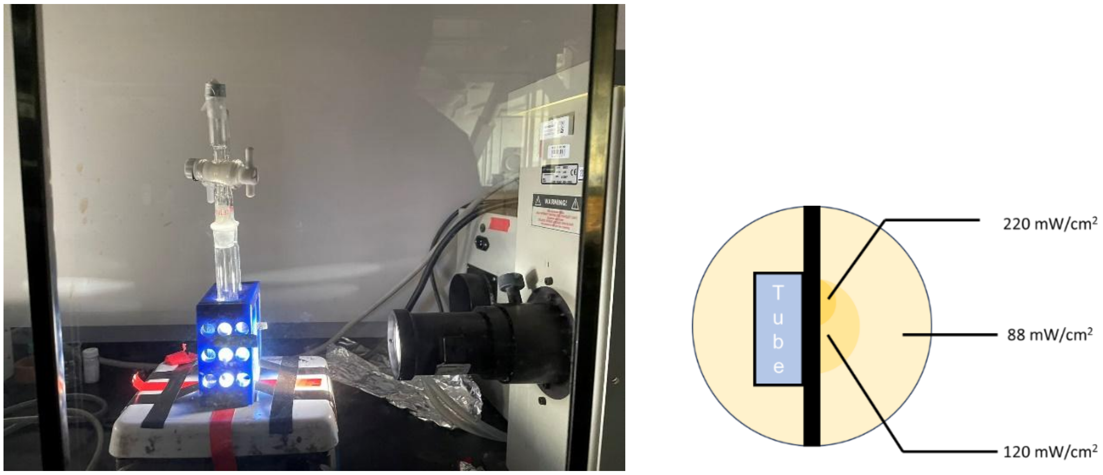

Fig. S47.
Image of photoreactor with Xe lamp setup and light intensity distribution.

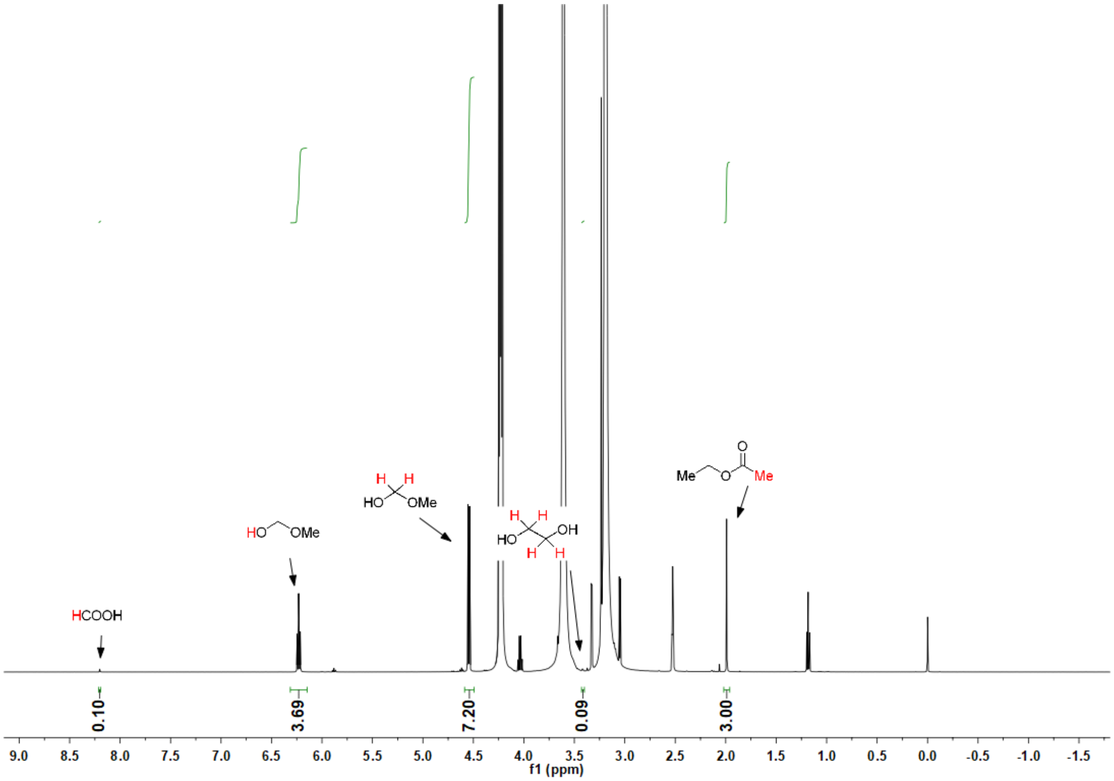

Fig. S48.
^1H NMR (500 MHz, DMSO-d_6) of crude reaction mixture after 24 h with 0.5 mmol ethyl acetate as the internal standard. Liquid phase product (HCHO): 0.21 mmol.

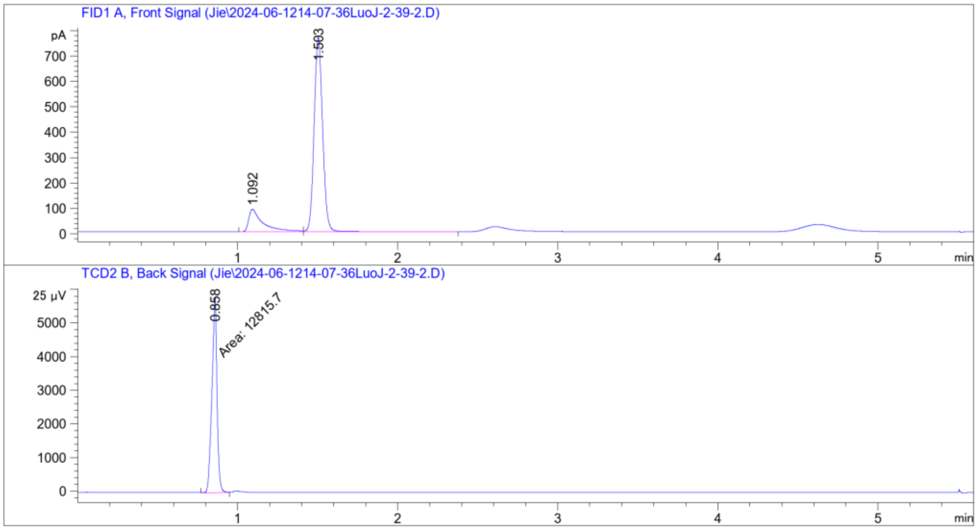

RetTime [min]	k'	Area [pA*s]	Height [pA]	Symm.	Width [min]	Plates	Resol ution	Signal /Noise
1.092	-	557.49799	88.93828	0.31	0.0750	1176	-	69.4
1.503	-	2828.82153	763.36993	0.87	0.0567	3899	3.67	595.8

RetTime [min]	k'	Area [25 µV*s]	Height [25 µV]	Symm.	Width [min]	Plates	Resol ution	Signal /Noise
0.858	-	1.28157e4	5755.08936	1.48	0.0330	3760	-	128.1

Fig. S49.
GC traces of gas product after 1 h under 370 nm LED (30 mW/cm^2),with background residual CO_2 and C_2H_4 peaks. n H_2 (TCD, retention time 0.858 min): 0.22 mmol. n CO (FID, retention time 1.092 min): 0.2 *μ*mol. n CH_4 (FID, retention time 1.503 min): 0.8 *μ*mol.

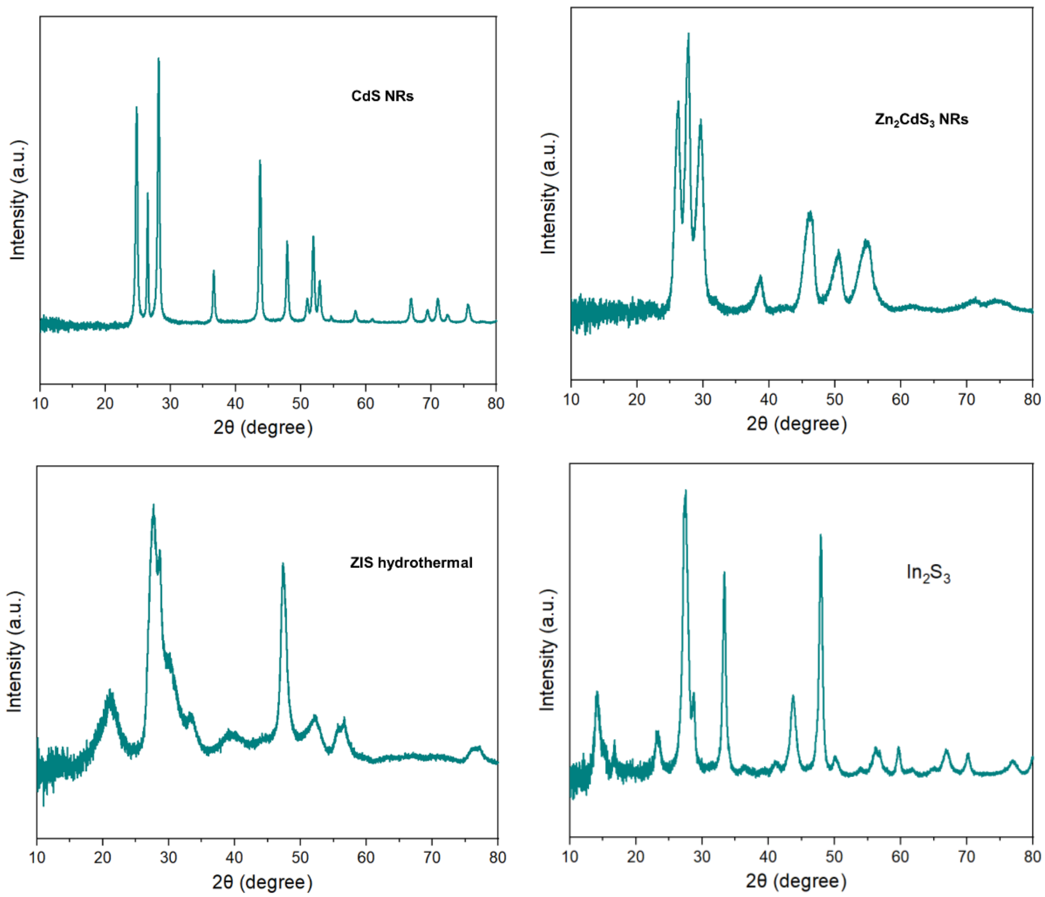

Fig. S50.
Powder XRD patterns of CdS NRs, Zn_2CdS_3 NRs, $Zn_2In_2S_5$-hydrothermal and In_2S_3.

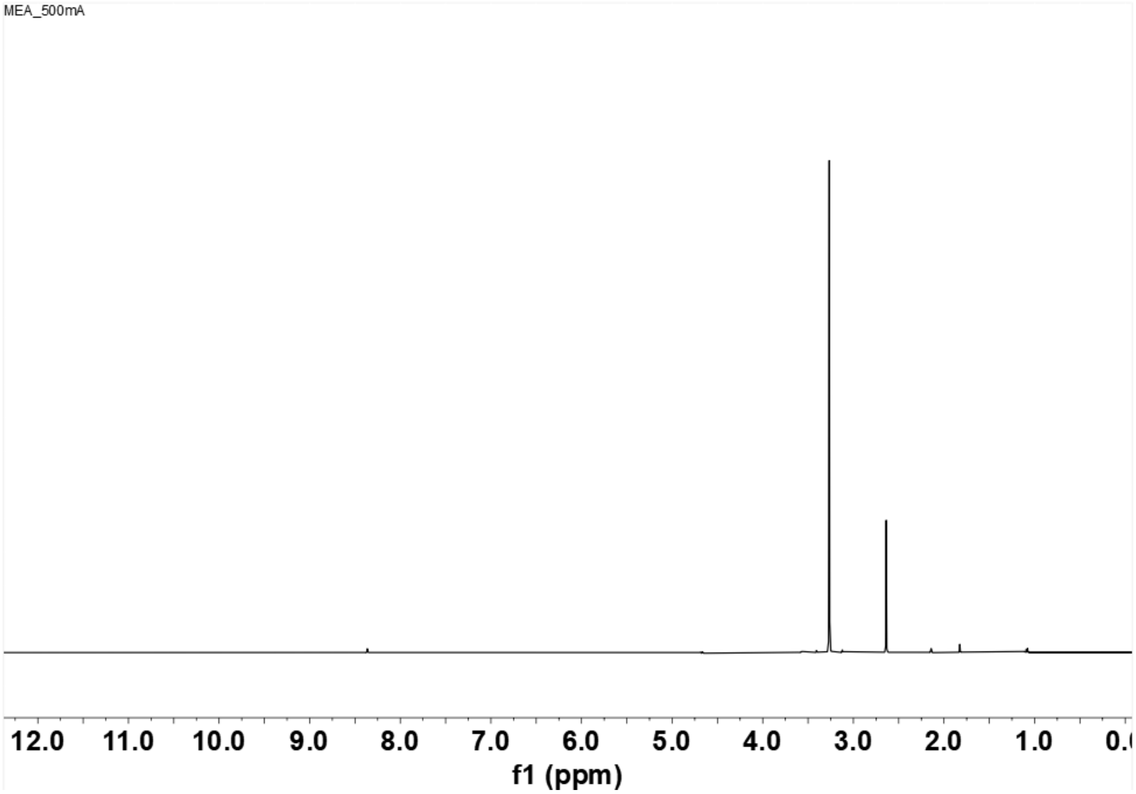

Fig. S51.
^{1}H-NMR of membrane electrode assembly-generated CO_2RR product. Singlet at 3.27 ppm is methanol, singlet at 2.64 ppm is DMSO internal standard.

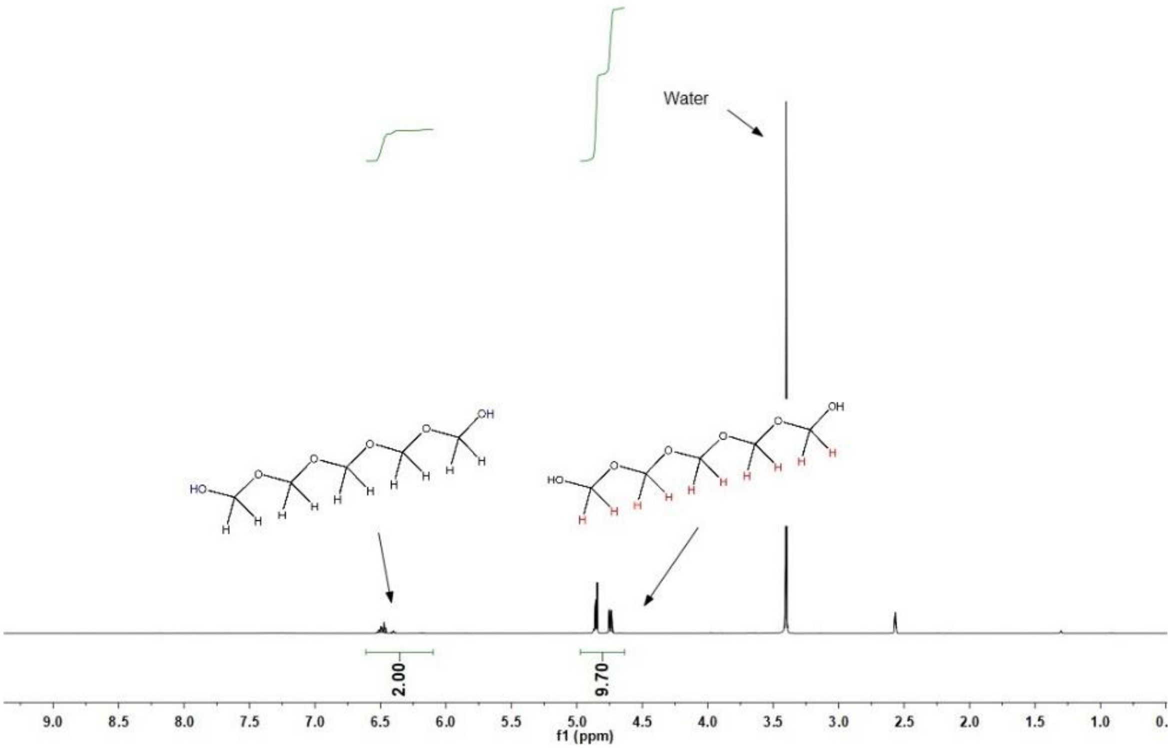

Fig. S52.
Isolated formaldehyde solid from the PMOR scale-up reaction, which was next dried and used for the formoin reaction. Based on the proposed linear structure of paraformaldehyde, degree of polymerization was estimated to be approximately n = 5.

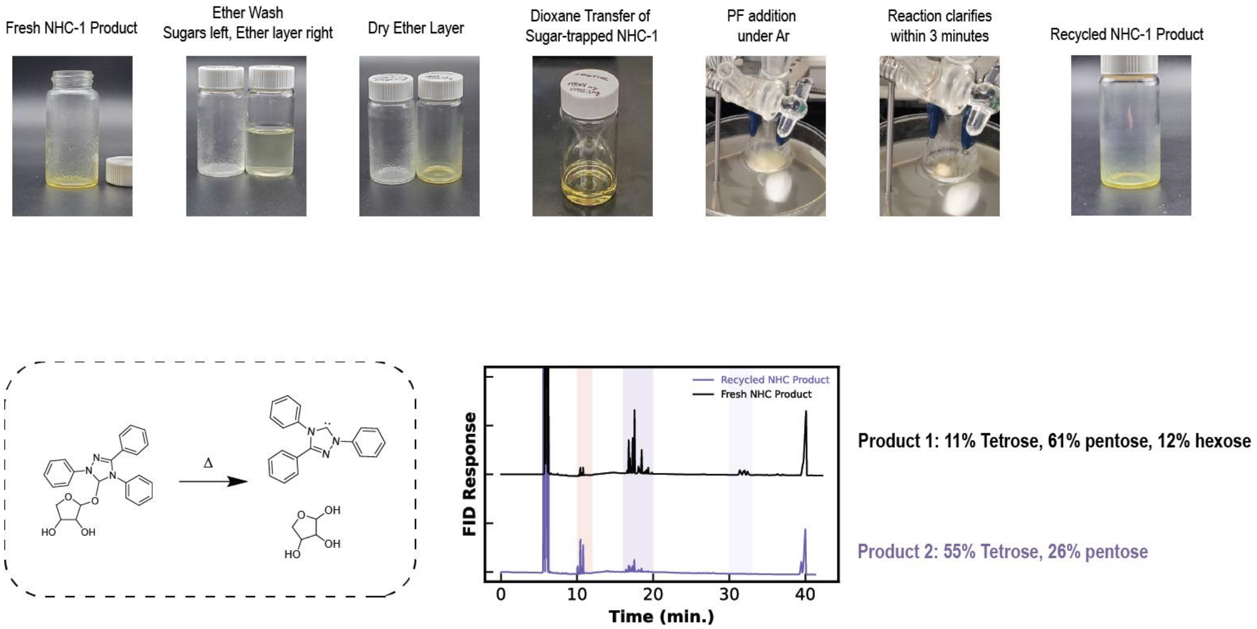

Fig. S53.
Recyclability of NHC-1 for formoin reaction. (top): recycling process post-formoin reaction, and addition to new substrate. (bottom): Illustration of protection mechanism that allows catalyst regeneration and product yields pre- and post-recycling. The diminished yield is likely a result of incomplete recovery of catalyst by a simple ether wash. The sugar product yields cannot be accounted for solely by transfer of adduct sugar from the NHC, indicating catalyst reusability.

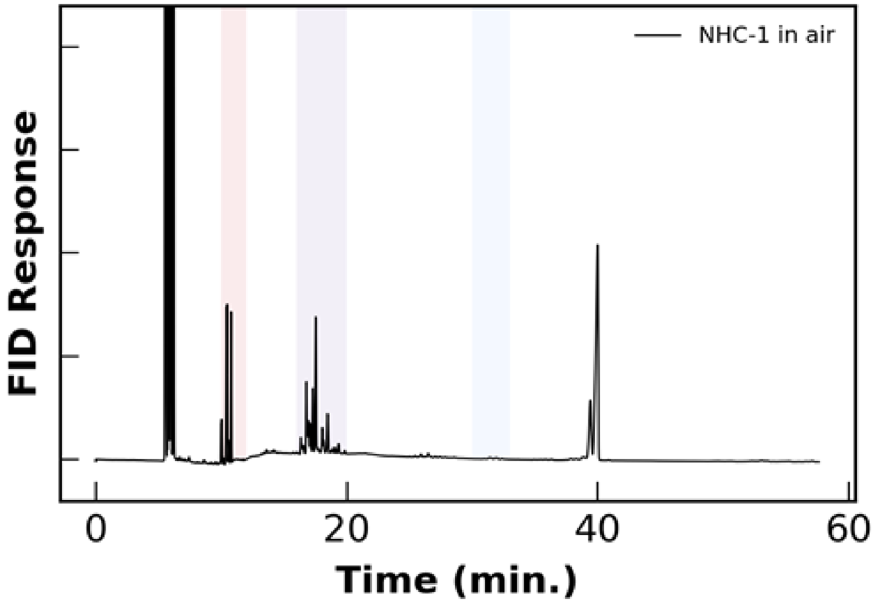

Fig. S54.
Formoin reaction performed in an open scintillation vial. Conditions: 2 mmol paraformaldehyde, 6 mg NHC-1, 5 mL dioxane, 100 °C, 15 minutes. Carbon yields were 65% tetrose, 38% pentose, with no hexose detected.

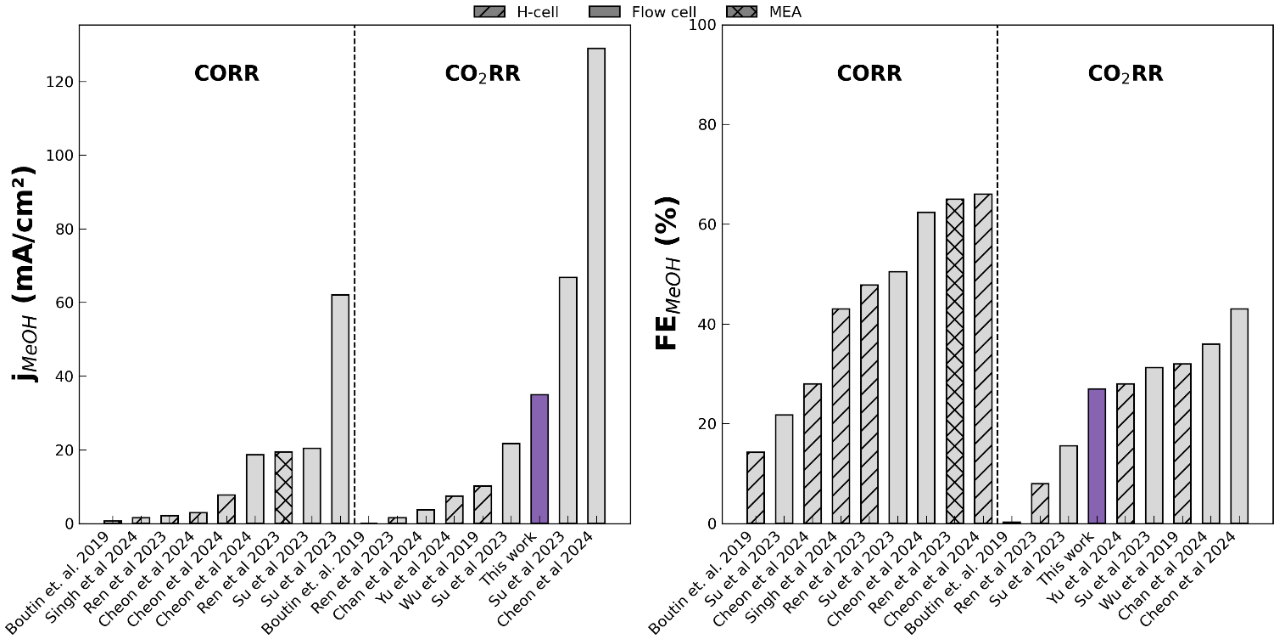

Fig. S55.
Highest reported partial current densities of (left) CORR and (right) CO_2RR from some prominent CoPc/CNT studies (9–15). **(right)** Highest reported Faradaic efficiencies from selected works. Note: in some cases, the highest reported FE is not from the same condition as the highest reported j_{MeOH}. Noteworthy is that CORR can generally be more electron efficient, as it eliminates CO as a possible faradaic product. However, this generally reduces the total operating current density.

Table S7: NHC-1 performance for short-time trials.

Time (min.)	n_{C3} (mmol as C_1)	n_{C4} (mmol as C_1)	n_{C5} (mmol as C_1)	n_{C6} (mmol as C_1)	TOF (h^{-1})
5	0	1.32	0.78	0	1600
15	0	1.02	0.85	0	532
30	0	0.46	1.55	0.13	266

To estimate the TOF for our typical formoin reactions using NHC-1, we used standard conditions of 2 mmol PF, 100 C, 5 mg NHC-1 (0.015 mmol), 5 mL dioxane. We ran the reaction for 5, 15, and 30 minutes. Each reaction was immediately quenched by cooling and addition of excess ethanol. The product was acetylated and quantified as previously described. Within analytical error, each reaction achieved quantitative conversion of formaldehyde, and thus the TOF is an apparent value:

$$TOF_{Total} = \frac{d(n_{carbon\ converted})}{dt \times n_{NHC}}$$

These figures indicate several notable observations: that the NHC-formoin reaction is quite fast, with nearly all formaldehyde substrate consumed within a few minutes at optimal conditions (yielding tetrose). Secondly, the product distribution shifts more toward pentose species at longer times, supporting the notion that they are the thermodynamic product and that some tetrose species may be degraded by the retro-benzoin reaction to build higher pentose and hexose species, albeit to a limited extent, and with lighter species (e.g. glycolaldehyde) that are not captured/resolved by this quenching and acetylation analysis strategy.

SI References

1. R. E. Blankenship, *et al.*, Comparing Photosynthetic and Photovoltaic Efficiencies and Recognizing the Potential for Improvement. *Science* **332**, 805–809 (2011).

2. S. Cestellos-Blanco, *et al.*, Toward abiotic sugar synthesis from CO2 electrolysis. *Joule* **6**, 2304–2323 (2022).

3. T. Cai, *et al.*, Cell-free chemoenzymatic starch synthesis from carbon dioxide. *Science* **373**, 1523–1527 (2021).

4. C. P. O'Brien, *et al.*, CO2 Electrolyzers. *Chem. Rev.* **124**, 3648–3693 (2024).

5. A. M. Bahmanpour, A. Hoadley, A. Tanksale, Critical review and exergy analysis of formaldehyde production processes. *Rev. Chem. Eng.* **30**, 583–604 (2014).

6. C. L. Rooney, *et al.*, Active Sites of Cobalt Phthalocyanine in Electrocatalytic CO2 Reduction to Methanol. *Angew. Chem. Int. Ed.* **63**, e202310623 (2024).

7. X. Ren, *et al.*, In-situ spectroscopic probe of the intrinsic structure feature of single-atom center in electrochemical CO/CO2 reduction to methanol. *Nat. Commun.* **14**, 3401 (2023).

8. Y. Yang, *et al.*, Operando probing dynamic migration of copper carbonyl during electrocatalytic CO2 reduction. *Nat. Catal.* **8**, 579–594 (2025).

9. J. Su, *et al.*, Strain enhances the activity of molecular electrocatalysts via carbon nanotube supports. *Nat. Catal.* **6**, 818–828 (2023).

10. E. Boutin, *et al.*, Aqueous Electrochemical Reduction of Carbon Dioxide and Carbon Monoxide into Methanol with Cobalt Phthalocyanine. *Angew. Chem.* **131**, 16318–16322 (2019).

11. A. Singh, *et al.*, Molecular Electrochemical Catalysis of CO-to-Formaldehyde Conversion with a Cobalt Complex. *J. Am. Chem. Soc.* **146**, 22129–22133 (2024).

12. X. Ren, *et al.*, In-situ spectroscopic probe of the intrinsic structure feature of single-atom center in electrochemical CO/CO2 reduction to methanol. *Nat. Commun.* **14**, 3401 (2023).

13. S. Cheon, J. Li, H. Wang, In Situ Generated CO Enables High-Current CO2 Reduction to Methanol in a Molecular Catalyst Layer. *J. Am. Chem. Soc.* (2024). https://doi.org/10.1021/jacs.4c05961.

14. S. Yu, *et al.*, CO2-to-methanol electroconversion on a molecular cobalt catalyst facilitated by acidic cations. *Nat. Catal.* 1–10 (2024). https://doi.org/10.1038/s41929-024-01197-2.

15. Y. Wu, Z. Jiang, X. Lu, Y. Liang, H. Wang, Domino electroreduction of CO2 to methanol on a molecular catalyst. *Nature* **575**, 639–642 (2019).
